# Genetic and functional correction of argininosuccinate lyase deficiency using CRISPR adenine base editors

**DOI:** 10.1016/j.ajhg.2024.03.004

**Published:** 2024-04-04

**Authors:** Sami Jalil, Timo Keskinen, Juhana Juutila, Rocio Sartori Maldonado, Liliya Euro, Anu Suomalainen, Risto Lapatto, Emilia Kuuluvainen, Ville Hietakangas, Timo Otonkoski, Mervi E. Hyvönen, Kirmo Wartiovaara

**Affiliations:** 1 Stem Cells and Metabolism Research Program, Faculty of Medicine, University of Helsinki, Helsinki, Finland; 2 Faculty of Biological and Environmental Sciences University of Helsinki, Helsinki, Finland; 3 Clinical Genetics, Helsinki University Hospital, Helsinki, Finland; 4 Institute of Biotechnology, Helsinki Institute of Life Science, University of Helsinki, Helsinki, Finland; 5 New Children’s Hospital, Pediatric Research Center, University of Helsinki and Helsinki University Hospital, Helsinki, Finland

**Keywords:** CRISPR, genome editing, ABE, lipid nanoparticles, LNP, mRNA, genetic defect, liver, argininosuccinic aciduria, ASL

## Abstract

Argininosuccinate lyase deficiency (ASLD) is a recessive metabolic disorder caused by variants in *ASL*. In an essential step in urea synthesis, ASL breaks down argininosuccinate (ASA), a pathognomonic ASLD biomarker. The severe disease forms lead to hyperammonemia, neurological injury, and even early death. The current treatments are unsatisfactory, involving a strict low-protein diet, arginine supplementation, nitrogen scavenging, and in some cases, liver transplantation. An unmet need exists for improved, efficient therapies. Here, we show the potential of a lipid nanoparticle-mediated CRISPR approach using adenine base editors (ABEs) for ASLD treatment. To model ASLD, we first generated human-induced pluripotent stem cells (hiPSCs) from biopsies of individuals homozygous for the Finnish founder variant (c.1153C>T [p.Arg385Cys]) and edited this variant using the ABE. We then differentiated the hiPSCs into hepatocyte-like cells that showed a 1,000-fold decrease in ASA levels compared to those of isogenic non-edited cells. Lastly, we tested three different FDA-approved lipid nanoparticle formulations to deliver the ABE-encoding RNA and the sgRNA targeting the *ASL* variant. This approach efficiently edited the *ASL* variant in fibroblasts with no apparent cell toxicity and minimal off-target effects. Further, the treatment resulted in a significant decrease in ASA, to levels of healthy donors, indicating restoration of the urea cycle. Our work describes a highly efficient approach to editing the disease-causing *ASL* variant and restoring the function of the urea cycle. This method relies on RNA delivered by lipid nanoparticles, which is compatible with clinical applications, improves its safety profile, and allows for scalable production.

## Graphical abstract

**Figure:**
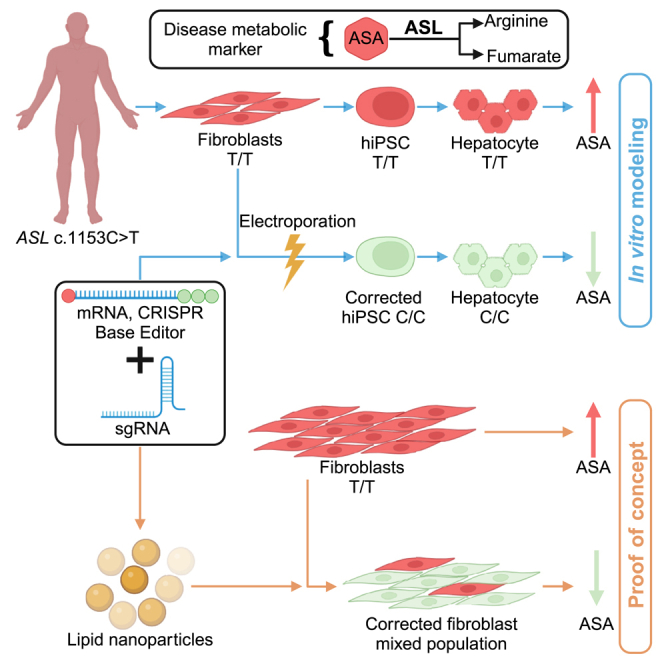

## Teaser

Harnessing CRISPR to correct a metabolic inherited disease, we developed a therapeutically relevant method to precisely edit the Finnish Founder pathogenic variant responsible for argininosuccinate lyase deficiency. By lipid-nanoparticle-mediated delivery of the adenosine base editor RNA, we achieve efficient gene editing and metabolic normalization in patient fibroblasts.

## Introduction

Argininosuccinate lyase deficiency (ASLD [MIM: 207900]), also known as argininosuccinic aciduria, is an autosomal-recessive urea cycle disorder caused by loss-of-function variants in *ASL* (MIM: 608310).^1^^,^^2^^,^^3^ ASLD, like other urea cycle defects, impairs the disposal of nitrogen and leads to hyperammonemia. It can present as a neonatal life-threatening condition with lethargy, vomiting, seizures, and coma. These symptoms can also arise later in life during a catabolic state. The long-term sequelae include epilepsy, developmental impairment, and liver disease.^1^^,^^2^^,^^3^ The incidence of ASLD in the US and Europe has been estimated in the most recent reports to be from 1:190,000 to 1:219,000 live births,^4^^,^^5^ and in Finland 1:144,000.^6^

The ASL enzyme catalyzes the breakdown of argininosuccinate (ASA) into arginine and fumarate.^7^ This is an essential cytosolic step in the urea cycle and its disruption leads to a potentially toxic ASA accumulation.^2^^,^^8^ Moreover, the disruption of ASL activity impairs the biosynthesis of arginine, which becomes an essential amino acid that needs to be acquired through diet.^2^ The hallmark of ASLD, and a diagnostically helpful distinction from other urea cycle defects, is the accumulation of ASA in plasma and urine.^9^

The pathophysiology of ASLD remains partially unclear. It involves hyperammonemia causing irreversible damage to the developing central nervous system,^10^ the potential hepatic and neurotoxic effects of ASA and its related metabolite guanidinosuccinate,^2^^,^^8^^,^^11^^,^^12^ and the compromised nitric oxide (NO) synthesis.^13^^,^^14^ ASLD variants disrupt NO synthesis through two mechanisms: they interfere with the structural role of ASL in the multiprotein complex required for NO production and impair the catalytic activity of ASL, restricting the recycling of citrulline into the arginine supply available for NO synthesis.^13^ The current options for disease management are a protein-restricted diet combined with arginine supplementation and nitrogen-scavenging medications or liver transplantation in severe cases.^2^

The disease severity varies depending on the variant and the residual ASL enzyme activity.^15^ The Finnish founder *ASL* variant (c.1153C>T [GenBank: NM_000048.4] [p.Arg385Cys]; rs28940286) is enriched in Finland, and according to the gnomAD database, the allele frequency is 0.0004832. This variant is homozygous or compound heterozygous in 70% of the identified ASLD individuals in Finland and results in a cysteine replacing the positively charged arginine at a crucial position near the enzyme’s active site. This arginine interacts with the negatively charged glutamine 389 to stabilize the carboxy-terminus helix bundle of the protein.^16^ Substitutions in arginine 385 may also interfere with residues near the active site, such as glutamine 399, thus impairing enzyme activity.^16^ The enzyme activity of this variant is not exactly known and can depend on the assay used, but a direct ASL enzyme activity assay in fibroblasts showed no activity.^17^^,^^18^

The CRISPR-Cas9 base editors have emerged as powerful tools for precisely editing point mutations with high efficiency and collectively possess the potential to reverse up to 60% of all pathogenic point mutations.^19^ The adenine base editor (ABE)^20^ catalyzes the nucleotide transition of a targeted A-T to a G-C base pair within a programmable and narrow target window without requiring a DNA donor template nor inducing DNA double-strand breaks, thereby providing a safer alternative to approaches that rely on such breaks and carry a higher risk of genomic rearrangements, insertions, and deletions.^21^ The ABE is not only safer, but also maintains a very high on-target efficiency while presenting a lower off-target activity than that observed with canonical SpCas9.^21^^,^^22^^,^^23^ Animal and pre-clinical studies have demonstrated the promise of base editors in editing variants associated with diseases, such as Duchenne muscular dystrophy (MIM: 310200)^24^ and hereditary tyrosinemia type 1 (MIM: 276700).^25^ Similarly, ongoing clinical trials are already underway to assess their therapeutic potential in individuals with familial hypercholesterolemia, lymphoblastic leukemia, and sickle cell disease (ClinicalTrials.gov: NCT05398029, NCT05885464, and NCT05456880).

We hypothesized that CRISPR base editors could offer a potential therapy for ASLD and tested this approach *in vitro* with the Finnish founder *ASL* variant c.1153C>T. By reprogramming ASLD fibroblasts into human-induced pluripotent stem cells (hiPSCs) and simultaneously editing the variant, we rescued the disrupted urea cycle in the genetically edited hiPSC-derived hepatocyte-like cells. To enable *in vivo* delivery, we encapsulated an optimized mRNA construct encoding the improved ABE version, ABE8e,^26^ along with the variant-targeting single-guide RNA (sgRNA), into lipid nanoparticles (LNPs). We tested three different US Food and Drug Administration (FDA)-approved LNP formulations,^27^^,^^28^^,^^29^ similar to those employed in COVID-19 mRNA vaccines, and they successfully delivered the ABE8e mRNA construct into the fibroblasts, editing the *ASL* variant, rescuing the enzyme activity, and restoring ASA levels to that of healthy donors without any evident toxicity or off-target effects.

## Material and methods

### Ethical permit

Generation of fibroblast and hiPSC lines from skin biopsies was approved by the Coordinating Ethics Committee of the Helsinki and Uusimaa Hospital District upon informed consent of the donors or their guardians (diary no.: HUS/2754/2019).

### Biopsies

Skin biopsy samples were collected from two unrelated voluntary donors carrying the Finnish founder variant *ASL* variant (c.1153C>T [p.Arg385Cys]) in homozygosity. The child’s biopsy was taken under general anesthesia for an unrelated reason.

### Development of T3_ABE8e_IVT plasmid

ABE8e was a gift from David Liu (Addgene: 138489; http://n2t.net/addgene:138489; RRID: Addgene_138489). We cloned the ABE8e open reading frame into a backbone containing a T3 promoter for *in vitro* transcription, and *Xenopus* 5′ and 3′ UTRs, as we previously did for the ABEmax.^30^ For the cloning, we used the NEB HighFidelity Assembly kit (New England BioLabs, catalog no.: E5520S), primers are detailed in Table S3.

Our plasmids for T3 *in vitro* transcription of the ABE8e and the ABEmax were deposited in Addgene (plasmids 201676 and 171761).

### *In vitro* transcription ABEmax, and ABE8e

Employing the ABEmax or the ABE8e IVT plasmid (Addgene: 201676 and 171761) as a DNA template, T3 RNA transcription was performed according to the manufacturer’s protocol (mMESSAGE mMACHINE T3 Transcription Kit, Thermo Fisher Scientific, Invitrogen, catalog no.: AM1348). The plasmid was linearized by SfiI restriction (Thermo Fisher Scientific, catalog no.: FD1824).

### Fibroblast reprogramming and simultaneous ABEmax-mediated editing

Fibroblast electroporation, reprogramming, ABEmax editing, and hiPSC line generation were performed as previously described.^30^

### Hepatocyte differentiation

Before hepatocyte differentiation, we cultured hiPSCs in Essential 8 medium (E8, Thermo Fisher Scientific, A1517001). On the day before the start of the differentiation, cells were treated with 0.5 mM EDTA in PBS, resuspended into single cell with DMEM, and seeded onto Matrigel-coated (Corning, 356231) 12-well plates (800,000 cells per well) containing E8 and 10 μM rho-associated protein kinase (ROCK) inhibitor (Y-27632, Selleckchem). On day 0, we changed the medium to a definitive endoderm induction medium consisting of Basal 1 media (MCDB 131 free from L-Glutamine [PAN BIOTECH, P04-80057] supplemented with 5 mg/mL Bovine Serum Albumin [Sigma, A7030], 1.5 mg/mL NaHCO_3_ [Sigma], Glucose and Glutamax with 0.1 μg/mL Activin A [Qkine, Qk001], and 3 μM CHIR-99021 [Tocris, 4423]). On day 1, we changed the medium to Basal 1 media with 0.1 μg/mL activin A and 0.3 μM CHIR. On day 2, we changed the medium to Basal 1 media with 0.1 μg/mL activin A and estimated the definitive endoderm induction efficiency by flow cytometry using BD PharmingenTM PE Mouse Anti-Human CD184 (#555974) to stain a definitive endoderm marker CXCR4 and BD PharmingenTM PE Mouse IgG1 (#555574) as a control. We proceeded with the differentiation if the cell population reached a threshold of 80% CD184-positive cells at this stage. From day 3 until day 18, we followed the hepatocyte differentiation protocol previously described.^31^

### LNP formulation

LNPs were generated by mixing an organic phase and an aqueous phase in a volume ratio of 1:2 using the NanoAssemblr Spark (Precision Nanosystems) according to the vendor’s instruction. These two phases mix within the microfluidic channels of the cartridge to form LNPs. The LNPs were immediately dispersed in a volume of neutral PBS buffer equal to the sum of the two input phases.

The organic phase consisted of a 45 mM mix of different lipids in ethanol according to the LNP formulations.

#### ALN-18328 (Onpattro/patisiran)

The solution consisted of DLin-MC3-DMA/DSPC/Cholesterol/DMG-PEG2000 (50/10/38.5/1.5 mol/mol). DLin-MC3-DMA (MedKoo Biosciences Inc. CAT#: 555308), DSPC (Avanti Polar Lipids CAT#: 850365), Cholesterol (Sigma-Aldrich C3045), DMG-PEG 2000 (Sigma-Aldrich 880151P). Molar N/P: 3. The mixture was prepared as previously described.^28^

#### mRNA-1273 (Moderna)

The solution consisted of SM-102/DSPC/Cholesterol/DMG-PEG2000 (50/10/38.5/1.5 mol/mol). SM-102 (BroadPharm CAT#: BP-25499). Molar N/P: 6. The mixture was prepared as previously described.^29^^,^^32^

#### BNT162b2 (Pfizer-BioNTech)

The solution consisted of ALC-0315/DSPC/Cholesterol/ALC-0159 (46.3/9.4/42.7/1.6 mol/mol). ALC-0315 (MedKoo Biosciences Inc. CAT#: 556006), ALC-0159 (BroadPharm CAT#: BP-25711). Molar N/P: 6. The mixture was prepared as previously described.^29^^,^^32^

The aqueous phase consists of sodium citrate buffer (pH 4, 65 mM) containing the sgRNA (IDT, Integrated DNA Technologies) and the ABE8e RNA construct in a 1:2 mass ratio.

Independently of the formulation, the total RNA concentration in the resulting LNPs was 17 mg/ml, considering the mass of the RNA construct and the sgRNA.

### Metabolomics analysis

Samples were analyzed on a Thermo Q Exactive Focus Quadrupole Orbitrap mass spectrometer coupled with a Thermo Dionex UltiMate 3000 HPLC system (Thermo Fisher Scientific). The high-performance liquid chromatography (HPLC) was equipped with a hydrophilic ZIC-pHILIC column (150 × 2.1 mm, 5 μm) with a ZIC-pHILIC guard column (20 × 2.1 mm, 5 μm, Merck Sequant). A 5 μL sample was injected into the liquid chromatography-mass spectrometry (LC-MS) instrument after quality controls in randomized order having every tenth sample as blank. A linear solvent gradient was applied in decreasing organic solvent (80%–35%, 16 min) at 0.15 mL min–1 flow rate and 45°C column oven temperature. Mobile phases were aqueous 200 mmol per liter ammonium bicarbonate solution (pH 9.3, adjusted with 25% ammonium hydroxide), 100% acetonitrile, and 100% water. Ammonium bicarbonate solution was kept at 10% throughout the run, resulting in a steady 20 mmol per liter concentration. Metabolites were analyzed using a mass spectrometer with a heated electrospray ionization source using polarity switching and the following settings: resolution of 70,000 at m/z of 200; spray voltages of 3,400 V for positive and 3,000 V for negative mode; sheath gas of 28 arbitrary units (AU) and auxiliary gas of 8 AU; vaporizer temperature of 280°C; and ion transfer tube temperature of 300°C. The instrument was controlled using Xcalibur 4.1.31.9 software (Thermo Scientific). Metabolite peaks were confirmed using commercial standards (Sigma-Aldrich). Data quality was monitored throughout the run using an in-house quality control cell line extracted similarly to other samples. After final peak integration with TraceFinder 4.1 SP2 software (Thermo Scientific), peak area data were exported as Excel files. The absolute peak area of a metabolite of interest was normalized to the sum of the absolute peak areas of all metabolites in the same sample. See Table S4 for metabolomics raw data and calculations.

### Data analysis

The data from the different software employed were collected in tables and analyzed using R scripts. For multiple comparisons, we used ANOVA coupled with a post hoc Tukey test. Data are represented as the mean ± SEM.

## Results

### Clinical characteristics of individuals homozygous for the *ASL* variant c.1153C>T

Two unrelated individuals with homozygous *ASL* c.1153C>T variant donated skin biopsies for fibroblast culture.

Person 1 (Table 1) was diagnosed by newborn screening while having hyperammonemia at the time of the diagnosis with relatively mild symptoms. The diagnosis was based on elevated ASA in plasma and urine and was confirmed by genetic testing. Treatment was initiated at 8 days of age, and hyperammonemia resolved rapidly. The individual is on a protein-restricted diet, arginine supplementation, and nitrogen-scavenging medication. After the diagnosis, the person has had one mild hyperammonemic episode and has a developmental delay. The skin biopsy was taken at 18 months of age.

**Table 1:** Clinical characteristics of individuals homozygous for the *ASL* variant c.1153C>T

	**Type of diagnosis**	**Age at diagnosis**	**Confirmation of diagnosis**		**ASA (umol/l), not detected normally**	**Citrulline (umol/l), normal <50**	**Arginine (umol/l), normal >10**	**Glutamine (umol/l), normal <800**	**Ammonium (umol/l), normal <100 in newborns, <50 later**	**ALT (U/l), normal <50**
Person 1	screening	8 days	sequencing	laboratory tests at diagnosis	626	397	23	1,442	314	37
				laboratory tests at follow up	219 (75–446)	175 (84–260)	–	–	46 (10–140)	73 (22–195)
Person 2	symptomatic	10 months	enzyme activity test in erythrocytes: 0.18 μmol/h/g Hb (reference range in the laboratory 5–8 μmol/h/g Hb)	laboratory tests at diagnosis	N/A (high in urine)	149	21	896	190	621
				laboratory tests at follow up	194 (103–291)	214 (97–356)	–	–	61 (39–126)	71 (16–305)

Table presenting clinical data of the two individuals. Plasma amino acids, ammonium, and alanine aminotransferase (ALT) were analyzed in Helsinki University Hospital Laboratory HUSLAB. The reference ranges for amino acids vary according to age; the reference values presented are the rounded mean. The follow-up data from 10 recent years are presented as the mean, and in between parentheses, the lowest and the highest values.

Person 2 (Table 1) was diagnosed at 10 months of age when presenting hyperammonemic symptoms: lethargy and vomiting. Before that, the person showed signs of motor developmental delay. The diagnosis was based on elevated ASA excretion in urine, and a typical plasma amino acid profile of high citrulline and low arginine and was confirmed by ASL enzyme activity measurement in erythrocytes. The genetic testing was done later. The individual has been on a protein-restricted diet and arginine supplementation since the diagnosis, and nitrogen scavengers have been added to the therapy. The person has had several mild to moderate hyperammonemic episodes and has an intellectual disability. The skin biopsy was taken at 30 years of age.

### Generation of hepatocyte-like cells from edited and not edited proband-derived hiPSCs to study ASLD

To generate a disease-relevant cell type, we applied a stem cell approach. We edited the pathogenic variant and simultaneously reprogrammed fibroblast derived from two individuals homozygous for the *ASL* variant into hiPSC by electroporating the ABEmax^33^ mRNA, a single-guide RNA, and plasmids expressing classic reprogramming factors as previously described.^30^ The average on-target A-T to G-C base editing efficiency reached 30% in this reaction, as the targeted base is in the suboptimal ninth position in the editing window.^20^ The subsequent experiments were performed using two edited hiPSC lines (*ASL* c.1153 C/C) and two non-edited hiPSC lines (*ASL* c.1153 T/T) from each individual. The quality controls demonstrated pluripotency (Figure S1), genomic integrity (Figure S2), and the absence of off-target effects (Table S1) for all cell lines.

**Figure 1:**
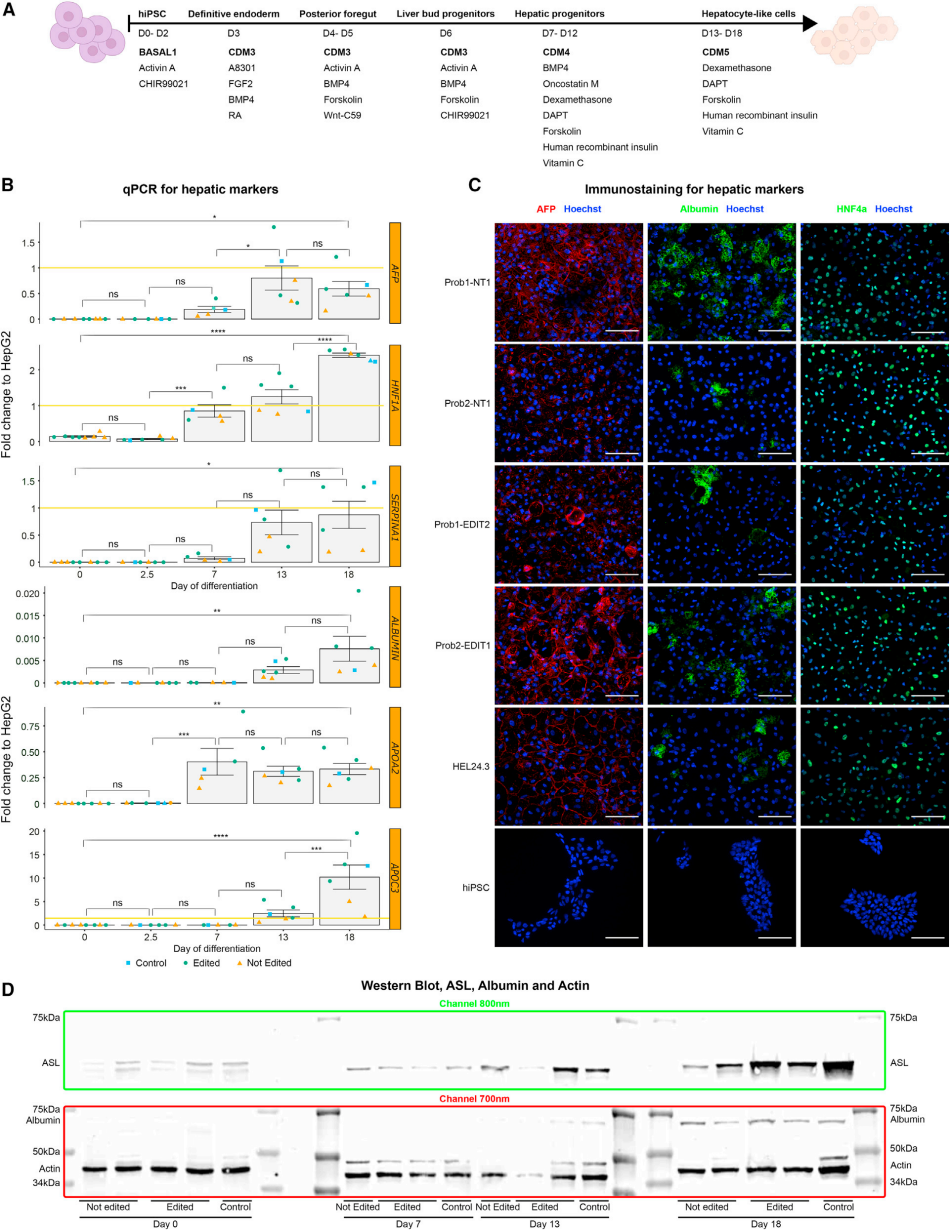
hiPSC differentiation into hepatocyte-like cells (A) Timeline for the 18-day differentiation protocol. The basic media employed for each stage is depicted in bold letters (BASAL1, CDM3, CDM4, and CDM5). The supplements incorporated in each stage are listed below the basic media. (B) mRNA levels of essential hepatocyte marker genes. Representative mRNA samples from different individuals, differentiation batches, stages (day 0, 2.5, 7, 13, and 18), and with different genotypes (control = HEL24.3, edited, and not edited) were analyzed by qPCR. The mRNA levels are expressed in fold change and normalized to the HepG2 commercial hepatocarcinoma cell line (illustrated with a yellow line at the fold change 1 on the y axis when the scale allows it). Each point represents an independent hiPSC line, which we consider a biological replicate. Data are represented as the mean ± SEM. Statistical significance based on Tukey test; p > 0.05 (ns, not significant), p < 0.05 (^∗^), p < 0.01 (^∗∗^), p < 0.001 (^∗∗∗^), p < 0.0001 (^∗∗∗∗^). (C) Representative immunocytochemistry images of day 18 hepatocyte-like cells. Hoechst is depicted in blue, AFP in red, HNF4α, and albumin in green. All the images were acquired and processed with the same settings to allow comparison. The white bar represents 100 μm. (D) Western blot for ASL, albumin, and actin. Representative protein samples from different individuals, stages (day 0, 2.5, 7, 13, and 18), and with different genotypes (control = HEL24.3, edited, and not edited) were imaged at the same time and using fluorescent antibodies for western blot.

To model ASLD *in vitro*, we differentiated the hiPSC lines toward hepatocyte-like cells, a cell type with an active urea cycle, following a previously described protocol,^31^ with some modifications (Figure 1A). On days 0, 2.5, 7, 13, and 18, we assessed the expression of different hepatic markers through qPCR (Figure 1B). The mRNA levels of the hepatocyte markers *AFP*, *HNF1α*, *SERPINA1*, *ALB*, *APOA2*, and *APOC3*^34^^,^^35^^,^^36^ increased significantly along the time points. On day 18, most of the markers presented a similar or higher expression in the hiPSC-derived hepatocyte-like cells compared to that of the HepG2 hepatocellular carcinoma commercial line. Similarly, the immunocytochemistry imaging indicated robust levels of AFP, HNF4α, and albumin in day 18 hepatocyte-like cells (Figure 1C). Finally, the western blot for samples from day 0 to day 18 corroborated clear albumin accumulation by the end of the protocol and a sharp increase in ASL along the differentiation stages (Figure 1D).

These data show that the genetically edited and non-edited hiPSC lines we generated from individuals homozygous for the *ASL* variant meet high-quality standards for genomic integrity, pluripotency markers, and differentiation potential. Importantly, all the proband-derived hiPSC differentiated equally well to hepatocyte-like cells as the HEL24.3 control hiPSC line.^37^

### ABE-mediated editing of the *ASL* c.1153C>T variant reverts the ASA and citrulline accumulation to control levels

**Figure 2:**
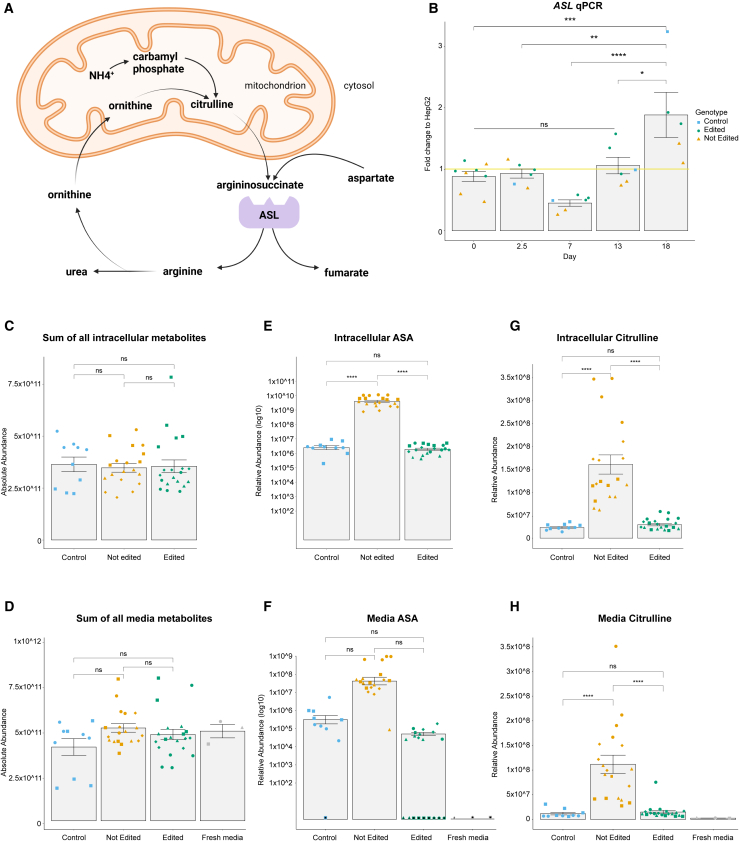
Rescue of the metabolic phenotype after ABE-mediated edition of the ASLD c.1153C>T variant (A) Urea cycle diagram. (B) mRNA levels of *ASL*. Representative mRNA samples from different individuals, differentiation batches, stages (day 0, 7, 13, and 18), and with different genotypes (control = HEL24.3, edited, and not edited) were analyzed by qPCR. The mRNA levels are expressed in fold change and normalized to the HepG2 commercial hepatocarcinoma cell line (illustrated with a yellow line at the fold change 1 on the y axis). Each point represents an independent hiPSC line, which we consider a biological replicate. Data are represented as the mean ± SEM. Statistical significance based on Tukey test; p > 0.05 (ns, not significant), p < 0.05 (^∗^), p < 0.01 (^∗∗^), p < 0.001 (^∗∗∗^), p < 0.0001 (^∗∗∗∗^). (C and D) The sum of the absolute abundance of all the metabolites detected by LC-MS in the cell lysate (C) and the media (D). Each shape represents independent differentiation batches (circle, square, diamond, triangle). We employed day-18 hiPSC-derived hepatocyte-like cells from two different individuals. We analyzed two independently edited hiPSC lines per proband (four biological replicates), two not edited independent hiPSC lines per proband (four biological replicates), and HEL24.3 as a control (two biological replicates). We processed five technical replicates of each sample in the LC-MS. The sum of the absolute abundance of all the metabolites in each sample was employed as a normalization to calculate the relative abundance of individual metabolites. Data are represented as the mean ± SEM. Statistical significance based on Tukey test; p > 0.05 (ns, not significant), p < 0.05 (^∗^), p < 0.01 (^∗∗^), p < 0.001 (^∗∗∗^), p < 0.0001 (^∗∗∗∗^). (E–H) Relative abundance of intracellular and media ASA (E and F) and citrulline (G and H) detected by LC-MS in the same samples described in the previous graph. Each shape represents independent differentiation batches (circle, square, diamond, triangle). As expected, in some of the control and edited samples, the ASA levels were below the detection limit of the mass spectrometer. We did not consider these values for the graph bar or the statistical analysis, but we illustrated these cases with a shape containing a black center. Relative abundance is the absolute abundance value normalized to the sum of all metabolites. Data are represented as the mean ± SEM. Statistical significance based on Tukey test; p > 0.05 (ns, not significant), p < 0.05 (^∗^), p < 0.01 (^∗∗^), p < 0.001 (^∗∗∗^), p < 0.0001 (^∗∗∗∗^).

Prompted by the successful generation of cell models, we tested for the functional rescue. ASL catalyzes the breakdown of ASA into arginine and fumarate, an essential step in the urea cycle (Figure 2A). Elevated ASA and citrulline in plasma are the primary markers used to diagnose ASLD, followed by genetic testing. We confirmed by western blot (Figure 1D) and qPCR (Figure 2B) that ASL accumulation and *ASL* expression both increase along the hepatocyte differentiation protocol. We then differentiated hepatocyte-like cells from a HEL24.3 control hiPSC line, and two edited and two non-edited independent hiPSC lines per individual. To assess the functionality of the urea cycle in the *ASL*-edited and non-edited hepatocyte-like cells, we performed metabolomics analysis of cell-lysate and media samples using LC-MS metabolomics. The sum of the absolute area of all detected metabolites in each sample did not show significant differences between the different cell lines (Figures 2C and 2D), which suggests that our protocol is robust and does not generate artificial differences along the differentiation and sampling. The edited hepatocyte-like cells (*ASL* c.1153 C/C) showed ASA and citrulline levels similar to the control while the non-edited cells (*ASL* c.1153 T/T) presented significantly higher levels of ASA in the cell-lysate and citrulline in the cell-lysate and media (Figures 2E–2H). Together, this demonstrates the relevance of these hiPSC-derived hepatocyte-like cells for disease modeling of ASLD. Importantly, in the edited hepatocyte-like cells (*ASL* c.1153 C/C) ASA and citrulline levels were restored and indistinguishable compared to the control cell line. As our culture media was supplemented with arginine, we did not see major changes in this metabolite or the ones downstream in the urea cycle (Figure S3; Table S4).

Taken together, these results show that, in hepatocyte-like cells, editing the variant *ASL* c.1153C>T restores the urea cycle, preventing the accumulation of ASA and citrulline, metabolites upstream of the affected enzyme. This is an important observation that supports base editing as an effective curative therapy for ASLD.

### LNP-mediated *ASL* editing and metabolic phenotype normalization in ASLD fibroblasts

**Figure 3:**
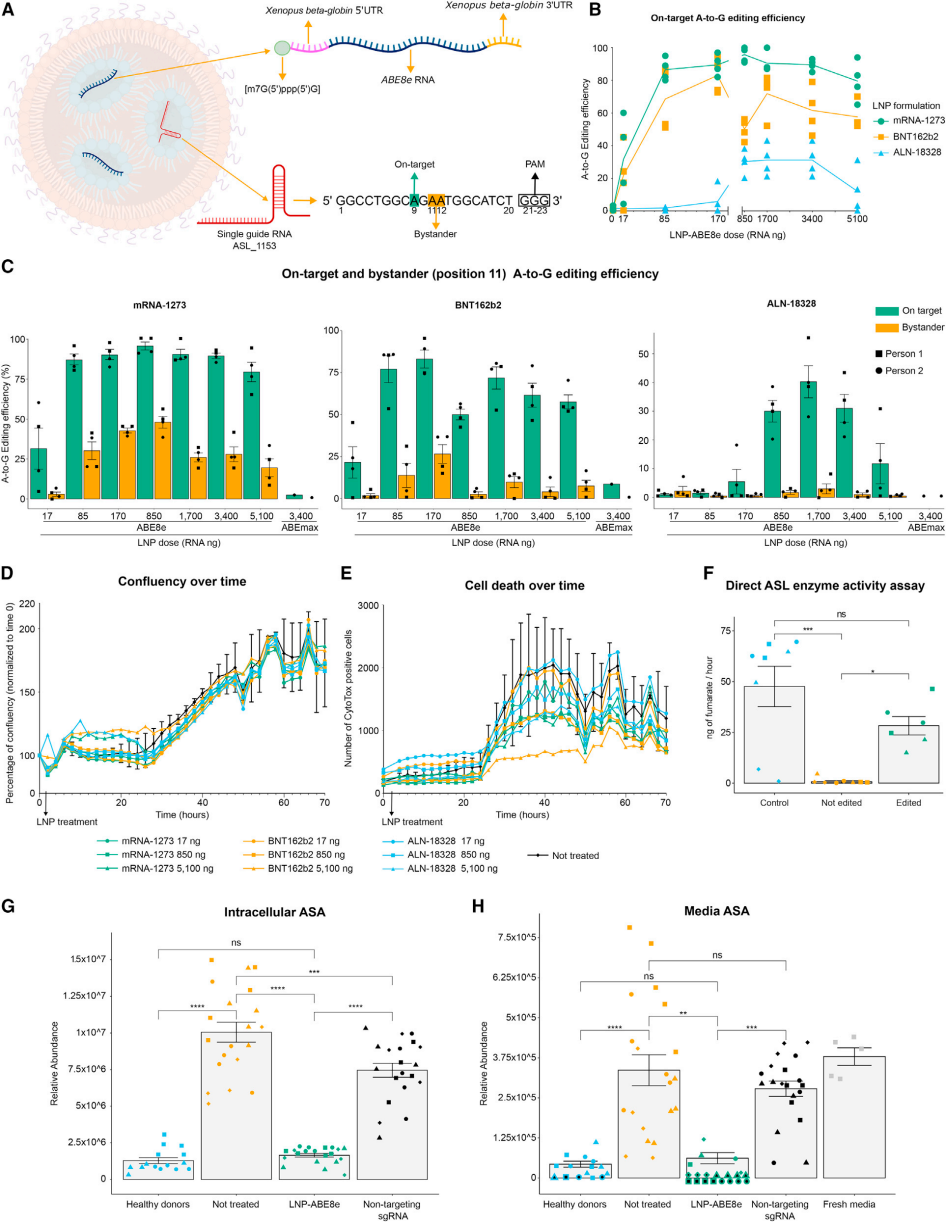
Editing efficiency, toxicity profile, and rescue of the metabolic phenotype in primary fibroblasts after lipid nanoparticles ABE8e treatment (A) Diagram of the lipid nanoparticle contents: an sgRNA targeting the *ASL* c.1153C>T variant plus an RNA cassette for ABE8e expression. The spacer section of the sgRNA, which targets the DNA, is written from base 1 to base 20. The PAM, not included in the sgRNA oligo, corresponds to bases 21–23 (GGG). (B) On target A-to-G editing efficiency. We employed primary fibroblasts from two different individuals. We independently treated these fibroblasts in duplicates (four biological replicates) with eight different doses (0–5,100 ng RNA) of three types of lipid nanoparticle ABE8e (mRNA-1273, BNT162b2, and ALN-18328). One week after the treatment, we estimated the on-target A-to-G editing efficiency by analyzing the Sanger sequence data through EditR.^42^ The solid lines represent the mean of each treatment, and each data point is individually represented. (C) Sequencing data from the experiment in point B is shown here in detail, considering the on-target (green) and bystander (orange) A-to-G editing efficiency. We found bystander editing just in the adenine position 11 but not in position 12. Data are represented as the mean ± SEM. A mid-high dose of ABEmax with no replicates was added as a comparison showing the editing efficiency of the previous generation of ABE. (D and E) We independently treated primary fibroblasts from two different individuals in duplicates (four biological replicates) with three different doses (17, 850, and 5,100 ng RNA) of three types of lipid nanoparticle ABE8e (mRNA-1273, BNT162b2, and ALN-18328). We followed the fibroblast populations for 70 h, taking pictures in the Incucyte every 2 h. The lipid nanoparticle ABE8e treatment was applied at the 2 h time point. We assessed the confluency (D) and the number of dead cells (E) estimated by the CytoTox dye. The solid lines represent the mean of each treatment. For simplicity, the error bars (SD) are shown only for the non-treated samples (in black). For more detailed data, please check Figure S5. (F) Direct ASL enzyme activity in fibroblast cell lysates. We employed cell lysates from two fibroblast populations derived from each of the two healthy donors (control, four biological replicates), three ASLD fibroblast populations independently treated with ABE8e mRNA-1273 LNPs targeting the *ASL* variant (edited, three biological replicates), and two not-treated fibroblast populations per each of the two individuals (not edited, four biological replicates). Each sample was processed in two technical replicates. Within each treatment, the data points illustrated with the same shape represent a technical replicate of the enzyme activity assay using the same fibroblast lysate. For the edited group, the triangle and the circle correspond to a lipid nanoparticle dose of 85 ng of RNA, whereas the square corresponds to a dose of 1,700 ng of RNA. Data are represented as the mean ± SEM. Statistical significance based on Tukey test; p > 0.05 (ns, not significant), p < 0.05 (^∗^), p < 0.01 (^∗∗^), p < 0.001 (^∗∗∗^), p < 0.0001 (^∗∗∗∗^). (G and H) Relative abundance of ASA in the cell lysate (G) and the media (H) detected by LC-MS. We independently treated primary fibroblasts from two different individuals in duplicates (four biological replicates) with 85 ng RNA of the mRNA-1273 lipid nanoparticle ABE8e plus the variant-targeting sgRNA (LNP-ABE8e), or with vehicle (not treated), or with mRNA-1273 lipid nanoparticle ABE8e containing the sgRNA site_16 targeting an unrelated locus (non-targeting sgRNA). As a control, we used fibroblasts coming from three healthy donors of different genders and ages (healthy donors, three biological replicates). Two weeks after the treatment, we analyzed the metabolite content of each condition, processing five technical replicates of each sample in the LC-MS. As expected, in some of the control and edited samples the ASA levels were below the detection limit of the mass spectrometer. We did not consider these values for the graph bar or the statistical analysis, but we illustrated these cases with a shape containing a black center. Relative abundance is the absolute abundance value normalized to the sum of all metabolites. Data are represented as the mean ± SEM. Statistical significance based on Tukey test; p > 0.05 (ns, not significant), p < 0.05 (^∗^), p < 0.01 (^∗∗^), p < 0.001 (^∗∗∗^), p < 0.0001 (^∗∗∗∗^).

After confirming that the ABE allows us to efficiently edit the *ASL* c.1153C>T variant thus restoring the ASA and citrulline levels, we sought to design a system compatible with *in vivo* delivery. This is a key step in any gene-editing therapy, as the CRISPR tools need to reach the target organ, in this case, the liver, and edit the genome of a sufficient number of cells. Electroporation is not an option for *in vivo* delivery, therefore, we decided to develop a proof-of-concept LNPs approach. As the ABEmax was not efficient when delivered by LNPs (Figure 3C), we employed a more efficient version of the ABE, the ABE8e, which presents a more processive deaminase component.^26^ We designed an mRNA cassette containing the ABE8e coding sequence flanked by the untranslated 5′ and 3′UTRs from the *Xenopus beta-globin* gene.^38^ These modifications plus a 5’ m7G(5′)ppp(5′)G and a synthetic polyA tail, increase mRNA stability, translation efficiency, and lower immunogenicity.^39^^,^^40^^,^^41^ LNPs represent an optimal delivery tool due to their high efficiency, safety profile, and industrial scalability potential demonstrated in the COVID-19 mRNA vaccine production and administration. Commercial FDA-approved LNP formulations consist of different combinations of lipids, and we benchmarked three different ones: mRNA-1273 (Moderna), BNT162b2 (Pfizer-BioNTech), and ALN-18328 (Onpattro/patisiran),^27^^,^^28^^,^^29^ to determine their efficiency at editing the variant *ASL* c.1153C>T. We produced these LNPs to encapsulate and deliver our optimized ABE8e mRNA and the sgRNA ASL_1153 targeting the variant (Figure 3A). Our first goal was to achieve a dose-dependent A-to-G editing efficiency and to find the lowest dose with the highest effect. We treated primary fibroblasts from the two individuals homozygous for the *ASL* variant in duplicates using different doses, defined as the final amount of RNA computing the mRNA and the sgRNA mass. The on-target A-to-G editing efficiency greatly increased from the LNP ABE8e doses of 17–85 ng but remained at a similar level for higher doses, even after 5,100 ng (Figure 3B). All three formulations exhibited a dose-dependent pattern, but mRNA-1273 and BNT162b2 performed considerably better than ALN-18328, with editing efficiencies surpassing 90% in bulk fibroblast populations (Figure 3C).

The ABE preferentially targets bases 4–8 within the locus targeted by the sgRNA,^43^ which represents the editing window (Figure 3A). The ASLD variant that we aimed to edit is located in position 9 of the protospacer on the sgRNA ASL_1153, but this locus presents other adenines (called bystanders) in positions 11 and 12. Using Sanger sequencing, we did not find any editing above the 5% detection threshold for adenine 12 in any of the samples. However, we did detect A-to-G editing for the bystander adenine 11 (Figure 3C), but in all cases, the on-target was higher than the bystander editing. The unwanted change of adenine 11 into guanine resulted in an amino acid change from the uncharged, nonpolar, and hydrophobic phenylalanine to an uncharged, polar, and hydrophilic serine. This amino acid change may affect the ASL structure or activity, as it is located near the active site of the enzyme.^16^ The mRNA-1273 85 ng was the lowest dose that resulted in a high on-target A-to-G editing efficiency (86.8% ± 2.7 mean ± SEM) with a relatively low bystander effect (30.5% ± 9.7 mean ± SEM), and no off-target effects (Table S1).

To explore alternatives to lower the bystander effect, we tested a second sgRNA (ASL_1153_II) consisting of a 20 bases protospacer that is shifted one base toward the 5′ of the DNA strand (Figure S4). The protospacer adjacent motif (PAM) for this new sgRNA is TGG, and the target *ASL* variant lies at position 10 instead of position 9 as described for the sgRNA ASL_1153. By shifting the protospacer and, therefore, the editing window, the main bystander (A11 on the previous sgRNA) is now in position 12, which would lower the A-to-G editing efficiency for this adenine. Testing this alternative sgRNA in primary fibroblasts yielded no bystanders, which is positive for a clinical setting but also a much lower on-target editing efficiency (Figure S4). Considering the superior on-target editing efficiency of the initial sgRNA ASL_1153, we opted to continue with this guide for further analysis.

To better quantify the allele frequency and diversity after gene editing, we resampled the primary fibroblast populations previously treated with three different doses (17, 85, and 170 ng RNA) of the LNP formulations mRNA-1273 and BNT162b2 carrying the ABE8e mRNA cassette and the sgRNA ASL_1153. We analyzed the samples by amplicon long-read sequencing technology from Oxford Nanopore Technologies (ONT) and estimated the allele frequency using the software CRISPResso2.^44^ We then computed the allele frequency into three categories: precisely edited alleles, those that contained any bystanders or indels, and those that remained unchanged (Figure S5). These data indicate that the editing efficiency, in general, was high even at low LNP doses such as 85 ng and that the frequency of bystanders increases rapidly at higher concentrations such as 170 ng, which is particularly clear in the mRNA-1273 ABE8e treatment.

To assess the toxicity of the different LNP ABE8e formulations, we treated primary fibroblasts from the two individuals homozygous for the *ASL* variant in duplicates using a null (not treated), a minimum (17 ng), a medium (850 ng), and a high LNP ABE8e dose (5,100 ng). During the 70 h follow up, we monitored the number of dead cells and the confluency, which did not significantly differ between any of the treatments and the non-treated samples (Figures 3D, 3E, and S5), indicating the safety of the treatment.

To analyze in detail the off-target activity of the LNP-delivered ABE8e, we selected for each formulation (mRNA-1273 and BNT162b2) the two fibroblast populations with the highest on-target editing efficiency. We then generated a list of 11 putative off-target sites using three different prediction software, Benchling, CRISPOR, and IDT (Table S2); amplified through PCR each locus; and sequenced the DNA samples through ONT. We aligned the raw reads to the reference genome using CRISPResso2 and compared the profile with a non-treated sample. We did not see any difference in the insertion and deletion quantification, but we could detect that the off-target 8 presented substantial A>G conversion (Figure S6). This site lies on the *ASL* pseudogene *ASLP1* (RefSeq: NG_002637). The 4,791 bp long *ASLP1* showed an average sequence identity of 85% to *ASL* after alignment with the National Center for Biotechnology Information (NCBI) Nucleotide BLAST tool, and it correlates with an immunoglobulin-lambda-like mRNA.^45^

To assess the effect of the gene editing, we measured the ASL enzyme activity in a direct assay from edited, not edited, and control fibroblast cell lysates. Importantly, the treatment with the LNP ABE8e targeting the *ASL* variant significantly increased the enzyme activity in comparison to the non-treated populations, restoring the null activity to around 59% of the healthy donor levels (Figure 3F). This indicated the high potential of LNP-delivered ABE to restore ASL enzyme activity.

Finally, we analyzed the metabolic phenotype following the mRNA-1273 85 ng LNP ABE8e treatment by assessing the intracellular and media levels of ASA and other urea cycle metabolites. We compared fibroblasts derived from three healthy donors against two independently treated populations (edited), two not treated, and two treated with an sgRNA targeting a non-related gene (non-targeting sgRNA) per individual. As expected, the non-targeting-sgRNA and the non-treated populations showed high ASA levels. Importantly, we observed a significant drop in the ASA levels in the cell lysate and the media of the fibroblasts treated with lipid nanoparticle ABE8e targeting the *ASL* variant, reaching the levels of the healthy donors (Figures 3G and 3H).

These results highlight the potential of the LNP-delivered ABE as a method to edit the Finnish founder variant *ASL* c.1153C>T in primary cells *in vitro*. The data show a highly efficient and dose-dependent editing of the variant and a strong phenotypic restoration in the ASA levels and an increase in ASL activity, which suggests recovery of the urea cycle.

## Discussion

ASLD presents with symptoms along a continuum of severity, often fatal in the neonatal period. The individuals homozygous for the *ASL* variant c.1153C>T are on the severe end of the disease spectrum.^6^^,^^46^ Newborn screening enables early diagnosis and improves the initial management of ammonia levels, but in the long-term, the frequency of hyperammonemia episodes does not decrease, even in people with an early diagnosis,^47^ and neurological complications still develop.^46^ Given the possibility of early diagnosis but with yet unsatisfying treatment outcomes, new therapies are highly needed.^46^ We show here that base editors can effectively edit *ASL* c.1153C>T variant and enzyme activity, presenting a proof-of-principle of a gene editing approach for treating ASLD.

Rapidly developing gene editing techniques enable the correction of genetic diseases, which expands the therapeutic options and broadens the field of clinical genetics. Certain medical conditions are especially promising for therapeutic interventions; for example, symptoms and signs caused by a systemic toxic metabolite are a more straightforward target for treatment than those caused by a structural defect in cells. Before any therapeutic development, the functional consequences of the disease variant and the effects of the gene editing require careful evaluation.

Our study reports a proband-derived hiPSC-based model for the urea cycle dysfunction in ASLD. Our hepatocyte-like cells display typical hepatocyte markers and show an upregulation of *ASL* expression, confirming their hepatic identity and suitability as an ASLD model. The level of *ASL* mRNA expression or protein accumulation did not differ between edited and non-edited primary cells, consistent with previous findings of the c.1153C>T variant.^15^ The differentiation protocol seemed to yield a rather heterogeneous cell population, with an uneven distribution of albumin and HNF4α in individual cells. Still, the *in vitro* model successfully replicated key metabolic features observed in ASLD, including ASA accumulation and increased citrulline levels.

In healthy individuals, ASA is not detected in plasma or urine, as intracellularly produced ASA is immediately cleaved to arginine and fumarate. The individuals in this study presented a high concentration of ASA in plasma at diagnosis, and they continued to show elevated levels during the follow-up despite the protein-restricted diet treatment. They also had increased plasma concentration of citrulline, the upstream metabolite in the urea cycle. We replicated these findings in hepatocyte-like cells, showing the abundance of ASA and citrulline in the cell lysates as well as in the cell culture media from c.1153T/T primary cells. Furthermore, we found ASA and citrulline levels similar to those of healthy controls in the isogenic c.1153C/C primary cells corrected by base editing, proving restoration of urea cycle function.

To generate a CRISPR gene editing approach with potential for *in vivo* applications, we encapsulated the ABE into LNPs. We compared three FDA-approved LNP formulations^27^^,^^28^^,^^29^ in fibroblasts aiming to edit the *ASL* c.1153C>T variant. This method yielded a high editing efficiency (up to 100%) in a dose-dependent manner. In addition to on-target editing, we also found, with a lower frequency, an unwanted but expected bystander editing change. The bystander edit may negatively affect the ASL structure or function. Nevertheless, the treatment in fibroblasts was effective: it reduced ASA levels to those of the healthy donors and restored the ASL enzyme activity. To evaluate the potential of this approach as a possible therapy, the optimal level of gene editing should be considered. Heterozygous *ASL* c.1153C>T individuals are asymptomatic, suggesting that not all cells or alleles need to be edited. Restoring the ASL activity to 10% of the normal range may protect from severe disease while surpassing the 25% could prevent cognitive impairment.^15^ Interestingly, in *ASL* hypomorphic mouse model,^13^ a high dose of the liver-targeting adeno-associated virus serotype 8 (AAV8) expressing human codon-optimized *ASL,* recovered up to 25% of the enzyme’s activity, restoring the plasma ASA and citrulline levels, and increasing survival and weight gain.^48^ Achieving similar enzyme activities through gene editing in ASL-deficient individuals could restore a healthy metabolic profile. In our study, the ABE lipid nanoparticle treatment was not cytotoxic and rescued an average of 59% of the ASL enzyme activity in the primary fibroblasts, while the non-treated lines showed very low ASL activity, consistent with the previous reports of this variant.^15^^,^^17^^,^^18^ Additionally, the ASL activity levels in control cell lines in our experiment were similar to those previously reported.^17^ Moreover, the ASA concentration significantly decreased in cell lysates and culture media of edited fibroblast indicating that genetically edited cells could clear ASA from their cytoplasm and their environment.

In clinical trials, LNPs for gene editing have successfully targeted the liver.^49^^,^^50^ Our results suggest that LNPs are a highly promising tool for the editing of the *ASL* variant in the liver. Such a treatment would restore the urea cycle, protect from hyperammonemia, and, importantly, clear ASA from circulation, preventing the need for liver transplantation, which represents an effective but highly invasive treatment to reduce ASA blood levels.^51^ However, in contrast to transplantation, a liver-targeted gene editing therapy would not require lifelong immunosuppression. In addition to the neurotoxic effects of ammonia, ASA has been suggested to be toxic,^2^^,^^8^ as demonstrated by the correlation of higher ASA levels to higher transaminases.^3^^,^^52^ This, in turn, indicates a risk for liver fibrosis, a known complication of ASLD.^53^ ASA is also detected in high concentrations in the cerebrospinal fluid of ASL-deficient individuals^54^ and can be metabolized to neurotoxic guanidinoacetate.^2^^,^^11^^,^^12^ Therefore, editing this variant with the ABE in the central nervous system could have a significant impact on preventing neurological complications, as only editing the variant in hepatocytes may not be enough to tackle the neuronal presentation of the disease. This is technically possible, and a mouse study has reported successful gene editing in the brain.^55^ Furthermore, the ASL enzyme, in addition to its role in the urea cycle, produces and channels arginine for NO production, contributing to blood pressure regulation.^2^ Editing the ASLD variant in vascular smooth muscle cells or endothelial cells might be technically possible, as these cell types have previously been edited with different CRISPR systems.^56^^,^^57^^,^^58^^,^^59^

Gene therapy options are rapidly evolving and under active investigation for urea cycle disorders.^60^ Our gene editing strategy offers advantages over gene transfer by viral vectors. LNPs present lower immunogenicity compared to viral vectors, circumventing the risk of existing or triggered anti-viral antibodies, and possibly allowing for subsequent applications of the treatment. The edited gene continues to be expressed under endogenous promoters and physiological regulation, avoiding artificial overexpression of AAV or lentivirus. For *ASL*, this could be important, as its expression in the kidney, lung, and spleen increases after inflammatory stimuli in rats.^61^^,^^62^

Pre-existing adaptive immune responses to Cas9 protein, the main component of the ABE, have been reported in the general population,^63^^,^^64^^,^^65^^,^^66^ which could hinder the efficient gene editing of the targeted organ *in vivo*. In addition, mouse studies have reported inflammatory reactions^67^ and a cytotoxic CD8^+^ T response^68^ associated with Cas9 expression after AAV delivery. From a translational perspective, it is essential to monitor the immune response against Cas9 and the delivery tool of choice before and after the treatment, as it is routinely done in gene-editing clinical trials. Another risk to consider is the possibility of off-target effects, unwanted modifications in regions of the genome not targeted by the sgRNA. Our off-target analysis, in agreement with the literature,^26^^,^^69^ showed that the ABE8e induces A>G conversions in some loci with a sequence similar to that of the sgRNA. Testing the safety profile of this base editing tool in a preclinical study would require genome-wide off-target analysis and an oncogene screening to understand the risks associated with this therapy. An alternative to improve the specificity could be the ABE8e V106W^26^ or SuperFi^69^ alternatives, with reduced off-target and on-target activity.

Employing RNA instead of DNA improves the safety of gene editing strategies by ensuring the quick degradation of the vector, transient expression of the encoded Cas9 or ABE protein, and reduced risk of vector integration. GMP lipid nanoparticle RNA vaccines demonstrate scalable artificial synthesis without bioreactors or viral vectors, as witnessed during the COVID-19 pandemic. Our lipid nanoparticle delivery approach for the ABE is a proof-of-principle for successful gene editing therapy in urea cycle disorders and paves the way to systemic trials.

In summary, this study provides a significant advancement in modeling ASLD and proposes a potential therapeutic approach using lipid nanoparticle delivered CRISPR base editor to edit the *ASL* c.1153C>T variant. This technology’s advantages over viral vectors, coupled with its reported ability to target the liver, are auspicious for addressing the metabolic systemic phenotype of ASLD and improving the health and prognosis of people suffering from this disease.

## Data and code availability

The published article includes all datasets generated or analyzed during this study.

## Supplemental information

Document S1. Figures S1–S6, Tables S1–S3, and supplemental methods

Table S4. Metabolomic data

Document S2. Article plus supplemental information
