# Supplementary material for: Genetic and functional correction of argininosuccinate lyase deficiency using CRISPR adenine base editors
Source: Am J Hum Genet. 2024 Apr 4;111(4):714–28. doi: 10.1016/j.ajhg.2024.03.004 (PMC11023919; doi:10.1016/j.ajhg.2024.03.004)
Supplement: Document S1. Figures S1–S6, Tables S1–S3, and supplemental methods [file mmc1.pdf]

The American Journal of Human Genetics, Volume *111*

Supplemental information

Genetic and functional correction of argininosuccinate lyase deficiency using CRISPR adenine base editors

Sami Jalil, Timo Keskinen, Juhana Juutila, Rocio Sartori Maldonado, Liliya Euro, Anu Suomalainen, Risto Lapatto, Emilia Kuuluvainen, Ville Hietakangas, Timo Otonkoski, Mervi E. Hyvönen, and Kirmo Wartiovaara

Supplemental Figures and Legends. Figure S1

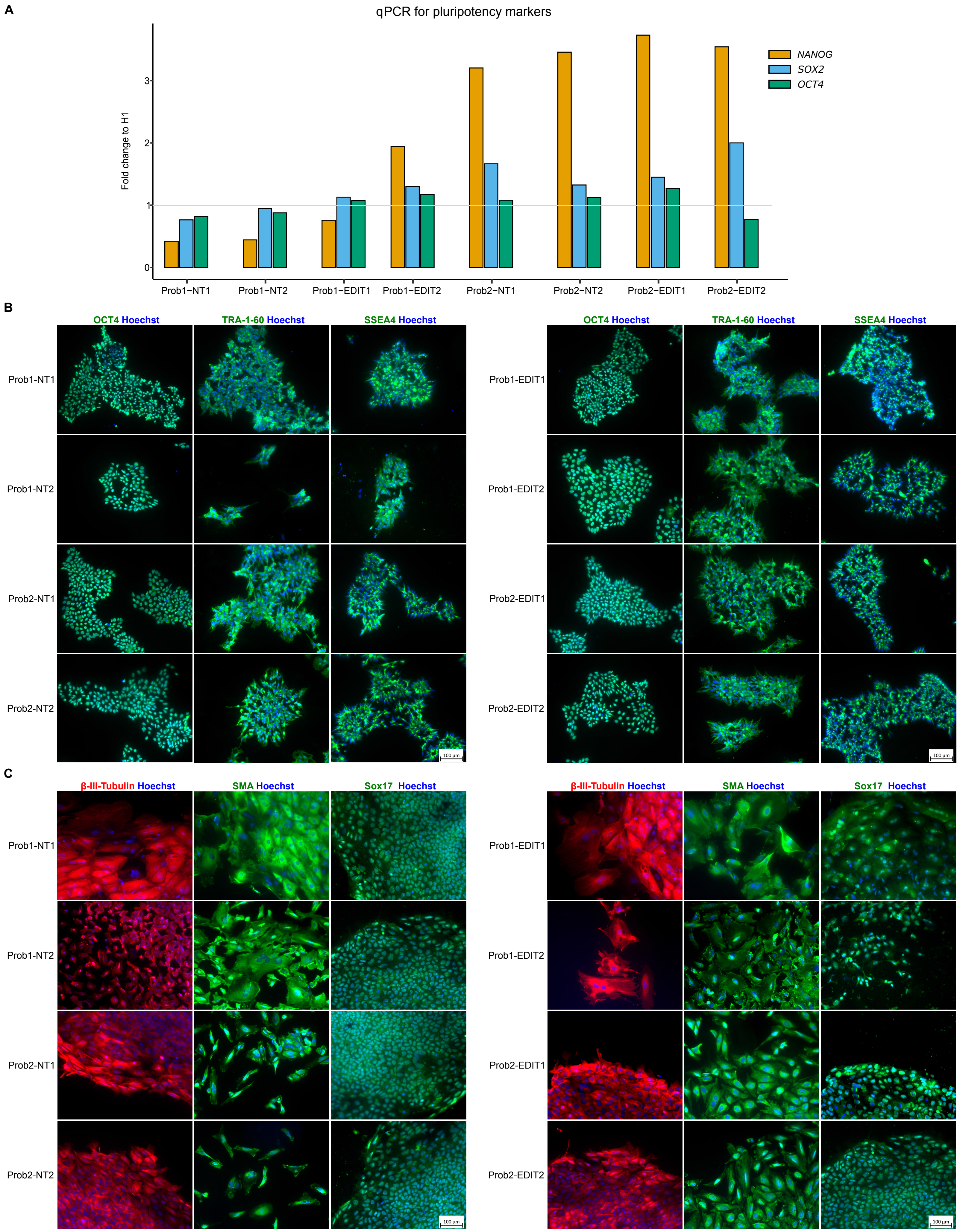

Figure S1: Assessment of the hiPSC lines pluripotency.
(A) We tested the mRNA levels of three essential pluripotency markers (*NANOG*, *SOX2*, and *OCT4*) in two edited hiPSC lines (*ASL* c.1153 C/C) and two non-edited hiPSC lines (*ASL* c.1153 T/T) per proband. These hiPSCs were later employed for hepatocyte differentiation. **(B)** Immunofluorescence staining of the same hiPSC lines illustrating a normal morphology and expression of the pluripotency markers OCT4, TRA-1-60, and SSEA4. Hoechst, in blue, is a nuclear marker. **(C)** Immunofluorescence staining of embryoid bodies derived from the same hiPSC lines. β-III-Tubulin (ectoderm), smooth muscle actin (SMA) (mesoderm), and Sox17 (endoderm). Hoechst, in blue, is a nuclear marker.

Figure S2

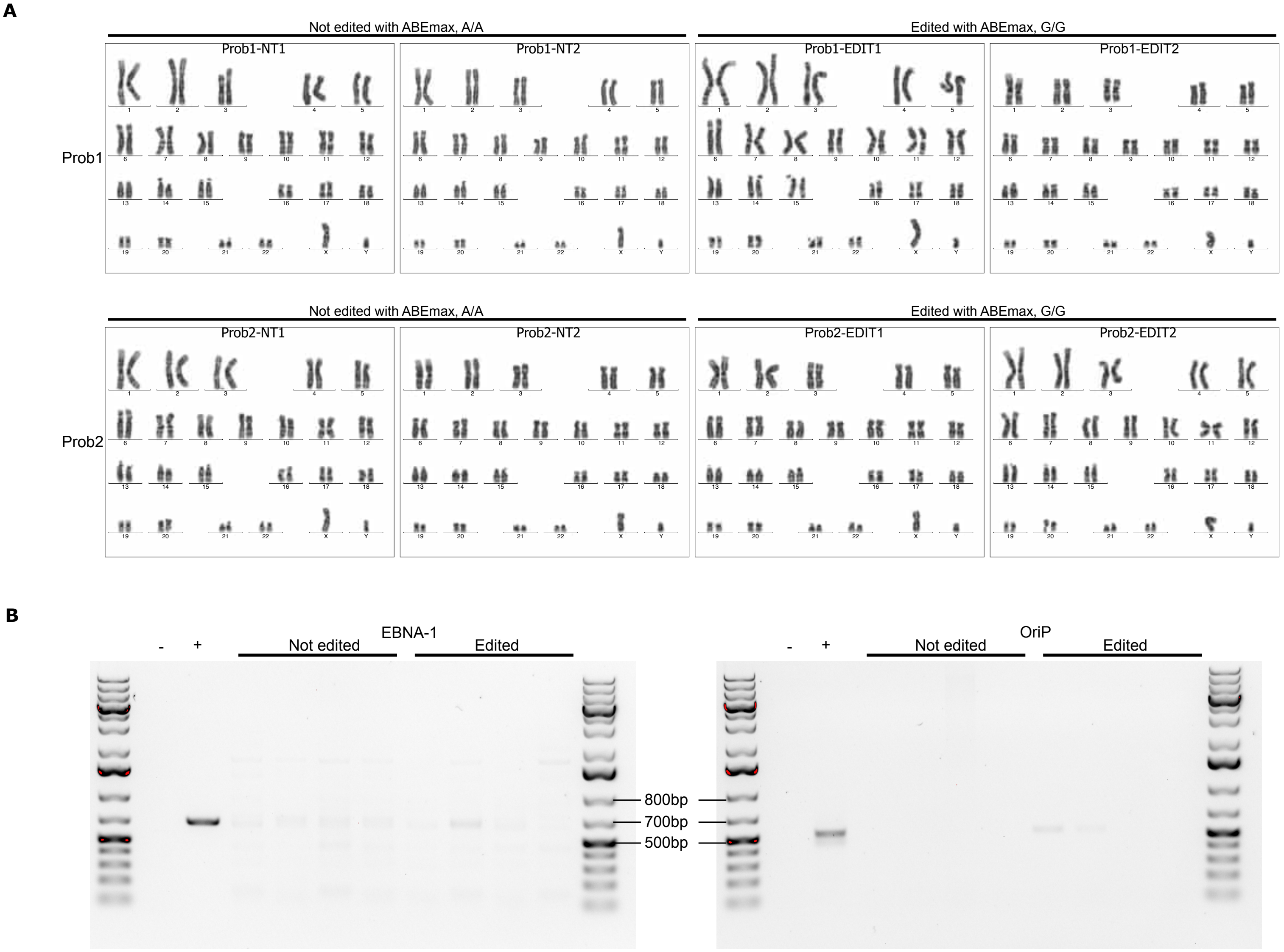

Figure S2: Genomic integrity of the edited and not edited hiPSC monoclonal lines.
(A) Normal karyotype of four hiPSC monoclonal lines reprogrammed from proband fibroblasts in the absence of the ABEmax construct (not edited) and four hiPSC monoclonal lines reprogrammed and simultaneously treated with the ABEmax construct (edited). **(B)** PCR to detect the episomal reprogramming vectors retention in four hiPSC monoclonal lines reprogrammed from proband fibroblasts in the absence of the ABEmax construct (not edited) and four hiPSC monoclonal lines reprogrammed and simultaneously treated with the ABEmax construct (edited). Two different plasmid regions are targeted in these PCRs: EBNA-1 and OriP. Water control (−), positive control (+).

Figure S3

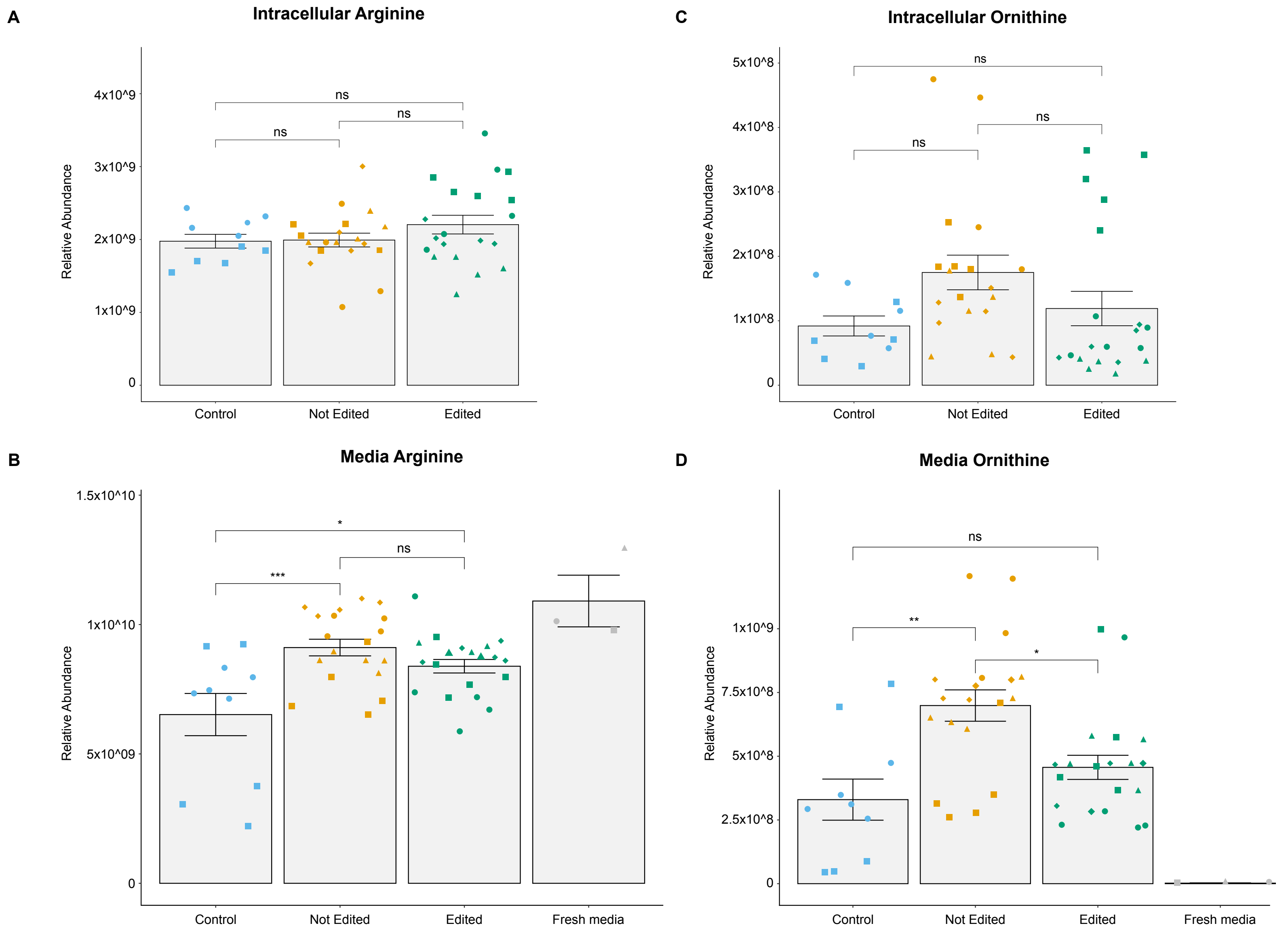

Figure S3: Metabolomic analysis of hiPSC-derived hepatocyte-like cells.
(A-D) Relative abundance of arginine (A-B) and ornithine (C-D) in the cell lysate and the media by LC-MS. Each shape represents independent differentiation batches (circle, square, diamond, triangle). We employed day-18 hiPSC-derived hepatocyte-like cells from two different probands. We analyzed two independently edited hiPSC lines per proband (four biological replicates), two not edited independent hiPSC lines per proband (four biological replicates), and HEL24.3 as a control (two biological replicates). We processed five technical replicates of each sample in the LC-MS. Relative abundance is the absolute abundance value normalized to the sum of all metabolites. Data are represented as the mean ± SEM. Statistical significance based on Tukey test; $p>0.05$ (ns, not significant), $p < 0.05$ (*), $p < 0.01$ (**), $p < 0.001$ (***), $p < 0.0001$ (****).

Figure S4

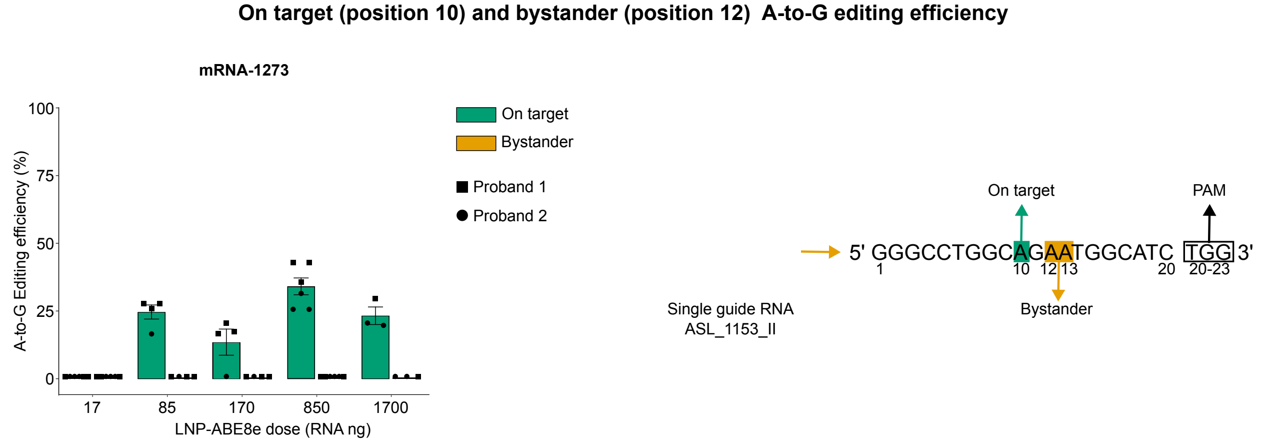

Figure S4: On target and bystander A-to-G editing efficiency using an alternative sgRNA.
On target (green) and bystander (orange) A-to-G editing efficiency. We employed primary fibroblasts from two different individuals. We independently treated these fibroblasts in triplicates (n=6) with five different doses (17 to 170 ng RNA) of the lipid nanoparticle formulation mRNA-1273 for the delivery of ABE8e together with the alternative sg ASL_1153_II. One week after the treatment, we estimated the on-target A-to-G editing efficiency by analyzing the Sanger sequence data through EditR62. Data are represented as the mean ± SEM, each data point is individually represented.

Figure S5

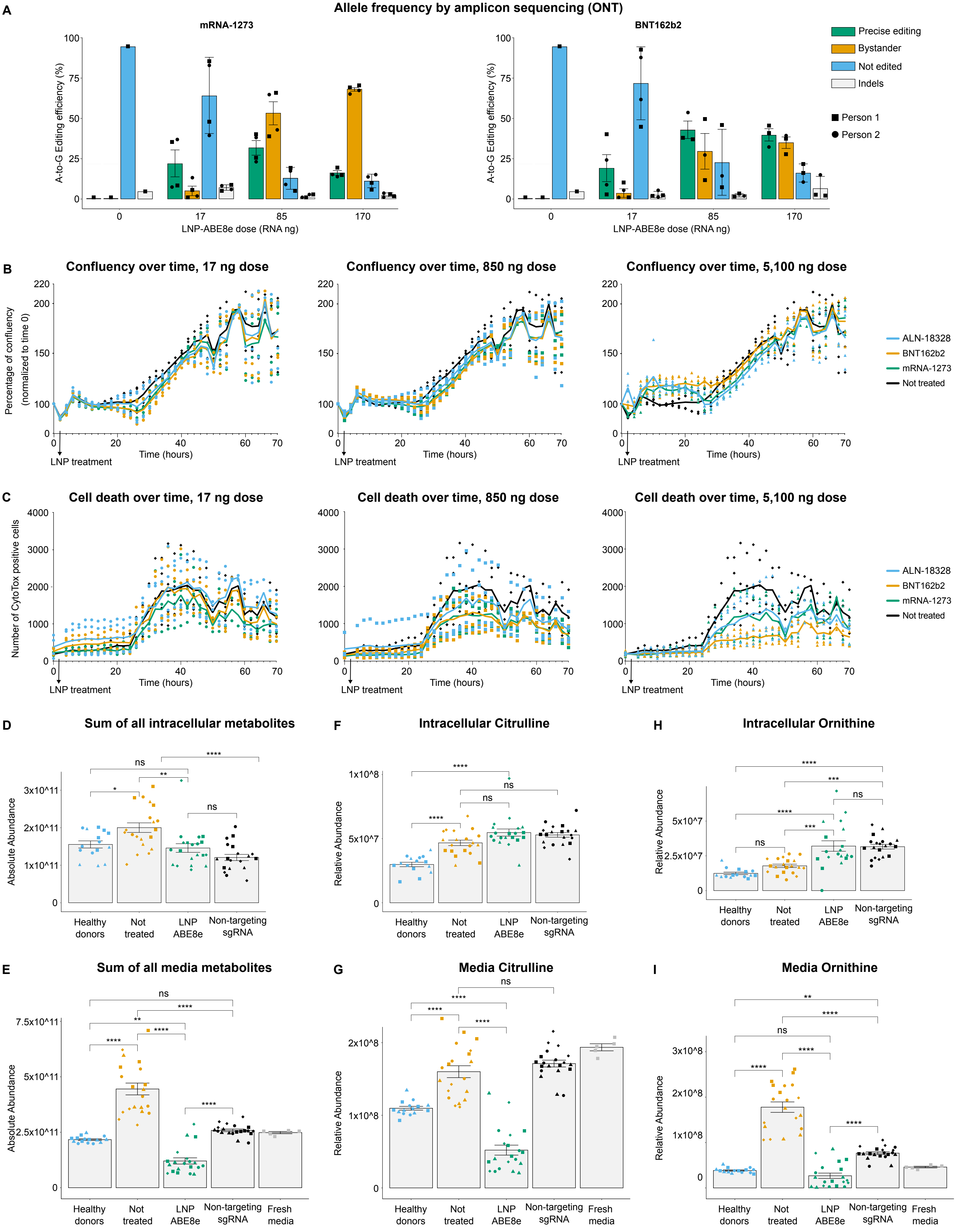
A
Allele frequency by amplicon sequencing (ONT)
mRNA-1273
BNT162b2
A-to-G Editing efficiency (%)
LNP-ABE8e dose (RNA ng)
Precise editing
Bystander
Not edited
Indels
Person 1
Person 2
B
Confluency over time, 17 ng dose
Confluency over time, 850 ng dose
Confluency over time, 5,100 ng dose
Percentage of confluency (normalized to time 0)
Time (hours)
LNP treatment
ALN-18328
BNT162b2
mRNA-1273
Not treated
C
Cell death over time, 17 ng dose
Cell death over time, 850 ng dose
Cell death over time, 5,100 ng dose
Number of CytoTox positive cells
Time (hours)
LNP treatment
D
Sum of all intracellular metabolites
Absolute Abundance
F
Intracellular Citrulline
Relative Abundance
H
Intracellular Ornithine
Relative Abundance
Healthy donors
Not treated
LNP ABE8e
Non-targeting sgRNA
E
Sum of all media metabolites
Absolute Abundance
G
Media Citrulline
Relative Abundance
I
Media Ornithine
Relative Abundance
Healthy donors
Not treated
LNP ABE8e
Non-targeting sgRNA
Fresh media

Figure S5: Detailed toxicity profile, allele frequency by amplicon sequencing (ONT), and metabolomic analysis of proband primary fibroblasts after LNP-ABE8e treatment.
(A) We independently treated primary fibroblasts from two different probands in duplicates (n=4) with three different doses (17, 85, and 170 ng RNA) of two types of lipid nanoparticles (mRNA-1273 and BNT162b2) carrying the ABE8e mRNA cassette and the ASL targeting sgRNA. One week after the treatment, we estimated the allele frequency by amplicon long-read sequencing technology from Oxford Nanopore Technologies (ONT). We analyzed the data using CRISPResso2. We first filtered out those reads with less than 50% identity to the genome of reference and also those with less than 1% representation, which reduces the noise introduced by sequencing errors. We then quantified the reads in groups: "Precise Editing" refers to the alleles containing only the desired A-to-G edit on base 9 of the protospacer; "Bystander" refers to any allele containing one or more undesired A-to-G on the protospacer; "Not Edited" refers to the alleles with the unchanged proband sequence; "Indels" refers to any allele containing an insertion or deletion. Data are represented as the mean ± SEM. **(B)** We independently treated primary fibroblasts from two different probands in duplicates (n=4) with three different doses (17, 850, and 5,100 ng RNA) of three types of lipid nanoparticles ABE8e (mRNA-1273, BNT162b2, and ALN-18328). We followed the fibroblast populations for 70 hrs, taking pictures in the Incucyte® every 2 hrs. The lipid nanoparticle ABE8e treatment was applied at time 2 hrs. The solid lines represent the mean confluency of each treatment, each data point is individually expressed. **(C)** In the same setting as the previous graph, we assessed the number of dead cells, estimated by the CytoTox dye. The solid lines represent the mean number of CytoTox-positive cells for each treatment, each data point is individually expressed. **(D-E)** The sum of the absolute abundance of all the metabolites detected by LC-MS in the cell lysate and the media. Each shape represents independent lipid nanoparticle treatments (circle, square, diamond, triangle). We employed primary fibroblasts from two different probands. We independently treated these fibroblasts in duplicates (four biological replicates) with 85 ng RNA of the mRNA-1273 lipid nanoparticle ABE8e plus the variant-targeting sgRNA (LNP-ABE8e), or with vehicle (not treated), or with mRNA-1273 lipid nanoparticle ABE8e containing the sgRNA Site_16 targeting an unrelated locus (non-targeting sgRNA). As a control, we used fibroblasts coming from three healthy donors of different genders and ages (healthy donors) (three biological replicates). Two weeks after the treatment, we analyzed the metabolite content of each condition in five technical replicates. The sum of the absolute abundance of all the metabolites in each sample was employed as a normalization to calculate the relative abundance of individual metabolites. Data are represented as the mean ± SEM. **(F-I)** Relative abundance of intracellular and media citrulline **(F-G)** and ornithine **(H-I)** measured by LC-MS in the same samples described above. Relative abundance is the absolute abundance value normalized to the sum of all metabolites. Data are represented as the mean ± SEM. Statistical significance based on Tukey test; $p>0.05$ (ns, not shown), $p < 0.05$ (*), $p < 0.01$ (**), $p < 0.001$ (***), $p < 0.0001$ (****).

Figure S6

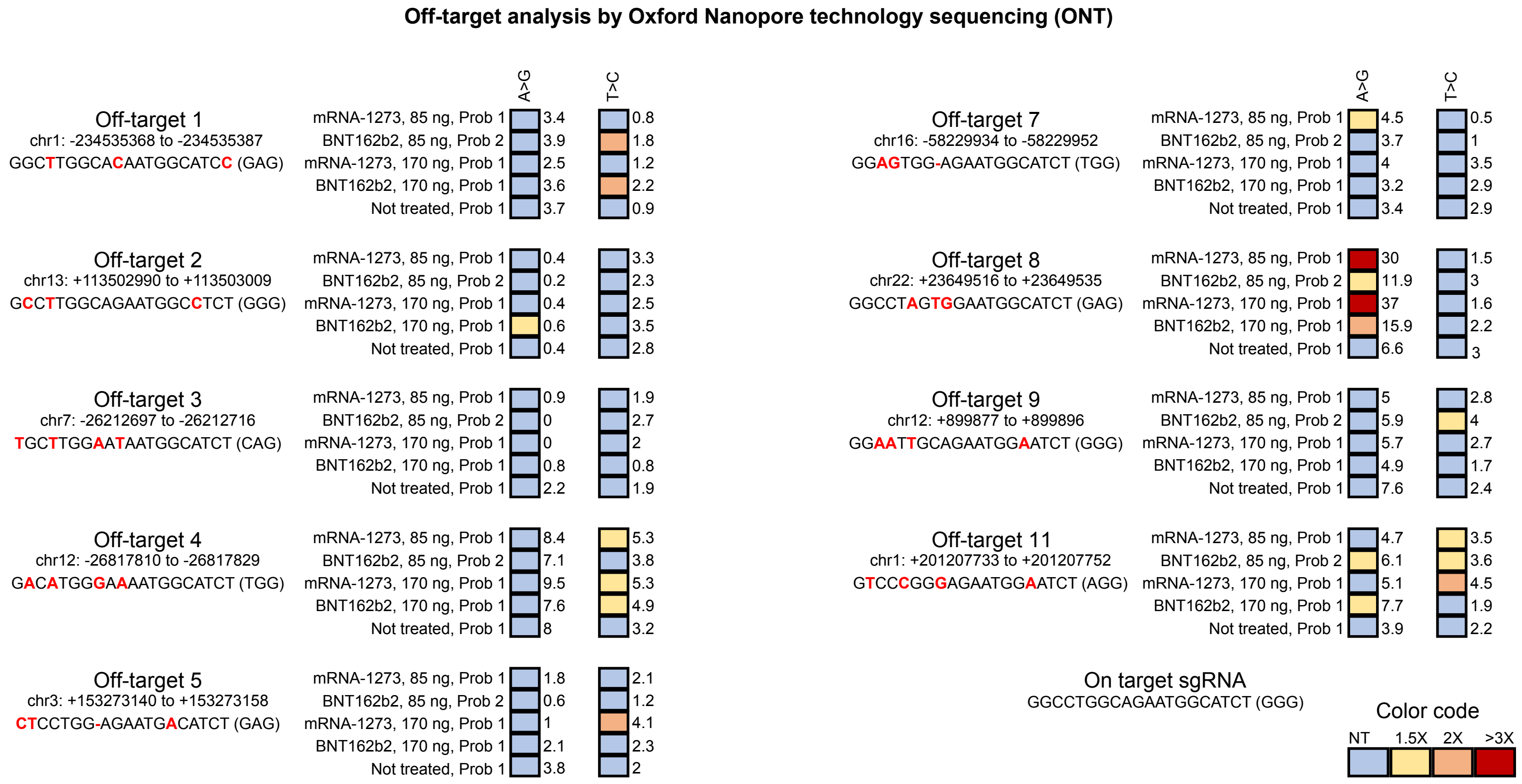

Figure S6: Analysis of the off-target activity through Oxford Nanopore Technology (ONT) sequencing.
To investigate the off-target effect in more detail, we generated a new list of 11 *in silico* predicted off-target sites using the software by IDT, CRISPOR, and Bencling. Then, we selected one non-treated fibroblast sample and four LNP-treated that showed a high on-target editing efficiency and amplified through PCR the 11 off-target sites in each one. We sequenced each DNA sample by ONT and analyzed the individual reads employing the CRISPResso2 software. The amplicon sizes were between 1400 nt and 2300 nt. We filtered out those reads that did not align with the genome of reference with at least 50% identity, but we kept all aligned reads, even those with low representation. This increases the sensitivity for rare events but also the noise in the analysis. We first compared the number of insertions and deletions in each treated sample to the non-treated one and saw no difference in that value. After that, we quantified all the adenine deamination activity on each of the nine off-target sites (as the sequencing of the off-target 6 and 10 filed due to highly repetitive flanking regions). When the putative off-target site falls on the positive strand of the locus, the base editing appears as an A>G change. When it occurs on the negative strand, the editing appears as a T>C change. Here we plotted the percentage of all aligned reads that presented any A>G (left) or T>C (right) conversion on the 20 nt region of each of the nine off-target sites. For each of the off-target sites, we noted the chromosomal location and the mismatches (in red) compared to the on-target sgRNA. The color of each square represents the fold change on A>G or T>C conversion percentage compared to the value of the non-treated sample for each of the off-target regions. To the right side of each square, we added the A>G or T>C conversion percentage for each sample. The ONT has some associated errors, and even the non-treated samples show variable levels of A>G and T>C change. As the ONT sequencing quality is partly related to the characteristics of each locus, for each off-target site we compared the conversion percentage of each treated sample to the conversion percentage of the corresponding non-treated sample.

Supplemental Tables

Table S1

sgRNA: ASL_1153	Software for prediction (order in the top 3 predictions)	Sequence	PAM	#MM	Gene	Locus	Editing
**On-target**		**GGCCTGGCAGAATGGCATCT**	**GGG**	**0**	**exon: *ASL***	**chr7:- 66092556-66092575**	**positive**
Off-target	IDT (1st)	AGCCTGG-AGAATGGCATCT	AAG	2	intergenic	chr2:+ 31113350-31113368	negative
Off-target	IDT (2nd) / Benchling (2nd)	TGCTTGTCAGAATGGCATCT	TGG	3	intergenic	chr2:- 29523682-29523701	negative
Off-target	IDT (3rd)	GGGCGGGCAGAATGACATCT	GGG	3	exon: *C1orf94*	chr1:- 34177482-34177501	negative
Off-target	CRISPOR (1st)	GGCCTGACAGAATGAAATCT	TGG	3	intron: *SCARB2*	chr4:+ 77128950-77128972	negative
Off-target	CRISPOR (2nd)	GGCCCAGCAAAATGACATCT	TGG	4	intergenic	chr4:- 7113139-7113161	negative
Off-target	Benchling (1st)	GGCCTGGCTGGATGGCATCT	TGG	2	intergenic	chr8:+ 142517669-142517688	negative
Off-target	Benchling (3rd)	TGCCTTGCACAATGGCATCT	TAG	3	intergenic	chr2:+ 184202763-184202782	negative

Table S1: off-target analysis by Sanger sequencing in hepatocyte-like cells and proband primary fibroblasts
Table S1 shows the *in silico* predicted off-target sites that were sequenced to check unwanted ABE-induced A•T to G•C mutations (off-target). The bases in red are the mismatches between each off-target sequence and the sgRNA ASL_1153. The number of mismatches is represented as #MM. We tested the presence of off-targets in the following hiPSC lines: Prob1-EDIT1, Prob1-EDIT2, Prob2-EDIT1, and Prob2-EDIT2. We also tested the presence of off-targets in the four fibroblast populations independently treated with 85ng of the mRNA-1273 LNP formulation carrying the ABE8e RNA vector, and the ASL_1153 sgRNA. We did not detect off-target activity in any of the seven loci analyzed in the four hiPSC or the four fibroblast populations.

Table S2

sgRNA: ASL_1153	Software for prediction (order of likelihood)	Sequence	PAM	#MM	Gene	Locus
**On-target**		**GGCCTGGCAGAATGGCATCT**	**GGG**	**0**	**exon: *ASL***	chr7:-66092556 to -66092575
ONT Off-target 1	Benchling (4th)	GGCTTGGCACAATGGCATCC	GAG	3	intergenic	chr1:-234535368 to 234535387
ONT Off-target 2	Benchling (5th)	GCCTTGGCAGAATGGCCTCT	GGG	3	intergenic	chr13:+113502990 to +113503009
ONT Off-target 3	Benchling (6th)	TGCTTGGAATAATGGCATCT	CAG	4	exon: *CBX3*	chr7:-26212697 to -26212716
ONT Off-target 4	Benchling (7th) / CRISPOR (5th)	GACATGGGAAAATGGCATCT	TGG	4	intergenic	chr12:-26817810 to -26817829
ONT Off-target 5	IDT (4th)	CTCCTGG-AGAATGACATCT	GAG	4	intergenic	chr3:+153273140 to +153273158
ONT Off-target 6	IDT (5th)	AGCTTGG-ATAATGGCATCT	GAG	4	intergenic	chr8:+129331504 to +129331522
ONT Off-target 7	IDT (6th)	GGAGTGG-AGAATGGCATCT	TGG	3	intergenic	chr16:-58229934 to -58229952
ONT Off-target 8	IDT (7th)	GGCCTAGTGGAATGGCATCT	GAG	3	*exon: *ASLP1*	chr22:+23649516 to +23649535
ONT Off-target 9	CRISPOR (3rd)	GGAATTGCAGAATGGAATCT	GGG	4	intron: *WNK1*	chr12:+899877 to +899896
ONT Off-target 10	CRISPOR (6th)	AGCCTGGCGGAAAGGAATCT	GGG	4	intron: *TAF2*	chr8:-119829280 to -119829299
ONT Off-target 11	CRISPOR (7th)	GTCCCGGGAGAATGGAATCT	AGG	4	exon: *IGFN1*	chr1:+201207733 to +201207752

Table S2: off-target sites for Oxford Nanopore technology sequencing analysis
Table S2 shows the *in silico* predicted off-target sites that were sequenced through Oxford Nanopore technology to check unwanted ABE-induced A•T to G•C mutations (off-target). The bases in red are the mismatches between each off-target sequence and the sgRNA ASL_1153. The number of mismatches is represented as #MM. We tested the presence of off-targets in four different LNP-treated fibroblast populations, the results are expressed in Figure S6.
**ASL* pseudogene ASLP1 (Refseq# NG_002637.6).

Table S3

Name	Sequence	Function	Comments	Vendor
**Single guide RNA**				
sgRNA: ASL_1153	mG*mG*mC*rCrUrGrGrCrArGrArArUrGrGrCrArUrCrUrGrUrUrUrUrArGrArGrCrUrArGrArArArUrArGrCrArArGrUrUrArArArArUrArArGrGrCrUrArGrUrCrCrGrUrUrArUrCrArArCrUrUrGrArArArArArGrUrGrGrCrArCrCrGrArGrUrCrGrGrUrGrCmU*mU*mU*rU	Target the ASL variant in chromosome 7	RNA oligo	IDT
sgRNA: ASL_1153_II	mG*mG*mG*rCrCrUrGrGrCrArGrArArUrGrGrCrArUrCrGrUrUrUrUrArGrArGrCrUrArGrArArArUrArGrCrArArGrUrUrArArArArUrArArGrGrCrUrArGrUrCrCrGrUrUrArUrCrArArCrUrUrGrArArArArArGrUrGrGrCrArCrCrGrArGrUrCrGrGrUrGrCmU*mU*mU*rU	Target the ASL variant in chromosome 7	RNA oligo	IDT
sgRNA: Site_16	mG*mG*mG*rArArUrArArArUrCrArUrArGrArArUrCrCrGrUrUrUrUrArGrArGrCrUrArGrArArArUrArGrCrArArGrUrUrArArArArUrArArGrGrCrUrArGrUrCrCrGrUrUrArUrCrArArCrUrUrGrArArArArArGrUrGrGrCrArCrCrGrArGrUrCrGrGrUrGrCmU*mU*mU*rU	Target the site 16 locus in chromosome 1. We used it as a non-targeting sgRNA control, given that it targets a locus unrelated to *ASL*.	RNA oligo	IDT
**PCR primers**				
*ASL*_Fw	GAGCTCAGGAATGGGTGCAA	Amplifies the genomic region around the pathogenic variant	DNA oligo	IDT
*ASL*_Rv	TCGCGCCCACTTTATTAGGG	Amplifies the genomic region around the pathogenic variant	DNA oligo	IDT
OT 1 Fw	CCTTGTAGGAGCAGTACCACC	Amplifies the genomic region containing a possible off-target	DNA oligo	IDT
OT 2 Fw	GCCCCGTTCAAGATCACAGA	Amplifies the genomic region containing a possible off-target	DNA oligo	IDT
OT 3 Fw	CTAGGCTGTCTTGGGGTGTG	Amplifies the genomic region containing a possible off-target	DNA oligo	IDT
OT 4 Fw	ACAGTTGTCAGCATAGCCCC	Amplifies the genomic region containing a possible off-target	DNA oligo	IDT
OT 5 Fw	GGCCACCACTTAGTGACTCC	Amplifies the genomic region containing a possible off-target	DNA oligo	IDT
OT 6 Fw	ACGGGAGAAGTGAGGAATGAC	Amplifies the genomic region containing a possible off-target	DNA oligo	IDT
OT 1 Rv	CCTTGTAGGAGCAGTACCACC	Amplifies the genomic region containing a possible off-target	DNA oligo	IDT
OT 2 Rv	GCCCCGTTCAAGATCACAGA	Amplifies the genomic region containing a possible off-target	DNA oligo	IDT
OT 3 Rv	CTAGGCTGTCTTGGGGTGTG	Amplifies the genomic region containing a possible off-target	DNA oligo	IDT
OT 4 Rv	ACAGTTGTCAGCATAGCCCC	Amplifies the genomic region containing a possible off-target	DNA oligo	IDT
OT 5 Rv	GGCCACCACTTAGTGACTCC	Amplifies the genomic region containing a possible off-target	DNA oligo	IDT
OT 6 Rv	ACGGGAGAAGTGAGGAATGAC	Amplifies the genomic region containing a possible off-target	DNA oligo	IDT
EBNA 1 Fw	ATCGTCAAAGCTGCACACAG	Amplifies the EBNA-1 region from the reprogramming plasmids in hiPSC	DNA oligo	Sigma
EBNA 1 Rv	CCCAGGAGTCCCAGTAGTCA	Amplifies the EBNA-1 region from the reprogramming plasmids in hiPSC	DNA oligo	Sigma
OriP Fw	TTCCACGAGGGTAGTGAACC	Amplifies the OriP region from the reprogramming plasmids in hiPSC	DNA oligo	Sigma
OriP Rv	TCGGGGGTGTTAGAGACAAC	Amplifies the OriP region from the reprogramming plasmids in hiPSC	DNA oligo	Sigma
ONT_OT_1_Fw	TGGCCTCACTGACGAGTTTC	Amplifies the genomic region containing a possible off target	DNA oligo	IDT
ONT_OT_1_Rv	CTTTGTCGGCCTCTCATGGT	Amplifies the genomic region containing a possible off target	DNA oligo	IDT
ONT_OT_2_Fw	GGCGGTGTTACTGGAGACAA	Amplifies the genomic region containing a possible off-target	DNA oligo	IDT
ONT_OT_2_Rv	TCTTTTGTGCGGTGTCTGGA	Amplifies the genomic region containing a possible off-target	DNA oligo	IDT
ONT_OT_3_Fw	TGGCTGTCGGTTGACATGAA	Amplifies the genomic region containing a possible off-target	DNA oligo	IDT
ONT_OT_3_Rv	GTCTATCATCACACTGAGCCCT	Amplifies the genomic region containing a possible off-target	DNA oligo	IDT
ONT_OT_4_Fw	GTGGCGAAAGGGAACTCAGA	Amplifies the genomic region containing a possible off-target	DNA oligo	IDT
ONT_OT_4_Rv	CCCATGCGAAGAATGAGCCT	Amplifies the genomic region containing a possible off-target	DNA oligo	IDT
ONT_OT_5_Fw	AGAGACGGCTCTTTCCCTCT	Amplifies the genomic region containing a possible off-target	DNA oligo	IDT
ONT_OT_5_Rv	GTGATGAGCAGCTAGGGAGC	Amplifies the genomic region containing a possible off-target	DNA oligo	IDT
ONT_OT_6_Fw	AACACTTCCAGCTTGCCTGA	Amplifies the genomic region containing a possible off-target	DNA oligo	IDT
ONT_OT_6_Rv	ATTCCTGGTGGAGCATTCGT	Amplifies the genomic region containing a possible off-target	DNA oligo	IDT
ONT_OT_7_Fw	ACCGCGAAACAAAATCCTGG	Amplifies the genomic region containing a possible off-target	DNA oligo	IDT
ONT_OT_7_Rv	TGCCCTTTTTCCCCAACAGT	Amplifies the genomic region containing a possible off-target	DNA oligo	IDT
ONT_OT_8_Fw	CAGGGCAGAGGCTGTGATAG	Amplifies the genomic region containing a possible off-target	DNA oligo	IDT
ONT_OT_8_Rv	GGGGTCCTCAAACGAAACCT	Amplifies the genomic region containing a possible off-target	DNA oligo	IDT
ONT_OT_9_Fw	CACATTAGGGCCAGTTCATGC	Amplifies the genomic region containing a possible off-target	DNA oligo	IDT
ONT_OT_9_Rv	TAGTCTCCCAACCCGCCTTA	Amplifies the genomic region containing a possible off-target	DNA oligo	IDT
ONT_OT_10_Fw	TAGGTGGGAACAGGGCTGAA	Amplifies the genomic region containing a possible off-target	DNA oligo	IDT
ONT_OT_10_Rv	ACTGCGGTAATCTAGGGGAGA	Amplifies the genomic region containing a possible off-target	DNA oligo	IDT
ONT_OT_11_Fw	AACTGTGGGATGCTCGACTG	Amplifies the genomic region containing a possible off-target	DNA oligo	IDT
ONT_OT_11_Rv	GGCCCTGGAAGTTCTGATCC	Amplifies the genomic region containing a possible off-target	DNA oligo	IDT
**qPCR primers**				
ASL Fw	CTCTCAACAGCATGGATGCCAC	quantifies ASL expression in qPCR	DNA oligo	Sigma
ASL Rv	CTTGGTGCAGTAGAGGATGAGG	quantifies ASL expression in qPCR	DNA oligo	Sigma
AFP Fw	CGCTGCAAACGATGAAGCAAG	quantifies AFP expression in qPCR	DNA oligo	Sigma
AFP Rv	AATCTGCAATGACAGCCTCAAG	quantifies AFP expression in qPCR	DNA oligo	Sigma
Albumin Fw	GGAAAAGTGGGCAGCAAATGT	quantifies Albumin expression in qPCR	DNA oligo	Sigma
Albumin Rv	GGTTCAGGACCACGGATAGA	quantifies Albumin expression in qPCR	DNA oligo	Sigma
HNF1a Fw	GGGCTTCTTGGACAACCTTTTCA	quantifies HNF1a expression in qPCR	DNA oligo	Sigma
HNF1a Rv	CGTATGGACACCCGGCTCAT	quantifies HNF1a expression in qPCR	DNA oligo	Sigma
CycloG Fw	TCTTGTCAATGGCCAACAGAG	quantifies CycloG expression in qPCR	DNA oligo	Sigma
CycloG Rv	GCCCATCTAAATGAGGAGTTG	quantifies CycloG expression in qPCR	DNA oligo	Sigma
Serpina1 Rv	AGCTGGCACACCAGTCCAAC	quantifies Serpina expression in qPCR	DNA oligo	Sigma
Serpina1 Fw	TGGCTGGTTGAGGGTACGGA	quantifies Serpina expression in qPCR	DNA oligo	Sigma
APOC3 Fw	CCGCCAAGGATGCACTGAG	quantifies APOC3 expression in qPCR	DNA oligo	Sigma
APOC3 Rv	CTCCAGTAGTCTTTCAGGGAACT	quantifies APOC3 expression in qPCR	DNA oligo	Sigma
APOA2 Fw	ATGTGTGGAGAGCCTGGTTTCTCA	quantifies APOA2 expression in qPCR	DNA oligo	Sigma
APOA2 Rv	AAGCTCTGGGCTCTTGACCTTCT	quantifies APOA2 expression in qPCR	DNA oligo	Sigma
OCT4 Fw	TTGGGCTCGAGAAGGATGTG	quantifies OCT4 expression in qPCR	DNA oligo	Sigma
OCT4 Rv	GTGAAGTGAGGGCTCCCATA	quantifies OCT4 expression in qPCR	DNA oligo	Sigma
SOX2 Fw	GCCCTGCAGTACAACTCCAT	quantifies SOX2 expression in qPCR	DNA oligo	Sigma
SOX2 Rv	CTCAGCCTCCAGCAGATGC	quantifies SOX2 expression in qPCR	DNA oligo	Sigma
Nanog Fw	TGCCCTGCTGCGAGTAGGA	quantifies Nanog expression in qPCR	DNA oligo	Sigma
Nanog Rv	TAGATTTCATTCTCTGGTTCTGG	quantifies Nanog expression in qPCR	DNA oligo	Sigma
**Primers for cloning**				
ABE8e-Fw	CTCAACTTTGGCAGATCTGAATTCCTGCAGCCCGGGATCCACAGCCACCATGAAACGGACAGCCGACGGAAGCGAG	Cloning ABE8e ORF into IVT T3 backbone	DNA oligo	Sigma
ABE8e-Rv	CTTGAGGCTGGTTTAGTGGTAACCAGATCCGCGGCCGCTCACCTACTTAGACTTTCCTCTTCTTCTTGGGCTCG	Cloning ABE8e ORF into IVT T3 backbone	DNA oligo	Sigma

Table S3: primers and sgRNAs

Table S3 shows the sgRNA ASL_1153 employed to target the ASL c.1153C>T pathogenic variant, and the sgRNA Site_16 used as a control guide targeting a locus unrelated to ASL. The table also shows the DNA primers for PCR and qPCR. The sequences are notated in 5'-to-3' orientation. In the "single guide RNA section", the protospacer is underlined, and the non-underlined sequence is the canonical CRISPR-Cas9 tracrRNA. "r_" (rA, rC, rG, rU) refers to ribonucleic bases. "m_*" (mC*, mA*, mG*, mU*) refers to phosphorothioated 2'-O-methyl RNA bases.

Supplemental Methods

Fibroblast culture

The skin biopsies were manually disaggregated, seeded, and cultured under glass coverslips in a 60 mm dish. The culture medium consisted of DMEM (Sigma, 6546) supplemented with 20% fetal bovine serum (FBS, Life Technologies, 10106-169), 1X GlutaMAX (Life Technologies, 35050-038), and 1% penicillin-streptomycin (Life Technologies, 15140-122) until the fibroblast cells formed a monolayer, after which the cells were passaged with TrypLETM Select Enzyme (Thermo Fisher Scientific; 12563029). After passaging the fibroblasts from the plates containing the skin biopsies, they were cultured in DMEM supplemented with 10% FBS and 1X GlutaMAX.

Incucyte and toxicity experiments

We followed the fibroblast population using the Incucyte® Live-Cell Analysis System, which automatically images the cells to assess their confluency and the number of dead cells, stained by the CytoTox membrane integrity dye (Sartorius; Cat. No. 4633). The fibroblasts were plated on 24-well plates and cultured with DMEM FBS 10%. We programmed the Incucyte® to take 16 pictures per well every 2 hrs, from time 0 to 70 hrs. Either different concentrations of lipid nanoparticles or an equal volume of PBS was added into each well at the 2 hrs time point. The confluence was calculated using the Incucyte® software and normalized to time 0. The absolute number of green positive (dead) cells was counted using the same software.

Direct argininosuccinate lyase activity assay

Fibroblasts were cultured in 100 mm plates until 80 % confluency, the cells were then washed with PBS, collected using a cell scraper, and centrifuged at 200g for 4 minutes. The supernatant was discarded, and the cell pellets were stored at -80°C. Protein samples were extracted from frozen fibroblast pellets by lysis with a buffer containing PBS, 0.1% TritonX-100 (Sigma-Aldrich, X100), and protease inhibitors (Roche, 04693124001), centrifuging 10,000 RCF for 10 minutes at + 4°C. The protein concentration in the supernatant was measured by PierceTM BCA Protein assay kit (Thermo Scientific; 23227). 0.6 mg/ml of total protein lysate was incubated in a physiological buffer consisting of 100 mM Tris-HCl (pH 7.2) and 0.3 mM argininosuccinate disodium salt (73097; Sigma-Aldrich) for 15 minutes at +37°C and 5 minutes at +95°C. The incubation was performed in the S1000 Thermal Cycler (Biorad) and the final volume per each reaction was 120 ul. The ASL enzyme activity was estimated by measuring the total amount of fumarate (in nanomoles) that formed during the incubation using the Fumarate Assay kit from Sigma-Aldrich (MAK060). The amount of fumarate in each reaction was measured in duplicates, loading 50 ul of the reaction supernatant into 96-well plate wells for a spectrophotometric measurement with the EnSpire® Multimode Plate Reader (PerkinElmer) at 450 nm. The unspecific reaction background was determined by a parallel reaction where the incubation order was reversed (5 minutes at +95°C and 15 minutes at +37°C). This background read was subtracted from the final result.

HepG2 cell line

HepG2 (Knowles BB, Aden DP. US Patent 4,393,133 dated Jul 12, 1983) is a commercial cell line isolated from a hepatocellular carcinoma of a 15-year-old, white male. The HepG2 line presents a high expression of AFP, HNF1α, SERPINA1, Albumin, APOA2, and APOC31; hence it was used as a positive control for the qPCR of the mentioned genes.

The cells were cultured in 12 well plates with no coating. The media consisted of DMEM (Sigma, 6546) supplemented with 10% fetal bovine serum (FBS, Life Technologies, 10106-169) inactivated for 30min at 56°C, 1X GlutaMAX (Life Technologies, 35050-038), and 1% penicillin-streptomycin (Life Technologies, 15140-122).

Sample preparation for metabolomic analysis

Hepatocyte-like cells and primary fibroblasts were cultured in 12 well plates with 0.75 mL of their respective media. Hepatocyte-like cells were harvested at day 18 of their differentiation protocol, fibroblasts were harvested one day after reaching confluency.

For harvesting, all the media (which was in contact with the cells for 24 hrs) in the well was collected and placed in a tube on ice. Then, we washed the cells once with cold PBS and added 255 µl of +4°C extraction buffer (Acetonitrile/dH2O 80:20) on top of the cells. All the cells in the well were detached and homogenized using a pipette and then transferred into a tube on ice. The samples were vortexed for 10 sec at full speed. At the same time, 50 µl of each media sample was transferred into a tube with 450 µl of +4°C extraction buffer (Acetonitrile/dH2O 80:20), and then vortexed. Then, all the cell lysate samples and the diluted media samples were centrifuged (15,800 g for 10 min at +4°C). We finally transferred 100 µl of the supernatant of each sample to a SureSTART 0.3 ml glass screw top micro vial (Thermo Scientific) and stored them at -80°C for no longer than one month until the metabolomic analysis.

sgRNA design

The sgRNA ASL_1153 and ASL_1153_II were designed using the web tool Benchling (https://benchling.com). Integrated DNA Technologies manufactured the sgRNA. The sequence of the sgRNA is listed in Table S3.

Determination of on-target editing, Sanger sequencing

We extracted genomic DNA from a bulk population of millions of cells, amplified the genomic locus around the *ASL* variant by PCR, purified and Sanger sequenced the PCR product. We analyzed on-target editing using the web tool EditR2.

Off-target analysis, Sanger sequencing

The seven most likely off-target loci for the sgRNA ASL_1153 were predicted by the web tools provided by (IDT Integrated DNA Technologies), Benchling (https://benchling.com), and CRISPOR3. See Table S1 for the top seven list of in silico predicted off-targets for the sgRNA ASL_1153. DNA samples from monoclonal edited hiPSC lines and edited bulk fibroblast populations were collected. Each of the seven loci in each sample was amplified by PCR (primers described in Table S3). The off-target editing was estimated from the Sanger sequencing data using the web tool EditR.

On-target and off-target analysis, Oxford Nanopore technology sequencing

We performed a 30-cycle PCR for each primary fibroblast DNA sample. We then purified the PCR product through electrophoresis in a 1% agarose TAE gel and column purified the gel band eluting the DNA in a concentration of 30 ng/ul. The samples were packaged according to the company guidelines (https://www.plasmidsaurus.com) and shipped them at room temperature. We obtained between 300 and 2,000 raw reads per sample and aligned them to the genome of reference and the sgRNA sequence using the CRSPResso2 software. For statistical analysis of the on-target editing efficiency, we considered all the reads that aligned with the genome of reference with at least 50% identity and had a representation equal to or above 1%, and computed the allele frequency into three categories: precisely edited reads, those that contained any bystanders or indels, and those that remained unchanged. For the statistical analysis of the off-target, we considered all reads that aligned with the genome of reference with at least 50% identity. Using CRISPResso2, we determined the A>G or T>C conversion percentages for every adenine or thymidine within the 20 nt region of each of the nine off-target sites. We then separately added all the A>G or T>C conversion percentages to create a parameter that reflects the potential ABE editing for each off-target site.

Western blot analysis

Cell samples were collected from culture plate wells by trypsinization or scraping and the cell pellets were lysed in a buffer containing PBS, 0.1 % TritonX-100, and protease inhibitors. The samples were centrifuged at 4°C for 10 minutes, 10000 g, and the supernatant was collected. The protein concentration of each sample was quantified by the Pierce BCA Protein Assay Kit (ThermoFisher, 23227). The samples were combined with 4x Laemmli Sample Buffer (Biorad, 1610746), and denatured at +95°C for 5 minutes. For the Western blot, 30 µg of total protein was seeded in each well of a Mini-Protean TGX Precast gel (Biorad, 456-8034). Primary antibodies: anti-ASL antibody produced in rabbit (Sigma-Aldrich, HPA016646, 1:500 dilution in 5 % milk-TBST), Human Serum Albumin antibody produced in mouse (R&D systems; MAB1455; 1:500 dilution in 5 % milk-TBST), anti-actin Antibody produced in mouse (Sigma-Aldrich; A3853; 1:1000 dilution in 5 % milk-TBST). Secondary antibodies: IRDye 800CW anti-rabbit IgG (LI-COR, 926-32213, 1:1,000 dilution in 5 % milk-TBST) and IRDye 680RD anti-mouse IgG (LI-COR, 926-68072, 1:1,000 dilution in 5 % milk-TBST). The ladder employed was Precision Plus Protein Dual Color Standards (Biorad, 1610374). After the treatment to simultaneously stain ASL, albumin, and actin, the membrane was imaged in the Odyssey (LI-COR), ASL was visible in the green channel, whereas albumin and actin were captured in the red channel.

Immunocytochemistry

HiPSCs were plated on round coverslips coated with Matrigel in 24-well plates and cultured to 50 % confluency when they were fixed. HiPSC-derived hepatocytes were differentiated on round coverslips coated with Matrigel in 24-well plates and fixed on day 18.

Cells were fixed with 4 % PFA in PBS for 15 minutes, permeabilized with 0.5 % Triton X-100 for 10 minutes, and blocked with an Ultravision blocker (Thermo Scientific) for 10 minutes. They were then incubated with primary antibodies diluted in 0.1% Tween in PBS for 24 hours at 4°C on a Stuart SSL4 seesaw rocker. Then, the plates were treated with Hoechst 33342 (Thermo Fisher Scientific), and the secondary antibodies were diluted in 0.1 % Tween in PBS in the dark at room temperature for 30 minutes on the seesaw rocker.

Primary antibodies: OCT4 (1:500 goat, polyclonal, Santa Cruz, sc-8628), TRA-1-60 (1:500 mouse, monoclonal, Thermo Fisher Scientific, MA1-023), SSEA (1:1000 mouse, monoclonal, Millipore, MAB4304), SOX17 (1:500 goat, polyclonal, R&D Systems, AF1924), α-SMA (1:500 mouse, monoclonal, Sigma, A2547), and β-tubulin III (1:500 rabbit, polyclonal, Abcam, Ab18207), AFP (1:300 rabbit, polyclonal, Agilent, A000829-2), Albumin (1:33 mouse, monoclonal, R&D Systems, MAB1455), HNF4α (1:500 rabbit, monoclonal, Cell Signalling Technology, 3113).

Secondary antibodies: Alexa Fluor 488 anti-goat (1:500 donkey, Invitrogen, A11055), Alexa Fluor 488 anti-mouse (1:500 donkey, Invitrogen, A21202) and anti-rabbit (1:500 donkey, Invitrogen, A21206), and Alexa Fluor 594 anti-mouse (1:500 donkey, Invitrogen, A21203) and anti-rabbit (1:500 donkey, Invitrogen, A21207).

Karyotyping

Samples were prepared for karyotyping as described previously[4]. 2.0 x 106 cells were suspended in a medium supplemented with 0.1 µg/mL KaryoMAX Colcemid Solution in PBS and incubated for 4 hrs at 37 °C. Cells were resuspended in 0.075 M KCl and incubated at 37 °C for 10 min. Fixative (3:1 ratio of methanol and acetic acid) was added dropwise to the cell suspension. Fixation was repeated three times before storing the samples at 4 °C until shipping. Karyotyping was performed as a service by Ambar in Barcelona, Spain.

Supplemental references

1. Rouillard, A.D., Gundersen, G.W., Fernandez, N.F., Wang, Z., Monteiro, C.D., McDermott, M.G., and Ma'ayan, A. (2016). The harmonizome: a collection of processed datasets gathered to serve and mine knowledge about genes and proteins. Database 2016, baw100. 10.1093/database/baw100.
2. Kluesner, M.G., Nedveck, D.A., Lahr, W.S., Garbe, J.R., Abrahante, J.E., Webber, B.R., and Moriarity, B.S. (2018). EditR: A Method to Quantify Base Editing from Sanger Sequencing. CRISPR J 1, 239–250. 10.1089/crispr.2018.0014.
3. Concordet, J.P., and Haeussler, M. (2018). CRISPOR: Intuitive guide selection for CRISPR/Cas9 genome editing experiments and screens. Nucleic Acids Res 46, W242–W245. 10.1093/nar/gky354.
4. Howe, B., Umrigar, A., and Tsien, F. (2014). Chromosome preparation from cultured cells. J Vis Exp, e50203–e50203. 10.3791/50203.
