# Supplementary material for: Genetic and functional correction of argininosuccinate lyase deficiency using CRISPR adenine base editors
Source: Am J Hum Genet. 2024 Apr 4;111(4):714–28. doi: 10.1016/j.ajhg.2024.03.004 (PMC11023919; doi:10.1016/j.ajhg.2024.03.004)
Supplement: Document S2. Article plus supplemental information [file mmc3.pdf]

Genetic and functional correction of argininosuccinate lyase deficiency using CRISPR adenine base editors

Graphical abstract

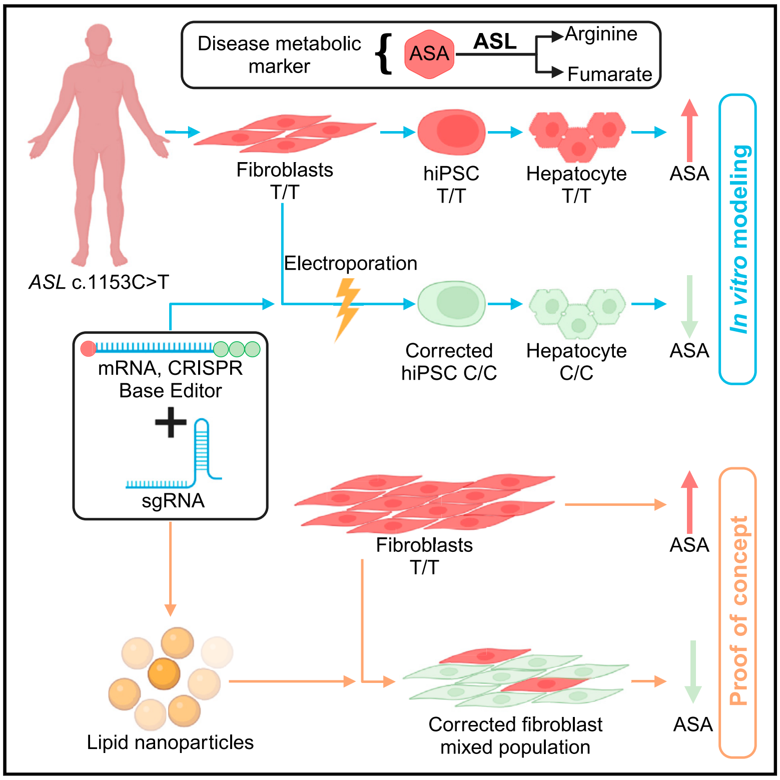

Authors

Sami Jalil, Timo Keskinen, Juhana Juutila, ..., Timo Otonkoski, Mervi E. Hyvönen, Kirmo Wartiovaara

Correspondence

kirmo.wartiovaara@helsinki.fi

Harnessing CRISPR to correct a metabolic inherited disease, we developed a therapeutically relevant method to precisely edit the Finnish Founder pathogenic variant responsible for argininosuccinate lyase deficiency. By lipid-nanoparticle-mediated delivery of the adenosine base editor RNA, we achieve efficient gene editing and metabolic normalization in patient fibroblasts.

Jalil et al., 2024, The American Journal of Human Genetics *111*, 714–728
April 4, 2024 © 2024 The Author(s).
https://doi.org/10.1016/j.ajhg.2024.03.004

CellPress

Genetic and functional correction of argininosuccinate lyase deficiency using CRISPR adenine base editors

Sami Jalil,[1,6] Timo Keskinen,[1,6] Juhana Juutila,[2,4] Rocio Sartori Maldonado,[1] Liliya Euro,[1] Anu Suomalainen,[1] Risto Lapatto,[1,5] Emilia Kuuluvainen,[2,4] Ville Hietakangas,[2,4] Timo Otonkoski,[1,5] Mervi E. Hyvönen,[1,5] and Kirmo Wartiovaara[1,3,*]

Summary

Argininosuccinate lyase deficiency (ASLD) is a recessive metabolic disorder caused by variants in *ASL*. In an essential step in urea synthesis, ASL breaks down argininosuccinate (ASA), a pathognomonic ASLD biomarker. The severe disease forms lead to hyperammonemia, neurological injury, and even early death. The current treatments are unsatisfactory, involving a strict low-protein diet, arginine supplementation, nitrogen scavenging, and in some cases, liver transplantation. An unmet need exists for improved, efficient therapies. Here, we show the potential of a lipid nanoparticle-mediated CRISPR approach using adenine base editors (ABEs) for ASLD treatment. To model ASLD, we first generated human-induced pluripotent stem cells (hiPSCs) from biopsies of individuals homozygous for the Finnish founder variant (c.1153C>T [p.Arg385Cys]) and edited this variant using the ABE. We then differentiated the hiPSCs into hepatocyte-like cells that showed a 1,000-fold decrease in ASA levels compared to those of isogenic non-edited cells. Lastly, we tested three different FDA-approved lipid nanoparticle formulations to deliver the ABE-encoding RNA and the sgRNA targeting the *ASL* variant. This approach efficiently edited the *ASL* variant in fibroblasts with no apparent cell toxicity and minimal off-target effects. Further, the treatment resulted in a significant decrease in ASA, to levels of healthy donors, indicating restoration of the urea cycle. Our work describes a highly efficient approach to editing the disease-causing *ASL* variant and restoring the function of the urea cycle. This method relies on RNA delivered by lipid nanoparticles, which is compatible with clinical applications, improves its safety profile, and allows for scalable production.

Introduction

Argininosuccinate lyase deficiency (ASLD [MIM: 207900]), also known as argininosuccinic aciduria, is an autosomal-recessive urea cycle disorder caused by loss-of-function variants in *ASL* (MIM: 608310).[1–3] ASLD, like other urea cycle defects, impairs the disposal of nitrogen and leads to hyperammonemia. It can present as a neonatal life-threatening condition with lethargy, vomiting, seizures, and coma. These symptoms can also arise later in life during a catabolic state. The long-term sequelae include epilepsy, developmental impairment, and liver disease.[1–3] The incidence of ASLD in the US and Europe has been estimated in the most recent reports to be from 1:190,000 to 1:219,000 live births,[4,5] and in Finland 1:144,000.[6]

The ASL enzyme catalyzes the breakdown of argininosuccinate (ASA) into arginine and fumarate.[7] This is an essential cytosolic step in the urea cycle and its disruption leads to a potentially toxic ASA accumulation.[2,8] Moreover, the disruption of ASL activity impairs the biosynthesis of arginine, which becomes an essential amino acid that needs to be acquired through diet.[2] The hallmark of ASLD, and a diagnostically helpful distinction from other urea cycle defects, is the accumulation of ASA in plasma and urine.[9]

The pathophysiology of ASLD remains partially unclear. It involves hyperammonemia causing irreversible damage to the developing central nervous system,[10] the potential hepatic and neurotoxic effects of ASA and its related metabolite guanidinosuccinate,[2,8,11,12] and the compromised nitric oxide (NO) synthesis.[13,14] ASLD variants disrupt NO synthesis through two mechanisms: they interfere with the structural role of ASL in the multiprotein complex required for NO production and impair the catalytic activity of ASL, restricting the recycling of citrulline into the arginine supply available for NO synthesis.[13] The current options for disease management are a protein-restricted diet combined with arginine supplementation and nitrogen-scavenging medications or liver transplantation in severe cases.[2]

The disease severity varies depending on the variant and the residual ASL enzyme activity.[15] The Finnish founder *ASL* variant (c.1153C>T [GenBank: NM_000048.4] [p.Arg385Cys]; rs28940286) is enriched in Finland, and according to the gnomAD database, the allele frequency is 0.0004832. This variant is homozygous or compound heterozygous in 70% of the identified ASLD individuals in

[1]Stem Cells and Metabolism Research Program, Faculty of Medicine, University of Helsinki, Helsinki, Finland; [2]Faculty of Biological and Environmental Sciences University of Helsinki, Helsinki, Finland; [3]Clinical Genetics, Helsinki University Hospital, Helsinki, Finland; [4]Institute of Biotechnology, Helsinki Institute of Life Science, University of Helsinki, Helsinki, Finland; [5]New Children's Hospital, Pediatric Research Center, University of Helsinki and Helsinki University Hospital, Helsinki, Finland
[6]These authors contributed equally
*Correspondence: kirmo.wartiovaara@helsinki.fi
https://doi.org/10.1016/j.ajhg.2024.03.004.
© 2024 The Author(s). This is an open access article under the CC BY license (http://creativecommons.org/licenses/by/4.0/).

Finland and results in a cysteine replacing the positively charged arginine at a crucial position near the enzyme's active site. This arginine interacts with the negatively charged glutamine 389 to stabilize the carboxy-terminus helix bundle of the protein.[16] Substitutions in arginine 385 may also interfere with residues near the active site, such as glutamine 399, thus impairing enzyme activity.[16] The enzyme activity of this variant is not exactly known and can depend on the assay used, but a direct ASL enzyme activity assay in fibroblasts showed no activity.[17,18]

The CRISPR-Cas9 base editors have emerged as powerful tools for precisely editing point mutations with high efficiency and collectively possess the potential to reverse up to 60% of all pathogenic point mutations.[19] The adenine base editor (ABE)[20] catalyzes the nucleotide transition of a targeted A-T to a G-C base pair within a programmable and narrow target window without requiring a DNA donor template nor inducing DNA double-strand breaks, thereby providing a safer alternative to approaches that rely on such breaks and carry a higher risk of genomic rearrangements, insertions, and deletions.[21] The ABE is not only safer, but also maintains a very high on-target efficiency while presenting a lower off-target activity than that observed with canonical SpCas9.[21–23] Animal and pre-clinical studies have demonstrated the promise of base editors in editing variants associated with diseases, such as Duchenne muscular dystrophy (MIM: 310200)[24] and hereditary tyrosinemia type 1 (MIM: 276700).[25] Similarly, ongoing clinical trials are already underway to assess their therapeutic potential in individuals with familial hypercholesterolemia, lymphoblastic leukemia, and sickle cell disease (ClinicalTrials.gov: NCT05398029, NCT05885464, and NCT05456880).

We hypothesized that CRISPR base editors could offer a potential therapy for ASLD and tested this approach *in vitro* with the Finnish founder *ASL* variant c.1153C>T. By reprogramming ASLD fibroblasts into human-induced pluripotent stem cells (hiPSCs) and simultaneously editing the variant, we rescued the disrupted urea cycle in the genetically edited hiPSC-derived hepatocyte-like cells. To enable *in vivo* delivery, we encapsulated an optimized mRNA construct encoding the improved ABE version, ABE8e,[26] along with the variant-targeting single-guide RNA (sgRNA), into lipid nanoparticles (LNPs). We tested three different US Food and Drug Administration (FDA)-approved LNP formulations,[27–29] similar to those employed in COVID-19 mRNA vaccines, and they successfully delivered the ABE8e mRNA construct into the fibroblasts, editing the *ASL* variant, rescuing the enzyme activity, and restoring ASA levels to that of healthy donors without any evident toxicity or off-target effects.

Material and methods

Ethical permit

Generation of fibroblast and hiPSC lines from skin biopsies was approved by the Coordinating Ethics Committee of the Helsinki and Uusimaa Hospital District upon informed consent of the donors or their guardians (diary no.: HUS/2754/2019).

Biopsies

Skin biopsy samples were collected from two unrelated voluntary donors carrying the Finnish founder variant *ASL* variant (c.1153C>T [p.Arg385Cys]) in homozygosity. The child's biopsy was taken under general anesthesia for an unrelated reason.

Development of T3_ABE8e_IVT plasmid

ABE8e was a gift from David Liu (Addgene: 138489; http://n2t.net/addgene:138489; RRID: Addgene_138489). We cloned the ABE8e open reading frame into a backbone containing a T3 promoter for *in vitro* transcription, and *Xenopus* 5′ and 3′ UTRs, as we previously did for the ABEmax.[30] For the cloning, we used the NEB HighFidelity Assembly kit (New England BioLabs, catalog no.: E5520S), primers are detailed in Table S3.

Our plasmids for T3 *in vitro* transcription of the ABE8e and the ABEmax were deposited in Addgene (plasmids 201676 and 171761).

In vitro transcription ABEmax, and ABE8e

Employing the ABEmax or the ABE8e IVT plasmid (Addgene: 201676 and 171761) as a DNA template, T3 RNA transcription was performed according to the manufacturer's protocol (mMESSAGE mMACHINE T3 Transcription Kit, Thermo Fisher Scientific, Invitrogen, catalog no.: AM1348). The plasmid was linearized by SfiI restriction (Thermo Fisher Scientific, catalog no.: FD1824).

Fibroblast reprogramming and simultaneous ABEmax-mediated editing

Fibroblast electroporation, reprogramming, ABEmax editing, and hiPSC line generation were performed as previously described.[30]

Hepatocyte differentiation

Before hepatocyte differentiation, we cultured hiPSCs in Essential 8 medium (E8, Thermo Fisher Scientific, A1517001). On the day before the start of the differentiation, cells were treated with 0.5 mM EDTA in PBS, resuspended into single cell with DMEM, and seeded onto Matrigel-coated (Corning, 356231) 12-well plates (800,000 cells per well) containing E8 and 10 μM rho-associated protein kinase (ROCK) inhibitor (Y-27632, Selleckchem). On day 0, we changed the medium to a definitive endoderm induction medium consisting of Basal 1 media (MCDB 131 free from L-Glutamine [PAN BIOTECH, P04-80057] supplemented with 5 mg/mL Bovine Serum Albumin [Sigma, A7030], 1.5 mg/mL $NaHCO_3$ [Sigma], Glucose and Glutamax with 0.1 μg/mL Activin A [Qkine, Qk001], and 3 μM CHIR-99021 [Tocris, 4423]). On day 1, we changed the medium to Basal 1 media with 0.1 μg/mL activin A and 0.3 μM CHIR. On day 2, we changed the medium to Basal 1 media with 0.1 μg/mL activin A and estimated the definitive endoderm induction efficiency by flow cytometry using BD PharmingenTM PE Mouse Anti-Human CD184 (#555974) to stain a definitive endoderm marker CXCR4 and BD PharmingenTM PE Mouse IgG1 (#555574) as a control. We proceeded with the differentiation if the cell population reached a threshold of 80% CD184-positive cells at this stage. From day 3 until day 18, we followed the hepatocyte differentiation protocol previously described.[31]

LNP formulation

LNPs were generated by mixing an organic phase and an aqueous phase in a volume ratio of 1:2 using the NanoAssemblr Spark (Precision Nanosystems) according to the vendor's instruction. These two phases mix within the microfluidic channels of the cartridge to form LNPs. The LNPs were immediately dispersed in a volume of neutral PBS buffer equal to the sum of the two input phases.

The organic phase consisted of a 45 mM mix of different lipids in ethanol according to the LNP formulations.

ALN-18328 (Onpattro/patisiran)

The solution consisted of DLin-MC3-DMA/DSPC/Cholesterol/DMG-PEG2000 (50/10/38.5/1.5 mol/mol). DLin-MC3-DMA (MedKoo Biosciences Inc. CAT#: 555308), DSPC (Avanti Polar Lipids CAT#: 850365), Cholesterol (Sigma-Aldrich C3045), DMG-PEG 2000 (Sigma-Aldrich 880151P). Molar N/P: 3. The mixture was prepared as previously described.[28]

mRNA-1273 (Moderna)

The solution consisted of SM-102/DSPC/Cholesterol/DMG-PEG2000 (50/10/38.5/1.5 mol/mol). SM-102 (BroadPharm CAT#: BP-25499). Molar N/P: 6. The mixture was prepared as previously described.[29,32]

BNT162b2 (Pfizer-BioNTech)

The solution consisted of ALC-0315/DSPC/Cholesterol/ALC-0159 (46.3/9.4/42.7/1.6 mol/mol). ALC-0315 (MedKoo Biosciences Inc. CAT#: 556006), ALC-0159 (BroadPharm CAT#: BP-25711). Molar N/P: 6. The mixture was prepared as previously described.[29,32]

The aqueous phase consists of sodium citrate buffer (pH 4, 65 mM) containing the sgRNA (IDT, Integrated DNA Technologies) and the ABE8e RNA construct in a 1:2 mass ratio.

Independently of the formulation, the total RNA concentration in the resulting LNPs was 17 mg/ml, considering the mass of the RNA construct and the sgRNA.

Metabolomics analysis

Samples were analyzed on a Thermo Q Exactive Focus Quadrupole Orbitrap mass spectrometer coupled with a Thermo Dionex UltiMate 3000 HPLC system (Thermo Fisher Scientific). The high-performance liquid chromatography (HPLC) was equipped with a hydrophilic ZIC-pHILIC column (150 × 2.1 mm, 5 μm) with a ZIC-pHILIC guard column (20 × 2.1 mm, 5 μm, Merck Sequant). A 5 μL sample was injected into the liquid chromatography-mass spectrometry (LC-MS) instrument after quality controls in randomized order having every tenth sample as blank. A linear solvent gradient was applied in decreasing organic solvent (80%–35%, 16 min) at 0.15 mL min–1 flow rate and 45°C column oven temperature. Mobile phases were aqueous 200 mmol per liter ammonium bicarbonate solution (pH 9.3, adjusted with 25% ammonium hydroxide), 100% acetonitrile, and 100% water. Ammonium bicarbonate solution was kept at 10% throughout the run, resulting in a steady 20 mmol per liter concentration. Metabolites were analyzed using a mass spectrometer with a heated electrospray ionization source using polarity switching and the following settings: resolution of 70,000 at m/z of 200; spray voltages of 3,400 V for positive and 3,000 V for negative mode; sheath gas of 28 arbitrary units (AU) and auxiliary gas of 8 AU; vaporizer temperature of 280°C; and ion transfer tube temperature of 300°C. The instrument was controlled using Xcalibur 4.1.31.9 software (Thermo Scientific). Metabolite peaks were confirmed using commercial standards (Sigma-Aldrich). Data quality was monitored throughout the run using an in-house quality control cell line extracted similarly to other samples. After final peak integration with TraceFinder 4.1 SP2 software (Thermo Scientific), peak area data were exported as Excel files. The absolute peak area of a metabolite of interest was normalized to the sum of the absolute peak areas of all metabolites in the same sample. See Table S4 for metabolomics raw data and calculations.

Data analysis

The data from the different software employed were collected in tables and analyzed using R scripts. For multiple comparisons, we used ANOVA coupled with a post hoc Tukey test. Data are represented as the mean ± SEM.

Results

Clinical characteristics of individuals homozygous for the *ASL* variant c.1153C>T

Two unrelated individuals with homozygous *ASL* c.1153C>T variant donated skin biopsies for fibroblast culture.

Person 1 (Table 1) was diagnosed by newborn screening while having hyperammonemia at the time of the diagnosis with relatively mild symptoms. The diagnosis was based on elevated ASA in plasma and urine and was confirmed by genetic testing. Treatment was initiated at 8 days of age, and hyperammonemia resolved rapidly. The individual is on a protein-restricted diet, arginine supplementation, and nitrogen-scavenging medication. After the diagnosis, the person has had one mild hyperammonemic episode and has a developmental delay. The skin biopsy was taken at 18 months of age.

Person 2 (Table 1) was diagnosed at 10 months of age when presenting hyperammonemic symptoms: lethargy and vomiting. Before that, the person showed signs of motor developmental delay. The diagnosis was based on elevated ASA excretion in urine, and a typical plasma amino acid profile of high citrulline and low arginine and was confirmed by ASL enzyme activity measurement in erythrocytes. The genetic testing was done later. The individual has been on a protein-restricted diet and arginine supplementation since the diagnosis, and nitrogen scavengers have been added to the therapy. The person has had several mild to moderate hyperammonemic episodes and has an intellectual disability. The skin biopsy was taken at 30 years of age.

Generation of hepatocyte-like cells from edited and not edited proband-derived hiPSCs to study ASLD

To generate a disease-relevant cell type, we applied a stem cell approach. We edited the pathogenic variant and simultaneously reprogrammed fibroblast derived from two individuals homozygous for the *ASL* variant into hiPSC by electroporating the ABEmax[33] mRNA, a single-guide RNA, and plasmids expressing classic reprogramming factors as previously described.[30] The average on-target A-T to G-C base editing efficiency reached 30% in this reaction, as the targeted base is in the suboptimal ninth position in the editing window.[20] The subsequent experiments were performed using two edited hiPSC lines (*ASL* c.1153 C/C)

Table 1. Clinical characteristics of individuals homozygous for the *ASL* variant c.1153C>T

	Type of diagnosis	Age at diagnosis	Confirmation of diagnosis		ASA (umol/l), not detected normally	Citrulline (umol/l), normal <50	Arginine (umol/l), normal >10	Glutamine (umol/l), normal <800	Ammonium (umol/l), normal <100 in newborns, <50 later	ALT (U/l), normal <50
Person 1	screening	8 days	sequencing	laboratory tests at diagnosis	626	397	23	1,442	314	37
				laboratory tests at follow up	219 (75–446)	175 (84–260)	–	–	46 (10–140)	73 (22–195)
Person 2	symptomatic	10 months	enzyme activity test in erythrocytes: 0.18 μmol/h/g Hb (reference range in the laboratory 5–8 μmol/h/g Hb)	laboratory tests at diagnosis	N/A (high in urine)	149	21	896	190	621
				laboratory tests at follow up	194 (103–291)	214 (97–356)	–	–	61 (39–126)	71 (16–305)

Table presenting clinical data of the two individuals. Plasma amino acids, ammonium, and alanine aminotransferase (ALT) were analyzed in Helsinki University Hospital Laboratory HUSLAB. The reference ranges for amino acids vary according to age; the reference values presented are the rounded mean. The follow-up data from 10 recent years are presented as the mean, and in between parentheses, the lowest and the highest values.

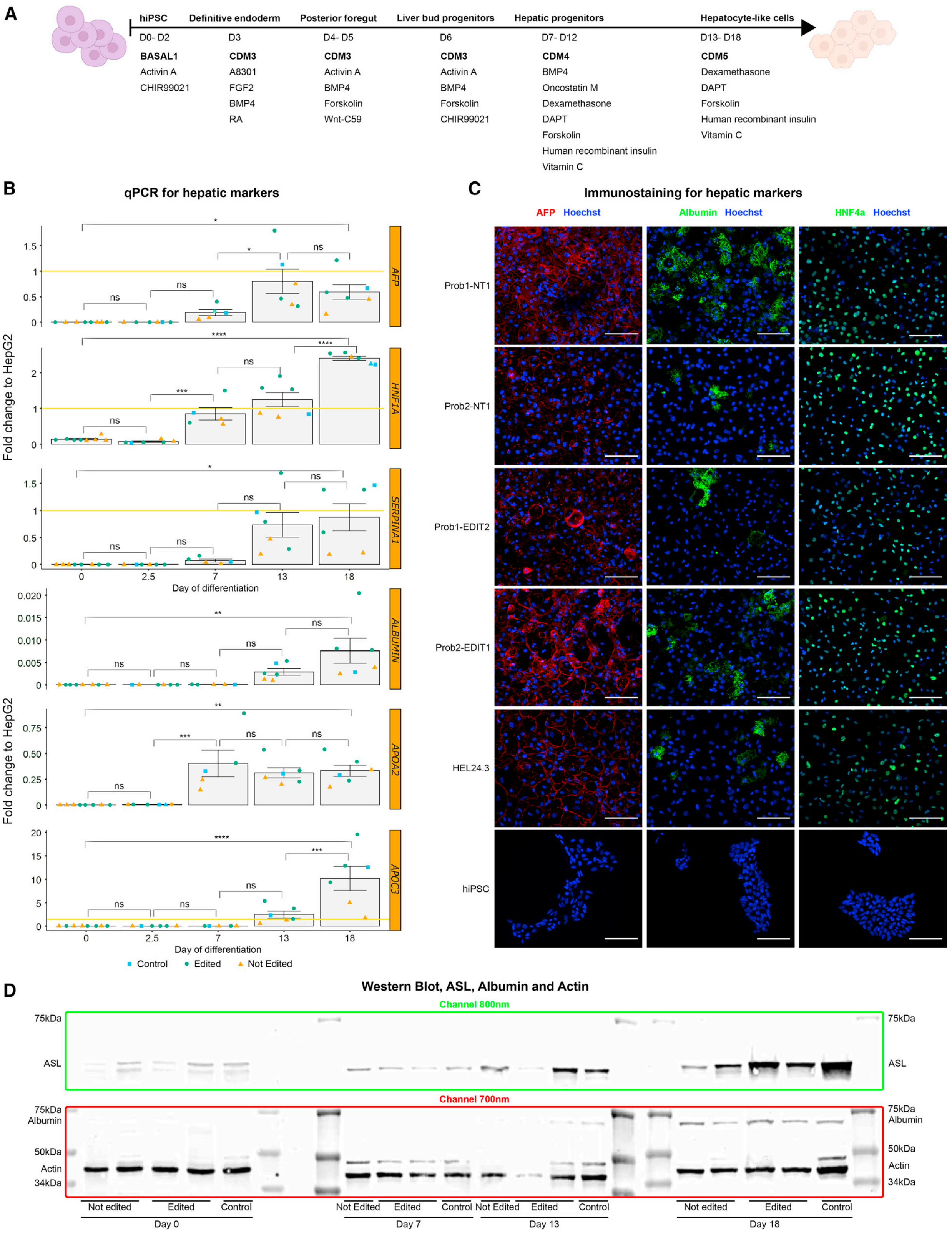

Figure 1. hiPSC differentiation into hepatocyte-like cells

(A) Timeline for the 18-day differentiation protocol. The basic media employed for each stage is depicted in bold letters (BASAL1, CDM3, CDM4, and CDM5). The supplements incorporated in each stage are listed below the basic media.

(B) mRNA levels of essential hepatocyte marker genes. Representative mRNA samples from different individuals, differentiation batches, stages (day 0, 2.5, 7, 13, and 18), and with different genotypes (control = HEL24.3, edited, and not edited) were analyzed by qPCR. The mRNA levels are expressed in fold change and normalized to the HepG2 commercial hepatocarcinoma cell line (illustrated with a yellow line at the fold change 1 on the y axis when the scale allows it). Each point represents an independent hiPSC line, which we consider a

(legend continued on next page)

and two non-edited hiPSC lines (*ASL* c.1153 T/T) from each individual. The quality controls demonstrated pluripotency (Figure S1), genomic integrity (Figure S2), and the absence of off-target effects (Table S1) for all cell lines.

To model ASLD *in vitro*, we differentiated the hiPSC lines toward hepatocyte-like cells, a cell type with an active urea cycle, following a previously described protocol,[31] with some modifications (Figure 1A). On days 0, 2.5, 7, 13, and 18, we assessed the expression of different hepatic markers through qPCR (Figure 1B). The mRNA levels of the hepatocyte markers *AFP*, *HNF1α*, *SERPINA1*, *ALB*, *APOA2*, and *APOC3*[34–36] increased significantly along the time points. On day 18, most of the markers presented a similar or higher expression in the hiPSC-derived hepatocyte-like cells compared to that of the HepG2 hepatocellular carcinoma commercial line. Similarly, the immunocytochemistry imaging indicated robust levels of AFP, HNF4α, and albumin in day 18 hepatocyte-like cells (Figure 1C). Finally, the western blot for samples from day 0 to day 18 corroborated clear albumin accumulation by the end of the protocol and a sharp increase in ASL along the differentiation stages (Figure 1D).

These data show that the genetically edited and non-edited hiPSC lines we generated from individuals homozygous for the *ASL* variant meet high-quality standards for genomic integrity, pluripotency markers, and differentiation potential. Importantly, all the proband-derived hiPSC differentiated equally well to hepatocyte-like cells as the HEL24.3 control hiPSC line.[37]

ABE-mediated editing of the *ASL* c.1153C>T variant reverts the ASA and citrulline accumulation to control levels

Prompted by the successful generation of cell models, we tested for the functional rescue. ASL catalyzes the breakdown of ASA into arginine and fumarate, an essential step in the urea cycle (Figure 2A). Elevated ASA and citrulline in plasma are the primary markers used to diagnose ASLD, followed by genetic testing. We confirmed by western blot (Figure 1D) and qPCR (Figure 2B) that ASL accumulation and *ASL* expression both increase along the hepatocyte differentiation protocol. We then differentiated hepatocyte-like cells from a HEL24.3 control hiPSC line, and two edited and two non-edited independent hiPSC lines per individual. To assess the functionality of the urea cycle in the *ASL*-edited and non-edited hepatocyte-like cells, we performed metabolomics analysis of cell-lysate and media samples using LC-MS metabolomics. The sum of the absolute area of all detected metabolites in each sample did not show significant differences between the different cell lines (Figures 2C and 2D), which suggests that our protocol is robust and does not generate artificial differences along the differentiation and sampling. The edited hepatocyte-like cells (*ASL* c.1153 C/C) showed ASA and citrulline levels similar to the control while the non-edited cells (*ASL* c.1153 T/T) presented significantly higher levels of ASA in the cell-lysate and citrulline in the cell-lysate and media (Figures 2E–2H). Together, this demonstrates the relevance of these hiPSC-derived hepatocyte-like cells for disease modeling of ASLD. Importantly, in the edited hepatocyte-like cells (*ASL* c.1153 C/C) ASA and citrulline levels were restored and indistinguishable compared to the control cell line. As our culture media was supplemented with arginine, we did not see major changes in this metabolite or the ones downstream in the urea cycle (Figure S3; Table S4).

Taken together, these results show that, in hepatocyte-like cells, editing the variant *ASL* c.1153C>T restores the urea cycle, preventing the accumulation of ASA and citrulline, metabolites upstream of the affected enzyme. This is an important observation that supports base editing as an effective curative therapy for ASLD.

LNP-mediated *ASL* editing and metabolic phenotype normalization in ASLD fibroblasts

After confirming that the ABE allows us to efficiently edit the *ASL* c.1153C>T variant thus restoring the ASA and citrulline levels, we sought to design a system compatible with *in vivo* delivery. This is a key step in any gene-editing therapy, as the CRISPR tools need to reach the target organ, in this case, the liver, and edit the genome of a sufficient number of cells. Electroporation is not an option for *in vivo* delivery, therefore, we decided to develop a proof-of-concept LNPs approach. As the ABEmax was not efficient when delivered by LNPs (Figure 3C), we employed a more efficient version of the ABE, the ABE8e, which presents a more processive deaminase component.[26] We designed an mRNA cassette containing the ABE8e coding sequence flanked by the untranslated 5′ and 3′UTRs from the *Xenopus beta-globin* gene.[38] These modifications plus a 5′ m7G(5′)ppp(5′)G and a synthetic polyA tail, increase mRNA stability, translation efficiency, and lower immunogenicity.[39–41] LNPs represent an optimal delivery tool due to their high efficiency, safety profile, and industrial scalability potential demonstrated in the COVID-19 mRNA vaccine production and administration. Commercial FDA-approved LNP formulations consist of different combinations of lipids, and we

biological replicate. Data are represented as the mean ± SEM. Statistical significance based on Tukey test; $p > 0.05$ (ns, not significant), $p < 0.05$ (*), $p < 0.01$ (**), $p < 0.001$ (***), $p < 0.0001$ (****).
(C) Representative immunocytochemistry images of day 18 hepatocyte-like cells. Hoechst is depicted in blue, AFP in red, HNF4α, and albumin in green. All the images were acquired and processed with the same settings to allow comparison. The white bar represents 100 μm.
(D) Western blot for ASL, albumin, and actin. Representative protein samples from different individuals, stages (day 0, 2.5, 7, 13, and 18), and with different genotypes (control = HEL24.3, edited, and not edited) were imaged at the same time and using fluorescent antibodies for western blot.

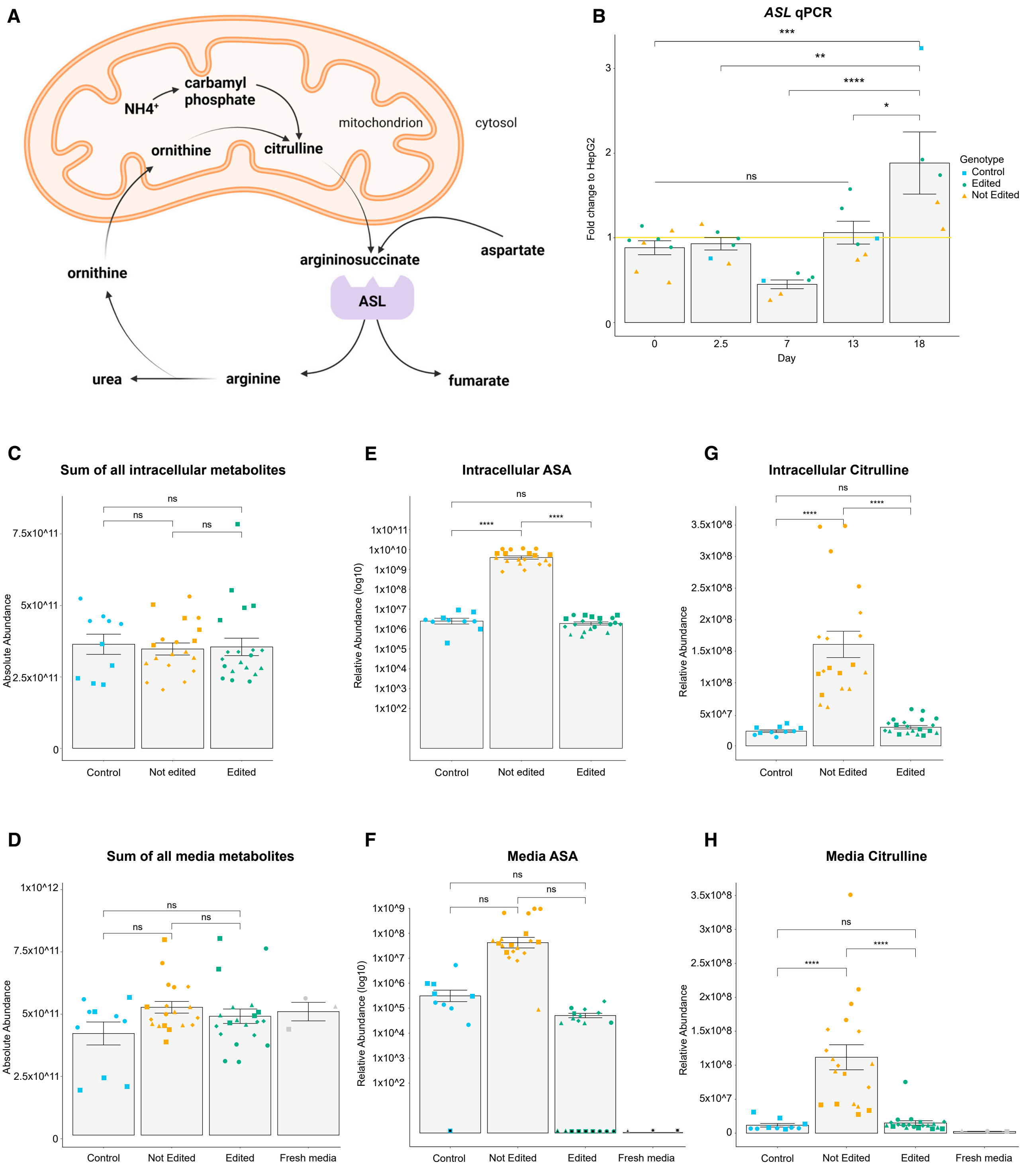

Figure 2. Rescue of the metabolic phenotype after ABE-mediated edition of the ASLD c.1153C>T variant
(A) Urea cycle diagram.
(B) mRNA levels of *ASL*. Representative mRNA samples from different individuals, differentiation batches, stages (day 0, 7, 13, and 18), and with different genotypes (control = HEL24.3, edited, and not edited) were analyzed by qPCR. The mRNA levels are expressed in fold change and normalized to the HepG2 commercial hepatocarcinoma cell line (illustrated with a yellow line at the fold change 1 on the y axis). Each point represents an independent hiPSC line, which we consider a biological replicate. Data are represented as the mean ± SEM. Statistical significance based on Tukey test; $p > 0.05$ (ns, not significant), $p < 0.05$ (*), $p < 0.01$ (**), $p < 0.001$ (***), $p < 0.0001$ (****).
(C and D) The sum of the absolute abundance of all the metabolites detected by LC-MS in the cell lysate (C) and the media (D). Each shape represents independent differentiation batches (circle, square, diamond, triangle). We employed day-18 hiPSC-derived hepatocyte-like cells from two different individuals. We analyzed two independently edited hiPSC lines per proband (four biological replicates), two not edited independent hiPSC lines per proband (four biological replicates), and HEL24.3 as a control (two biological replicates). We processed five technical replicates of each sample in the LC-MS. The sum of the absolute abundance of all the metabolites in each sample

(legend continued on next page)

benchmarked three different ones: mRNA-1273 (Moderna), BNT162b2 (Pfizer-BioNTech), and ALN-18328 (Onpattro/patisiran),[27–29] to determine their efficiency at editing the variant *ASL* c.1153C>T. We produced these LNPs to encapsulate and deliver our optimized ABE8e mRNA and the sgRNA ASL_1153 targeting the variant (Figure 3A). Our first goal was to achieve a dose-dependent A-to-G editing efficiency and to find the lowest dose with the highest effect. We treated primary fibroblasts from the two individuals homozygous for the *ASL* variant in duplicates using different doses, defined as the final amount of RNA computing the mRNA and the sgRNA mass. The on-target A-to-G editing efficiency greatly increased from the LNP ABE8e doses of 17–85 ng but remained at a similar level for higher doses, even after 5,100 ng (Figure 3B). All three formulations exhibited a dose-dependent pattern, but mRNA-1273 and BNT162b2 performed considerably better than ALN-18328, with editing efficiencies surpassing 90% in bulk fibroblast populations (Figure 3C).

The ABE preferentially targets bases 4–8 within the locus targeted by the sgRNA,[43] which represents the editing window (Figure 3A). The ASLD variant that we aimed to edit is located in position 9 of the protospacer on the sgRNA ASL_1153, but this locus presents other adenines (called bystanders) in positions 11 and 12. Using Sanger sequencing, we did not find any editing above the 5% detection threshold for adenine 12 in any of the samples. However, we did detect A-to-G editing for the bystander adenine 11 (Figure 3C), but in all cases, the on-target was higher than the bystander editing. The unwanted change of adenine 11 into guanine resulted in an amino acid change from the uncharged, nonpolar, and hydrophobic phenylalanine to an uncharged, polar, and hydrophilic serine. This amino acid change may affect the ASL structure or activity, as it is located near the active site of the enzyme.[16] The mRNA-1273 85 ng was the lowest dose that resulted in a high on-target A-to-G editing efficiency (86.8% ± 2.7 mean ± SEM) with a relatively low bystander effect (30.5% ± 9.7 mean ± SEM), and no off-target effects (Table S1).

To explore alternatives to lower the bystander effect, we tested a second sgRNA (ASL_1153_II) consisting of a 20 bases protospacer that is shifted one base toward the 5′ of the DNA strand (Figure S4). The protospacer adjacent motif (PAM) for this new sgRNA is TGG, and the target *ASL* variant lies at position 10 instead of position 9 as described for the sgRNA ASL_1153. By shifting the protospacer and, therefore, the editing window, the main bystander (A11 on the previous sgRNA) is now in position 12, which would lower the A-to-G editing efficiency for this adenine. Testing this alternative sgRNA in primary fibroblasts yielded no bystanders, which is positive for a clinical setting but also a much lower on-target editing efficiency (Figure S4). Considering the superior on-target editing efficiency of the initial sgRNA ASL_1153, we opted to continue with this guide for further analysis.

To better quantify the allele frequency and diversity after gene editing, we resampled the primary fibroblast populations previously treated with three different doses (17, 85, and 170 ng RNA) of the LNP formulations mRNA-1273 and BNT162b2 carrying the ABE8e mRNA cassette and the sgRNA ASL_1153. We analyzed the samples by amplicon long-read sequencing technology from Oxford Nanopore Technologies (ONT) and estimated the allele frequency using the software CRISPResso2.[44] We then computed the allele frequency into three categories: precisely edited alleles, those that contained any bystanders or indels, and those that remained unchanged (Figure S5). These data indicate that the editing efficiency, in general, was high even at low LNP doses such as 85 ng and that the frequency of bystanders increases rapidly at higher concentrations such as 170 ng, which is particularly clear in the mRNA-1273 ABE8e treatment.

To assess the toxicity of the different LNP ABE8e formulations, we treated primary fibroblasts from the two individuals homozygous for the *ASL* variant in duplicates using a null (not treated), a minimum (17 ng), a medium (850 ng), and a high LNP ABE8e dose (5,100 ng). During the 70 h follow up, we monitored the number of dead cells and the confluency, which did not significantly differ between any of the treatments and the non-treated samples (Figures 3D, 3E, and S5), indicating the safety of the treatment.

To analyze in detail the off-target activity of the LNP-delivered ABE8e, we selected for each formulation (mRNA-1273 and BNT162b2) the two fibroblast populations with the highest on-target editing efficiency. We then generated a list of 11 putative off-target sites using three different prediction software, Benchling, CRISPOR, and IDT (Table S2); amplified through PCR each locus; and sequenced the DNA samples through ONT. We aligned the raw reads to the reference genome using CRISPResso2 and compared the profile with a non-treated sample. We did not see any difference in the insertion and deletion quantification, but we could detect that the off-target 8 presented substantial A>G conversion (Figure S6). This site lies on the *ASL* pseudogene *ASLP1* (RefSeq: NG_002637). The 4,791 bp long *ASLP1* showed an average sequence identity of 85% to *ASL* after alignment with the

was employed as a normalization to calculate the relative abundance of individual metabolites. Data are represented as the mean ± SEM. Statistical significance based on Tukey test; $p > 0.05$ (ns, not significant), $p < 0.05$ (*), $p < 0.01$ (**), $p < 0.001$ (***), $p < 0.0001$ (****).
(E–H) Relative abundance of intracellular and media ASA (E and F) and citrulline (G and H) detected by LC-MS in the same samples described in the previous graph. Each shape represents independent differentiation batches (circle, square, diamond, triangle). As expected, in some of the control and edited samples, the ASA levels were below the detection limit of the mass spectrometer. We did not consider these values for the graph bar or the statistical analysis, but we illustrated these cases with a shape containing a black center. Relative abundance is the absolute abundance value normalized to the sum of all metabolites. Data are represented as the mean ± SEM. Statistical significance based on Tukey test; $p > 0.05$ (ns, not significant), $p < 0.05$ (*), $p < 0.01$ (**), $p < 0.001$ (***), $p < 0.0001$ (****).

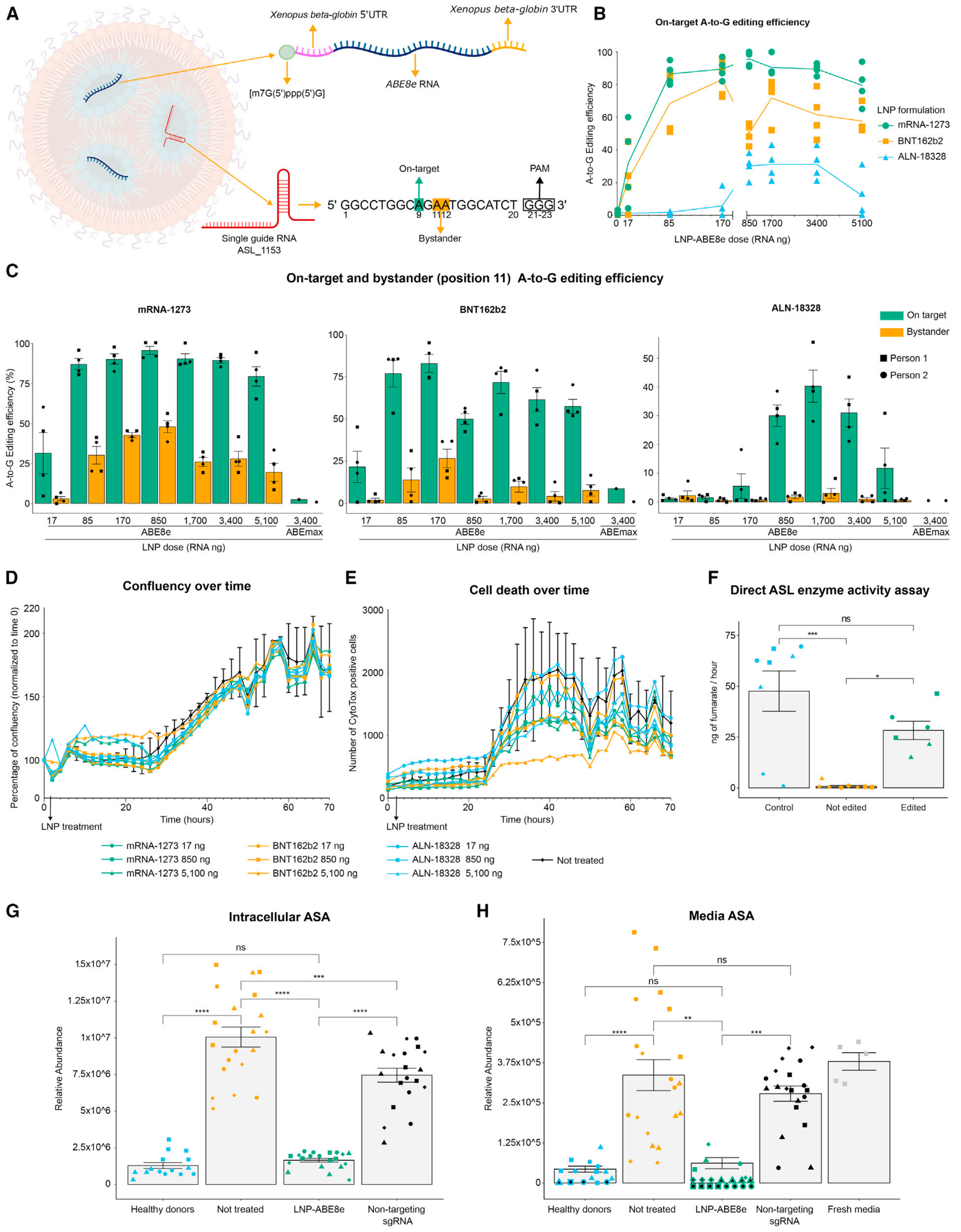

Figure 3. Editing efficiency, toxicity profile, and rescue of the metabolic phenotype in primary fibroblasts after lipid nanoparticles ABE8e treatment

(A) Diagram of the lipid nanoparticle contents: an sgRNA targeting the *ASL* c.1153C>T variant plus an RNA cassette for ABE8e expression. The spacer section of the sgRNA, which targets the DNA, is written from base 1 to base 20. The PAM, not included in the sgRNA oligo, corresponds to bases 21–23 (GGG).

(B) On target A-to-G editing efficiency. We employed primary fibroblasts from two different individuals. We independently treated these fibroblasts in duplicates (four biological replicates) with eight different doses (0–5,100 ng RNA) of three types of lipid nanoparticle ABE8e

(legend continued on next page)

National Center for Biotechnology Information (NCBI) Nucleotide BLAST tool, and it correlates with an immunoglobulin-lambda-like mRNA.[45]

To assess the effect of the gene editing, we measured the ASL enzyme activity in a direct assay from edited, not edited, and control fibroblast cell lysates. Importantly, the treatment with the LNP ABE8e targeting the *ASL* variant significantly increased the enzyme activity in comparison to the non-treated populations, restoring the null activity to around 59% of the healthy donor levels (Figure 3F). This indicated the high potential of LNP-delivered ABE to restore ASL enzyme activity.

Finally, we analyzed the metabolic phenotype following the mRNA-1273 85 ng LNP ABE8e treatment by assessing the intracellular and media levels of ASA and other urea cycle metabolites. We compared fibroblasts derived from three healthy donors against two independently treated populations (edited), two not treated, and two treated with an sgRNA targeting a non-related gene (non-targeting sgRNA) per individual. As expected, the non-targeting-sgRNA and the non-treated populations showed high ASA levels. Importantly, we observed a significant drop in the ASA levels in the cell lysate and the media of the fibroblasts treated with lipid nanoparticle ABE8e targeting the *ASL* variant, reaching the levels of the healthy donors (Figures 3G and 3H).

These results highlight the potential of the LNP-delivered ABE as a method to edit the Finnish founder variant *ASL* c.1153C>T in primary cells *in vitro*. The data show a highly efficient and dose-dependent editing of the variant and a strong phenotypic restoration in the ASA levels and an increase in ASL activity, which suggests recovery of the urea cycle.

Discussion

ASLD presents with symptoms along a continuum of severity, often fatal in the neonatal period. The individuals homozygous for the *ASL* variant c.1153C>T are on the severe end of the disease spectrum.[6,46] Newborn screening enables early diagnosis and improves the initial management of ammonia levels, but in the long-term, the frequency of hyperammonemia episodes does not decrease, even in people with an early diagnosis,[47] and neurological complications still develop.[46] Given the possibility of early diagnosis but with yet unsatisfying treatment outcomes, new therapies are highly needed.[46] We show here that base editors can effectively edit *ASL* c.1153C>T variant and enzyme activity, presenting a proof-of-principle of a gene editing approach for treating ASLD.

Rapidly developing gene editing techniques enable the correction of genetic diseases, which expands the therapeutic options and broadens the field of clinical genetics. Certain medical conditions are especially promising for therapeutic interventions; for example, symptoms and signs caused by a systemic toxic metabolite are a more straightforward target for treatment than those caused by a structural defect in cells. Before any therapeutic development, the functional consequences of the disease variant and the effects of the gene editing require careful evaluation.

(mRNA-1273, BNT162b2, and ALN-18328). One week after the treatment, we estimated the on-target A-to-G editing efficiency by analyzing the Sanger sequence data through EditR.[42] The solid lines represent the mean of each treatment, and each data point is individually represented.

(C) Sequencing data from the experiment in point B is shown here in detail, considering the on-target (green) and bystander (orange) A-to-G editing efficiency. We found bystander editing just in the adenine position 11 but not in position 12. Data are represented as the mean ± SEM. A mid-high dose of ABEmax with no replicates was added as a comparison showing the editing efficiency of the previous generation of ABE.

(D and E) We independently treated primary fibroblasts from two different individuals in duplicates (four biological replicates) with three different doses (17, 850, and 5,100 ng RNA) of three types of lipid nanoparticle ABE8e (mRNA-1273, BNT162b2, and ALN-18328). We followed the fibroblast populations for 70 h, taking pictures in the Incucyte every 2 h. The lipid nanoparticle ABE8e treatment was applied at the 2 h time point. We assessed the confluency (D) and the number of dead cells (E) estimated by the CytoTox dye. The solid lines represent the mean of each treatment. For simplicity, the error bars (SD) are shown only for the non-treated samples (in black). For more detailed data, please check Figure S5.

(F) Direct ASL enzyme activity in fibroblast cell lysates. We employed cell lysates from two fibroblast populations derived from each of the two healthy donors (control, four biological replicates), three ASLD fibroblast populations independently treated with ABE8e mRNA-1273 LNPs targeting the *ASL* variant (edited, three biological replicates), and two not-treated fibroblast populations per each of the two individuals (not edited, four biological replicates). Each sample was processed in two technical replicates. Within each treatment, the data points illustrated with the same shape represent a technical replicate of the enzyme activity assay using the same fibroblast lysate. For the edited group, the triangle and the circle correspond to a lipid nanoparticle dose of 85 ng of RNA, whereas the square corresponds to a dose of 1,700 ng of RNA. Data are represented as the mean ± SEM. Statistical significance based on Tukey test; $p > 0.05$ (ns, not significant), $p < 0.05$ (*), $p < 0.01$ (**), $p < 0.001$ (***), $p < 0.0001$ (****).

(G and H) Relative abundance of ASA in the cell lysate (G) and the media (H) detected by LC-MS. We independently treated primary fibroblasts from two different individuals in duplicates (four biological replicates) with 85 ng RNA of the mRNA-1273 lipid nanoparticle ABE8e plus the variant-targeting sgRNA (LNP-ABE8e), or with vehicle (not treated), or with mRNA-1273 lipid nanoparticle ABE8e containing the sgRNA site_16 targeting an unrelated locus (non-targeting sgRNA). As a control, we used fibroblasts coming from three healthy donors of different genders and ages (healthy donors, three biological replicates). Two weeks after the treatment, we analyzed the metabolite content of each condition, processing five technical replicates of each sample in the LC-MS. As expected, in some of the control and edited samples the ASA levels were below the detection limit of the mass spectrometer. We did not consider these values for the graph bar or the statistical analysis, but we illustrated these cases with a shape containing a black center. Relative abundance is the absolute abundance value normalized to the sum of all metabolites. Data are represented as the mean ± SEM. Statistical significance based on Tukey test; $p > 0.05$ (ns, not significant), $p < 0.05$ (*), $p < 0.01$ (**), $p < 0.001$ (***), $p < 0.0001$ (****).

Our study reports a proband-derived hiPSC-based model for the urea cycle dysfunction in ASLD. Our hepatocyte-like cells display typical hepatocyte markers and show an upregulation of *ASL* expression, confirming their hepatic identity and suitability as an ASLD model. The level of *ASL* mRNA expression or protein accumulation did not differ between edited and non-edited primary cells, consistent with previous findings of the c.1153C>T variant.[15] The differentiation protocol seemed to yield a rather heterogeneous cell population, with an uneven distribution of albumin and HNF4α in individual cells. Still, the *in vitro* model successfully replicated key metabolic features observed in ASLD, including ASA accumulation and increased citrulline levels.

In healthy individuals, ASA is not detected in plasma or urine, as intracellularly produced ASA is immediately cleaved to arginine and fumarate. The individuals in this study presented a high concentration of ASA in plasma at diagnosis, and they continued to show elevated levels during the follow-up despite the protein-restricted diet treatment. They also had increased plasma concentration of citrulline, the upstream metabolite in the urea cycle. We replicated these findings in hepatocyte-like cells, showing the abundance of ASA and citrulline in the cell lysates as well as in the cell culture media from c.1153T/T primary cells. Furthermore, we found ASA and citrulline levels similar to those of healthy controls in the isogenic c.1153C/C primary cells corrected by base editing, proving restoration of urea cycle function.

To generate a CRISPR gene editing approach with potential for *in vivo* applications, we encapsulated the ABE into LNPs. We compared three FDA-approved LNP formulations[27–29] in fibroblasts aiming to edit the *ASL* c.1153C>T variant. This method yielded a high editing efficiency (up to 100%) in a dose-dependent manner. In addition to on-target editing, we also found, with a lower frequency, an unwanted but expected bystander editing change. The bystander edit may negatively affect the ASL structure or function. Nevertheless, the treatment in fibroblasts was effective: it reduced ASA levels to those of the healthy donors and restored the ASL enzyme activity. To evaluate the potential of this approach as a possible therapy, the optimal level of gene editing should be considered. Heterozygous *ASL* c.1153C>T individuals are asymptomatic, suggesting that not all cells or alleles need to be edited. Restoring the ASL activity to 10% of the normal range may protect from severe disease while surpassing the 25% could prevent cognitive impairment.[15] Interestingly, in *ASL* hypomorphic mouse model,[13] a high dose of the liver-targeting adeno-associated virus serotype 8 (AAV8) expressing human codon-optimized *ASL,* recovered up to 25% of the enzyme's activity, restoring the plasma ASA and citrulline levels, and increasing survival and weight gain.[48] Achieving similar enzyme activities through gene editing in ASL-deficient individuals could restore a healthy metabolic profile. In our study, the ABE lipid nanoparticle treatment was not cytotoxic and rescued an average of 59% of the ASL enzyme activity in the primary fibroblasts, while the non-treated lines showed very low ASL activity, consistent with the previous reports of this variant.[15,17,18] Additionally, the ASL activity levels in control cell lines in our experiment were similar to those previously reported.[17] Moreover, the ASA concentration significantly decreased in cell lysates and culture media of edited fibroblast indicating that genetically edited cells could clear ASA from their cytoplasm and their environment.

In clinical trials, LNPs for gene editing have successfully targeted the liver.[49,50] Our results suggest that LNPs are a highly promising tool for the editing of the *ASL* variant in the liver. Such a treatment would restore the urea cycle, protect from hyperammonemia, and, importantly, clear ASA from circulation, preventing the need for liver transplantation, which represents an effective but highly invasive treatment to reduce ASA blood levels.[51] However, in contrast to transplantation, a liver-targeted gene editing therapy would not require lifelong immunosuppression. In addition to the neurotoxic effects of ammonia, ASA has been suggested to be toxic,[2,8] as demonstrated by the correlation of higher ASA levels to higher transaminases.[3,52] This, in turn, indicates a risk for liver fibrosis, a known complication of ASLD.[53] ASA is also detected in high concentrations in the cerebrospinal fluid of ASL-deficient individuals[54] and can be metabolized to neurotoxic guanidinoacetate.[2,11,12] Therefore, editing this variant with the ABE in the central nervous system could have a significant impact on preventing neurological complications, as only editing the variant in hepatocytes may not be enough to tackle the neuronal presentation of the disease. This is technically possible, and a mouse study has reported successful gene editing in the brain.[55] Furthermore, the ASL enzyme, in addition to its role in the urea cycle, produces and channels arginine for NO production, contributing to blood pressure regulation.[2] Editing the ASLD variant in vascular smooth muscle cells or endothelial cells might be technically possible, as these cell types have previously been edited with different CRISPR systems.[56–59]

Gene therapy options are rapidly evolving and under active investigation for urea cycle disorders.[60] Our gene editing strategy offers advantages over gene transfer by viral vectors. LNPs present lower immunogenicity compared to viral vectors, circumventing the risk of existing or triggered anti-viral antibodies, and possibly allowing for subsequent applications of the treatment. The edited gene continues to be expressed under endogenous promoters and physiological regulation, avoiding artificial overexpression of AAV or lentivirus. For *ASL*, this could be important, as its expression in the kidney, lung, and spleen increases after inflammatory stimuli in rats.[61,62]

Pre-existing adaptive immune responses to Cas9 protein, the main component of the ABE, have been reported in the general population,[63–66] which could hinder the efficient gene editing of the targeted organ *in vivo*. In addition, mouse studies have reported inflammatory reactions[67]

and a cytotoxic $CD8^+$ T response[68] associated with Cas9 expression after AAV delivery. From a translational perspective, it is essential to monitor the immune response against Cas9 and the delivery tool of choice before and after the treatment, as it is routinely done in gene-editing clinical trials. Another risk to consider is the possibility of off-target effects, unwanted modifications in regions of the genome not targeted by the sgRNA. Our off-target analysis, in agreement with the literature,[26,69] showed that the ABE8e induces A>G conversions in some loci with a sequence similar to that of the sgRNA. Testing the safety profile of this base editing tool in a preclinical study would require genome-wide off-target analysis and an oncogene screening to understand the risks associated with this therapy. An alternative to improve the specificity could be the ABE8e V106W[26] or SuperFi[69] alternatives, with reduced off-target and on-target activity.

Employing RNA instead of DNA improves the safety of gene editing strategies by ensuring the quick degradation of the vector, transient expression of the encoded Cas9 or ABE protein, and reduced risk of vector integration. GMP lipid nanoparticle RNA vaccines demonstrate scalable artificial synthesis without bioreactors or viral vectors, as witnessed during the COVID-19 pandemic. Our lipid nanoparticle delivery approach for the ABE is a proof-of-principle for successful gene editing therapy in urea cycle disorders and paves the way to systemic trials.

In summary, this study provides a significant advancement in modeling ASLD and proposes a potential therapeutic approach using lipid nanoparticle delivered CRISPR base editor to edit the *ASL* c.1153C>T variant. This technology's advantages over viral vectors, coupled with its reported ability to target the liver, are auspicious for addressing the metabolic systemic phenotype of ASLD and improving the health and prognosis of people suffering from this disease.

Data and code availability

The published article includes all datasets generated or analyzed during this study.

Supplemental information

Supplemental information can be found online at https://doi.org/10.1016/j.ajhg.2024.03.004.

Acknowledgments

The help and advice of Hazem Ibrahim, Hossam Montaser, Joonas Sokka, Solja Eurola, Inkeri Soppa, Nadim Kestilä, Jarkko Ustinov, Helene Markkanen, Sonam Gurung, Leena Saikko, and Anni Laitinen significantly enriched this article. Likewise, the guidance and scientific support of Dr. Diego Balboa were essential for critical feedback and troubleshooting.

The work was funded by Helsinki University Hospital Research funds, the Academy of Finland Centre of Excellence on Stem Cell Metabolism, the Foundation for Pediatric Research, the Paulo Foundation, the Magnus Ehrnrooth Foundation, and the Orion Research Foundation.

Author contributions

Conceptualization, S.J., T.K, M.E.H., and K.W.; methodology, S.J., T.K., and E.K.; metabolomics, S.J., J.J., T.K., and E.K.; investigation, S.J. and T.K.; formal analysis, S.J. and T.K.; visualization, S.J. and R.S.M.; writing – original draft, S.J., T.K., and M.E.H.; writing – review & editing, S.J., T.K, M.E.H., R.S.M., E.K., R.L., V.H., A.S., T.O., and K.W.; resources, T.O., K.W., V.H., and M.E.H.; supervision, K.W. and M.E.H.; project administration, S.J. and K.W.; funding acquisition, K.W., T.O., A.S., and M.E.H.

Declaration of interests

The authors declare no competing interests.

Received: August 23, 2023
Accepted: March 6, 2024
Published: April 4, 2024

References

1. Erez, A., Nagamani, S.C.S., and Lee, B. (2011). Argininosuccinate lyase deficiency-argininosuccinic aciduria and beyond. Am. J. Med. Genet. C Semin. Med. Genet. *157C*, 45–53. https://doi.org/10.1002/AJMG.C.30289.
2. Baruteau, J., Diez-Fernandez, C., Lerner, S., Ranucci, G., Gissen, P., Dionisi-Vici, C., Nagamani, S., Erez, A., and Häberle, J. (2019). Argininosuccinic aciduria: Recent pathophysiological insights and therapeutic prospects. J. Inherit. Metab. Dis. *42*, 1147–1161. https://doi.org/10.1002/JIMD.12047.
3. Nagamani, S.C.S., Erez, A., and Lee, B. (2012). Argininosuccinate lyase deficiency. Genet. Med. *14*, 501–507. https://doi.org/10.1038/GIM.2011.1.
4. Dionisi-Vici, C., Rizzo, C., Burlina, A.B., Caruso, U., Sabetta, G., Uziel, G., and Abeni, D. (2002). Inborn errors of metabolism in the Italian pediatric population: A national retrospective survey. J. Pediatr. *140*, 321–327. https://doi.org/10.1067/mpd.2002.122394.
5. Summar, M.L., Koelker, S., Freedenberg, D., Le Mons, C., Haberle, J., Lee, H.S., Kirmse, B.; European Registry and Network for Intoxication Type Metabolic Diseases E-IMD. Electronic address: http://www.e-imd.org/en/index.phtml; and Members of the Urea Cycle Disorders Consortium UCDC. Electronic address: http://rarediseasesnetwork.epi.usf.edu/ucdc/ (2013). The incidence of urea cycle disorders. Mol. Genet. Metab. *110*, 179–180. https://doi.org/10.1016/J.YMGME.2013.07.008.
6. Keskinen, P., Siitonen, A., and Salo, M. (2008). Hereditary urea cycle diseases in Finland. Acta Paediatr. *97*, 1412–1419. https://doi.org/10.1111/J.1651-2227.2008.00923.X.
7. Palekar, A.G., and Mantagos, S. (1981). Human Liver Argininosuccinase Purification and Partial Characterization. J. Biol. Chem. *256*, 9192–9194. https://doi.org/10.1016/S0021-9258(19)52526-1.
8. Diez-Fernandez, C., Hertig, D., Loup, M., Diserens, G., Henry, H., Vermathen, P., Nuoffer, J.M., Häberle, J., and Braissant, O. (2019). Argininosuccinate neurotoxicity and prevention by

creatine in argininosuccinate lyase deficiency: An in vitro study in rat three-dimensional organotypic brain cell cultures. J. Inherit. Metab. Dis. *42*, 1077–1087. https://doi.org/10.1002/jimd.12090.
9. Allan, J.D., Cusworth, D.C., Dent, C.E., and Wilson, V.K. (1958). A DISEASE, PROBABLY HEREDITARY, CHARACTERISED BY SEVERE MENTAL DEFICIENCY AND A CONSTANT GROSS ABNORMALITY OF AMINOACID METABOLISM. Lancet *1*, 182–187. https://doi.org/10.1016/S0140-6736(58)90666-4.
10. Braissant, O., McLin, V.A., and Cudalbu, C. (2013). Ammonia toxicity to the brain. J. Inherit. Metab. Dis. *36*, 595–612. https://doi.org/10.1007/s10545-012-9546-2.
11. Aoyagi, K. (2003). Inhibition of arginine synthesis by urea: a mechanism for arginine deficiency in renal failure which leads to increased hydroxyl radical generation. Mol. Cell. Biochem. *244*, 11–15.
12. D'Hooge, R., Pei, Y.-Q., Manil, J., and De Deyn, P.P. (1992). The uremic quanidino compound guanidinosuccinic acid induces behavioral convulsions and concomitant epileptiform electrocorticographic discharges in mice. Brain Res. *598*, 316–320. https://doi.org/10.1016/0006-8993(92)90200-S.
13. Erez, A., Nagamani, S.C.S., Shchelochkov, O.A., Premkumar, M.H., Campeau, P.M., Chen, Y., Garg, H.K., Li, L., Mian, A., Bertin, T.K., et al. (2011). Requirement of argininosuccinate lyase for systemic nitric oxide production. Nat. Med. *17*, 1619–1626. https://doi.org/10.1038/nm.2544.
14. Hermann, M., Flammer, A., and Lüscher, T.F. (2006). Nitric oxide in hypertension. J. Clin. Hypertens. *8*, 17–29. https://doi.org/10.1111/J.1524-6175.2006.06032.X.
15. Zielonka, M., Garbade, S.F., Gleich, F., Okun, J.G., Nagamani, S.C.S., Gropman, A.L., Hoffmann, G.F., Kölker, S., Posset, R., et al.; Urea Cycle Disorders Consortium UCDC and the European registry and network for Intoxication type Metabolic Diseases E-IMD Consortia Study Group (2020). From genotype to phenotype: Early prediction of disease severity in argininosuccinic aciduria. Hum. Mutat. *41*, 946–960. https://doi.org/10.1002/humu.23983.
16. Balmer, C., Pandey, A.V., Rüfenacht, V., Nuoffer, J.M., Fang, P., Wong, L.J., and Häberle, J. (2014). Mutations and polymorphisms in the human argininosuccinate lyase (ASL) gene. Hum. Mutat. *35*, 27–35. https://doi.org/10.1002/HUMU.22469.
17. Kleijer, W.J., Garritsen, V.H., Linnebank, M., Mooyer, P., Huijmans, J.G.M., Mustonen, A., Simola, K.O.J., Arslan-Kirchner, M., Battini, R., Briones, P., et al. (2002). Clinical, enzymatic, and molecular genetic characterization of a biochemical variant type of argininosuccinic aciduria: Prenatal and postnatal diagnosis in five unrelated families. J. Inherit. Metab. Dis. *25*, 399–410. https://doi.org/10.1023/A:1020108002877.
18. Engel, K., Vuissoz, J.M., Eggimann, S., Groux, M., Berning, C., Hu, L., Klaus, V., Moeslinger, D., Mercimek-Mahmutoglu, S., Stöckler, S., et al. (2012). Bacterial expression of mutant argininosuccinate lyase reveals imperfect correlation of *in - vitro* enzyme activity with clinical phenotype in argininosuccinic aciduria. J. Inherit. Metab. Dis. *35*, 133–140. https://doi.org/10.1007/s10545-011-9357-x.
19. Rees, H.A., and Liu, D.R. (2018). Base editing: precision chemistry on the genome and transcriptome of living cells. Nat. Rev. Genet. *19*, 770–788. https://doi.org/10.1038/s41576-018-0059-1.
20. Gaudelli, N.M., Komor, A.C., Rees, H.A., Packer, M.S., Badran, A.H., Bryson, D.I., and Liu, D.R. (2017). Programmable base editing of A•T to G•C in genomic DNA without DNA cleavage. Nature *551*, 464–471. https://doi.org/10.1038/nature24644.
21. Komor, A.C., Badran, A.H., and Liu, D.R. (2018). Editing the Genome Without Double-Stranded DNA Breaks. ACS Chem. Biol. *13*, 383–388. https://doi.org/10.1021/acschembio.7b00710.
22. Liang, P., Xie, X., Zhi, S., Sun, H., Zhang, X., Chen, Y., Chen, Y., Xiong, Y., Ma, W., Liu, D., et al. (2019). Genome-wide profiling of adenine base editor specificity by EndoV-seq. Nat. Commun. *10*, 67. https://doi.org/10.1038/s41467-018-07988-z.
23. Jin, S., Zong, Y., Gao, Q., Zhu, Z., Wang, Y., Qin, P., Liang, C., Wang, D., Qiu, J.-L., Zhang, F., and Gao, C. (2019). Cytosine, but not adenine, base editors induce genome-wide off-target mutations in rice. Science *364*, 292–295. https://doi.org/10.1126/science.aaw7166.
24. Xu, L., Zhang, C., Li, H., Wang, P., Gao, Y., Mokadam, N.A., Ma, J., Arnold, W.D., and Han, R. (2021). Efficient precise in vivo base editing in adult dystrophic mice. Nat. Commun. *12*, 3719. https://doi.org/10.1038/s41467-021-23996-y.
25. Kim, Y., Hong, S.-A., Yu, J., Eom, J., Jang, K., Yoon, S., Hong, D.H., Seo, D., Lee, S.-N., Woo, J.-S., et al. (2021). Adenine base editing and prime editing of chemically derived hepatic progenitors rescue genetic liver disease. Cell Stem Cell *28*, 1614–1624.e5. https://doi.org/10.1016/j.stem.2021.04.010.
26. Richter, M.F., Zhao, K.T., Eton, E., Lapinaite, A., Newby, G.A., Thuronyi, B.W., Wilson, C., Koblan, L.W., Zeng, J., Bauer, D.E., et al. (2020). Phage-assisted evolution of an adenine base editor with improved Cas domain compatibility and activity. Nat. Biotechnol. *38*, 883–891. https://doi.org/10.1038/S41587-020-0453-Z.
27. Fang, Z., Peng, L., Filler, R., Suzuki, K., McNamara, A., Lin, Q., Renauer, P.A., Yang, L., Menasche, B., Sanchez, A., et al. (2022). Omicron-specific mRNA vaccination alone and as a heterologous booster against SARS-CoV-2. Nat. Commun. *13*, 3250. https://doi.org/10.1038/s41467-022-30878-4.
28. Jayaraman, M., Ansell, S.M., Mui, B.L., Tam, Y.K., Chen, J., Du, X., Butler, D., Eltepu, L., Matsuda, S., Narayanannair, J.K., et al. (2012). Maximizing the Potency of siRNA Lipid Nanoparticles for Hepatic Gene Silencing In Vivo. Angew. Chem. *124*, 8657–8661. https://doi.org/10.1002/ange.201203263.
29. Schoenmaker, L., Witzigmann, D., Kulkarni, J.A., Verbeke, R., Kersten, G., Jiskoot, W., and Crommelin, D.J.A. (2021). mRNA-lipid nanoparticle COVID-19 vaccines: Structure and stability. Int. J. Pharm. *601*, 120586. https://doi.org/10.1016/J.IJPHARM.2021.120586.
30. Jalil, S., Keskinen, T., Maldonado, R., Sokka, J., Trokovic, R., Otonkoski, T., and Wartiovaara, K. (2021). Simultaneous high-efficiency base editing and reprogramming of patient fibroblasts. Stem Cell Rep. *16*, 3064–3075. https://doi.org/10.1016/J.STEMCR.2021.10.017.
31. Ang, L.T., Tan, A.K.Y., Autio, M.I., Goh, S.H., Choo, S.H., Lee, K.L., Tan, J., Pan, B., Lee, J.J.H., Lum, J.J., et al. (2018). A Roadmap for Human Liver Differentiation from Pluripotent Stem Cells. Cell Rep. *22*, 2190–2205. https://doi.org/10.1016/J.CELREP.2018.01.087.
32. Verbeke, R., Lentacker, I., De Smedt, S.C., and Dewitte, H. (2021). The dawn of mRNA vaccines: The COVID-19 case. J. Control. Release *333*, 511–520. https://doi.org/10.1016/j.jconrel.2021.03.043.
33. Koblan, L.W., Doman, J.L., Wilson, C., Levy, J.M., Tay, T., Newby, G.A., Maianti, J.P., Raguram, A., and Liu, D.R.

(2018). Improving cytidine and adenine base editors by expression optimization and ancestral reconstruction. Nat. Biotechnol. *36*, 843–846. https://doi.org/10.1038/nbt.4172.
34. Wesley, B.T., Ross, A.D.B., Muraro, D., Miao, Z., Saxton, S., Tomaz, R.A., Morell, C.M., Ridley, K., Zacharis, E.D., Petrus-Reurer, S., et al. (2022). Single-cell atlas of human liver development reveals pathways directing hepatic cell fates. Nat. Cell Biol. *24*, 1487–1498. https://doi.org/10.1038/S41556-022-00989-7.
35. Lokmane, L., Haumaitre, C., Garcia-Villalba, P., Anselme, I., Schneider-Maunoury, S., and Cereghini, S. (2008). Crucial role of vHNF1 in vertebrate hepatic specification. Development *135*, 2777–2786. https://doi.org/10.1242/dev.023010.
36. Fagerberg, L., Hallström, B.M., Oksvold, P., Kampf, C., Djureinovic, D., Odeberg, J., Habuka, M., Tahmasebpoor, S., Danielsson, A., Edlund, K., et al. (2014). Analysis of the Human Tissue-specific Expression by Genome-wide Integration of Transcriptomics and Antibody-based Proteomics. Mol. Cell. Proteomics *13*, 397–406. https://doi.org/10.1074/mcp.M113.035600.
37. Trokovic, R., Weltner, J., and Otonkoski, T. (2015). Generation of iPSC line HEL24.3 from human neonatal foreskin fibroblasts. Stem Cell Res. *15*, 266–268. https://doi.org/10.1016/j.scr.2015.05.012.
38. Krieg, P.A., and Melton, D.A. (1984). Functional messenger RNAs are produced by SP6 *in vitro* transcription of cloned cDNAs. Nucleic Acids Res. *12*, 7057–7070. https://doi.org/10.1093/nar/12.18.7057.
39. Karikó, K., Buckstein, M., Ni, H., and Weissman, D. (2005). Suppression of RNA Recognition by Toll-like Receptors: The Impact of Nucleoside Modification and the Evolutionary Origin of RNA. Immunity *23*, 165–175. https://doi.org/10.1016/j.immuni.2005.06.008.
40. Mu, X., Greenwald, E., Ahmad, S., and Hur, S. (2018). An origin of the immunogenicity of in vitro transcribed RNA. Nucleic Acids Res. *46*, 5239–5249. https://doi.org/10.1093/nar/gky177.
41. Li, M., Wang, Z., Xie, C., and Xia, X. (2022). Chapter Seven - Advances in mRNA vaccines. In mRNA-Based Therapeutics International Review of Cell and Molecular Biology, F. Aranda, P. Berraondo, and L. Galluzzi, eds. (Academic Press), pp. 295–316. https://doi.org/10.1016/bs.ircmb.2022.04.011.
42. Kluesner, M.G., Nedveck, D.A., Lahr, W.S., Garbe, J.R., Abrahante, J.E., Webber, B.R., and Moriarity, B.S. (2018). EditR: A Method to Quantify Base Editing from Sanger Sequencing. CRISPR J. *1*, 239–250. https://doi.org/10.1089/crispr.2018.0014.
43. Anzalone, A.V., Koblan, L.W., and Liu, D.R. (2020). Genome editing with CRISPR–Cas nucleases, base editors, transposases and prime editors. Nat. Biotechnol. *38*, 824–844. https://doi.org/10.1038/s41587-020-0561-9.
44. Clement, K., Rees, H., Canver, M.C., Gehrke, J.M., Farouni, R., Hsu, J.Y., Cole, M.A., Liu, D.R., Joung, J.K., Bauer, D.E., and Pinello, L. (2019). CRISPResso2 provides accurate and rapid genome editing sequence analysis. Nat. Biotechnol. *37*, 224–226. https://doi.org/10.1038/s41587-019-0032-3.
45. Linnebank, M., Tschiedel, E., Häberle, J., Linnebank, A., Willenbring, H., Kleijer, W.J., and Koch, H.G. (2002). Argininosuccinate lyase (ASL) deficiency: mutation analysis in 27 patients and a completed structure of the human ASL gene. Hum. Genet. *111*, 350–359. https://doi.org/10.1007/S00439-002-0793-4.
46. Baruteau, J., Jameson, E., Morris, A.A., Chakrapani, A., Santra, S., Vijay, S., Kocadag, H., Beesley, C.E., Grunewald, S., Murphy, E., et al. (2017). Expanding the phenotype in argininosuccinic aciduria: need for new therapies. J. Inherit. Metab. Dis. *40*, 357–368. https://doi.org/10.1007/s10545-017-0022-x.
47. Posset, R., Kölker, S., Gleich, F., Okun, J.G., Gropman, A.L., Nagamani, S.C.S., Scharre, S., Probst, J., Walter, M.E., Hoffmann, G.F., et al. (2020). Severity-adjusted evaluation of newborn screening on the metabolic disease course in individuals with cytosolic urea cycle disorders. Mol. Genet. Metab. *131*, 390–397. https://doi.org/10.1016/j.ymgme.2020.10.013.
48. Ashley, S.N., Nordin, J.M.L., Buza, E.L., Greig, J.A., and Wilson, J.M. (2018). Adeno-associated viral gene therapy corrects a mouse model of argininosuccinic aciduria. Mol. Genet. Metab. *125*, 241–250. https://doi.org/10.1016/j.ymgme.2018.08.013.
49. Gillmore, J.D., Gane, E., Taubel, J., Kao, J., Fontana, M., Maitland, M.L., Seitzer, J., O'Connell, D., Walsh, K.R., Wood, K., et al. (2021). CRISPR-Cas9 In Vivo Gene Editing for Transthyretin Amyloidosis. N. Engl. J. Med. *385*, 493–502. https://doi.org/10.1056/NEJMoa2107454.
50. Cullis, P.R., and Hope, M.J. (2017). Lipid Nanoparticle Systems for Enabling Gene Therapies. Mol. Ther. *25*, 1467–1475. https://doi.org/10.1016/j.ymthe.2017.03.013.
51. Marble, M., McGoey, R.R., Mannick, E., Keats, B., Ng, S.S., Deputy, S., Gereighty, H., and Schmidt-Sommerfeld, E. (2008). Living Related Liver Transplant in a Patient With Argininosuccinic Aciduria and Cirrhosis: Metabolic Follow-up. J. Pediatr. Gastroenterol. Nutr. *46*, 453–456. https://doi.org/10.1097/MPG.0b013e3180ca8720.
52. Nagamani, S.C.S., Shchelochkov, O.A., Mullins, M.A., Carter, S., Lanpher, B.C., Sun, Q., Kleppe, S., Erez, A., O'Brian Smith, E., Marini, J.C., et al. (2012). A randomized controlled trial to evaluate the effects of high-dose versus low-dose of arginine therapy on hepatic function tests in argininosuccinic aciduria. Mol. Genet. Metab. *107*, 315–321. https://doi.org/10.1016/j.ymgme.2012.09.016.
53. Mori, T., Nagai, K., Mori, M., Nagao, M., Imamura, M., Iijima, M., and Kobayashi, K. (2002). Progressive Liver Fibrosis in Late-onset Argininosuccinate Lyase Deficiency. Pediatr. Dev. Pathol. *5*, 597–601. https://doi.org/10.1007/s10024-002-0109-7.
54. Tomlinson, S., and Westall, R.G. (1960). Argininosuccinase Activity in Brain Tissue. Nature *188*, 235–236. https://doi.org/10.1038/188235a0.
55. Levy, J.M., Yeh, W.H., Pendse, N., Davis, J.R., Hennessey, E., Butcher, R., Koblan, L.W., Comander, J., Liu, Q., and Liu, D.R. (2020). Cytosine and adenine base editing of the brain, liver, retina, heart and skeletal muscle of mice via adeno-associated viruses. Nat. Biomed. Eng. *4*, 97–110. https://doi.org/10.1038/S41551-019-0501-5.
56. Atri, D.S., Lee-Kim, V.S., Vellarikkal, S.K., Sias-Garcia, O., Yanamandala, M., Schniztler, G.R., and Gupta, R.M. (2021). CRISPR-Cas9 Genome Editing of Primary Human Vascular Cells In Vitro. Curr. Protoc. *1*, e291. https://doi.org/10.1002/cpz1.291.
57. Wang, J., Zhang, L., Wu, G., Wu, J., Zhou, X., Chen, X., Niu, Y., Jiao, Y., Liu, Q., Liang, P., et al. (2024). Correction of a CADASIL point mutation using adenine base editors in hiPSCs and blood vessel organoids. Journal of Genetics and Genomics *51*, 197–207. https://doi.org/10.1016/j.jgg.2023.04.013.

58. Koblan, L.W., Erdos, M.R., Wilson, C., Cabral, W.A., Levy, J.M., Xiong, Z.-M., Tavarez, U.L., Davison, L.M., Gete, Y.G., Mao, X., et al. (2021). In vivo base editing rescues Hutchinson–Gilford progeria syndrome in mice. Nature *589*, 608–614. https://doi.org/10.1038/s41586-020-03086-7.

59. Wu, W., Duan, Y., Ma, G., Zhou, G., Park-Windhol, C., D'Amore, P.A., and Lei, H. (2017). AAV-CRISPR/Cas9–Mediated Depletion of VEGFR2 Blocks Angiogenesis In Vitro. Invest. Ophthalmol. Vis. Sci. *58*, 6082–6090. https://doi.org/10.1167/iovs.17-21902.

60. Duff, C., Alexander, I.E., and Baruteau, J. (2024). Gene therapy for urea cycle defects: An update from historical perspectives to future prospects. J. Inherit. Metab. Dis. *47*, 50–62. https://doi.org/10.1002/jimd.12609.

61. Nagasaki, A., Gotoh, T., Takeya, M., Yu, Y., Takiguchi, M., Matsuzaki, H., Takatsuki, K., and Mori, M. (1996). Coinduction of Nitric Oxide Synthase, Argininosuccinate Synthetase, and Argininosuccinate Lyase in Lipopolysaccharide-treated Rats. J. Biol. Chem. *271*, 2658–2662. https://doi.org/10.1074/jbc.271.5.2658.

62. Hattori, Y., Shimoda, S., and Gross, S.S. (1995). Effect of Lipopolysaccharide Treatment in Vivo on Tissue Expression of Argininosuccinate Synthetase and Argininosuccinate Lyase mRNAs: Relationship to Nitric Oxide Synthase. Biochem. Biophys. Res. Commun. *215*, 148–153. https://doi.org/10.1006/bbrc.1995.2445.

63. Simhadri, V.L., McGill, J., McMahon, S., Wang, J., Jiang, H., and Sauna, Z.E. (2018). Prevalence of Pre-existing Antibodies to CRISPR-Associated Nuclease Cas9 in the USA Population. Mol. Ther. Methods Clin. Dev. *10*, 105–112. https://doi.org/10.1016/j.omtm.2018.06.006.

64. Charlesworth, C.T., Deshpande, P.S., Dever, D.P., Camarena, J., Lemgart, V.T., Cromer, M.K., Vakulskas, C.A., Collingwood, M.A., Zhang, L., Bode, N.M., et al. (2019). Identification of preexisting adaptive immunity to Cas9 proteins in humans. Nat. Med. *25*, 249–254. https://doi.org/10.1038/s41591-018-0326-x.

65. Ferdosi, S.R., Ewaisha, R., Moghadam, F., Krishna, S., Park, J.G., Ebrahimkhani, M.R., Kiani, S., and Anderson, K.S. (2019). Multifunctional CRISPR-Cas9 with engineered immunosilenced human T cell epitopes. Nat. Commun. *10*, 1842. https://doi.org/10.1038/s41467-019-09693-x.

66. Wagner, D.L., Amini, L., Wendering, D.J., Burkhardt, L.-M., Akyüz, L., Reinke, P., Volk, H.-D., and Schmueck-Henneresse, M. (2019). High prevalence of Streptococcus pyogenes Cas9-reactive T cells within the adult human population. Nat. Med. *25*, 242–248. https://doi.org/10.1038/s41591-018-0204-6.

67. Wang, D., Mou, H., Li, S., Li, Y., Hough, S., Tran, K., Li, J., Yin, H., Anderson, D.G., Sontheimer, E.J., et al. (2015). Adenovirus-Mediated Somatic Genome Editing of *Pten* by CRISPR/Cas9 in Mouse Liver in Spite of Cas9-Specific Immune Responses. Hum. Gene Ther. *26*, 432–442. https://doi.org/10.1089/hum.2015.087.

68. Li, A., Tanner, M.R., Lee, C.M., Hurley, A.E., De Giorgi, M., Jarrett, K.E., Davis, T.H., Doerfler, A.M., Bao, G., Beeton, C., and Lagor, W.R. (2020). AAV-CRISPR Gene Editing Is Negated by Pre-existing Immunity to Cas9. Mol. Ther. *28*, 1432–1441. https://doi.org/10.1016/j.ymthe.2020.04.017.

69. Kulcsár, P.I., Tálas, A., Ligeti, Z., Krausz, S.L., and Welker, E. (2022). SuperFi-Cas9 exhibits remarkable fidelity but severely reduced activity yet works effectively with ABE8e. Nat. Commun. *13*, 6858. https://doi.org/10.1038/s41467-022-34527-8.

The American Journal of Human Genetics, Volume *111*

Supplemental information

Genetic and functional correction of argininosuccinate lyase deficiency using CRISPR adenine base editors

Sami Jalil, Timo Keskinen, Juhana Juutila, Rocio Sartori Maldonado, Liliya Euro, Anu Suomalainen, Risto Lapatto, Emilia Kuuluvainen, Ville Hietakangas, Timo Otonkoski, Mervi E. Hyvönen, and Kirmo Wartiovaara

Supplemental Figures and Legends. Figure S1

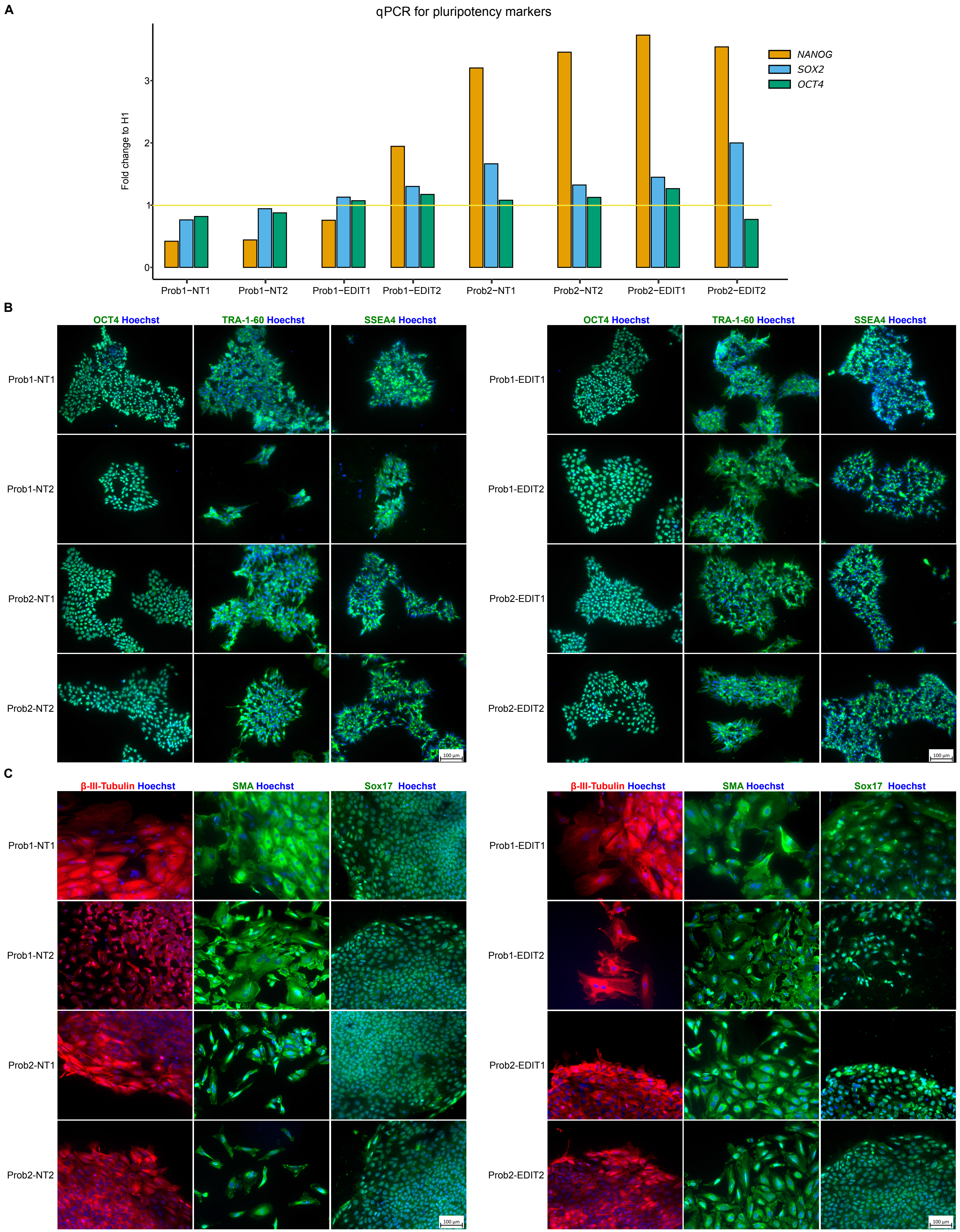

Figure S1: Assessment of the hiPSC lines pluripotency.

(A) We tested the mRNA levels of three essential pluripotency markers (*NANOG*, *SOX2*, and *OCT4*) in two edited hiPSC lines (*ASL* c.1153 C/C) and two non-edited hiPSC lines (*ASL* c.1153 T/T) per proband. These hiPSCs were later employed for hepatocyte differentiation. **(B)** Immunofluorescence staining of the same hiPSC lines illustrating a normal morphology and expression of the pluripotency markers OCT4, TRA-1-60, and SSEA4. Hoechst, in blue, is a nuclear marker. **(C)** Immunofluorescence staining of embryoid bodies derived from the same hiPSC lines. β-III-Tubulin (ectoderm), smooth muscle actin (SMA) (mesoderm), and Sox17 (endoderm). Hoechst, in blue, is a nuclear marker.

Figure S2

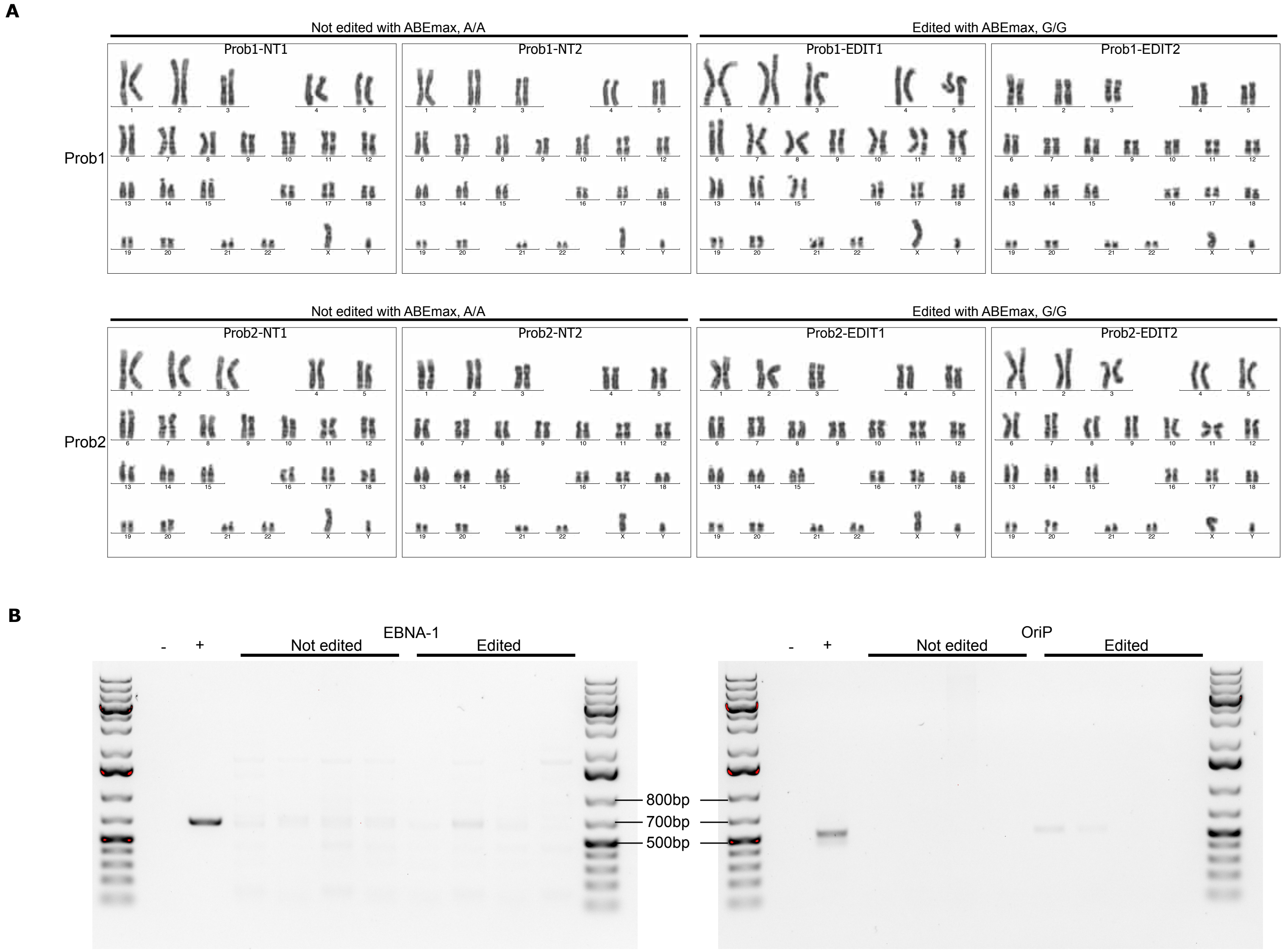

Figure S2: Genomic integrity of the edited and not edited hiPSC monoclonal lines.

(A) Normal karyotype of four hiPSC monoclonal lines reprogrammed from proband fibroblasts in the absence of the ABEmax construct (not edited) and four hiPSC monoclonal lines reprogrammed and simultaneously treated with the ABEmax construct (edited). **(B)** PCR to detect the episomal reprogramming vectors retention in four hiPSC monoclonal lines reprogrammed from proband fibroblasts in the absence of the ABEmax construct (not edited) and four hiPSC monoclonal lines reprogrammed and simultaneously treated with the ABEmax construct (edited). Two different plasmid regions are targeted in these PCRs: EBNA-1 and OriP. Water control (−), positive control (+).

Figure S3

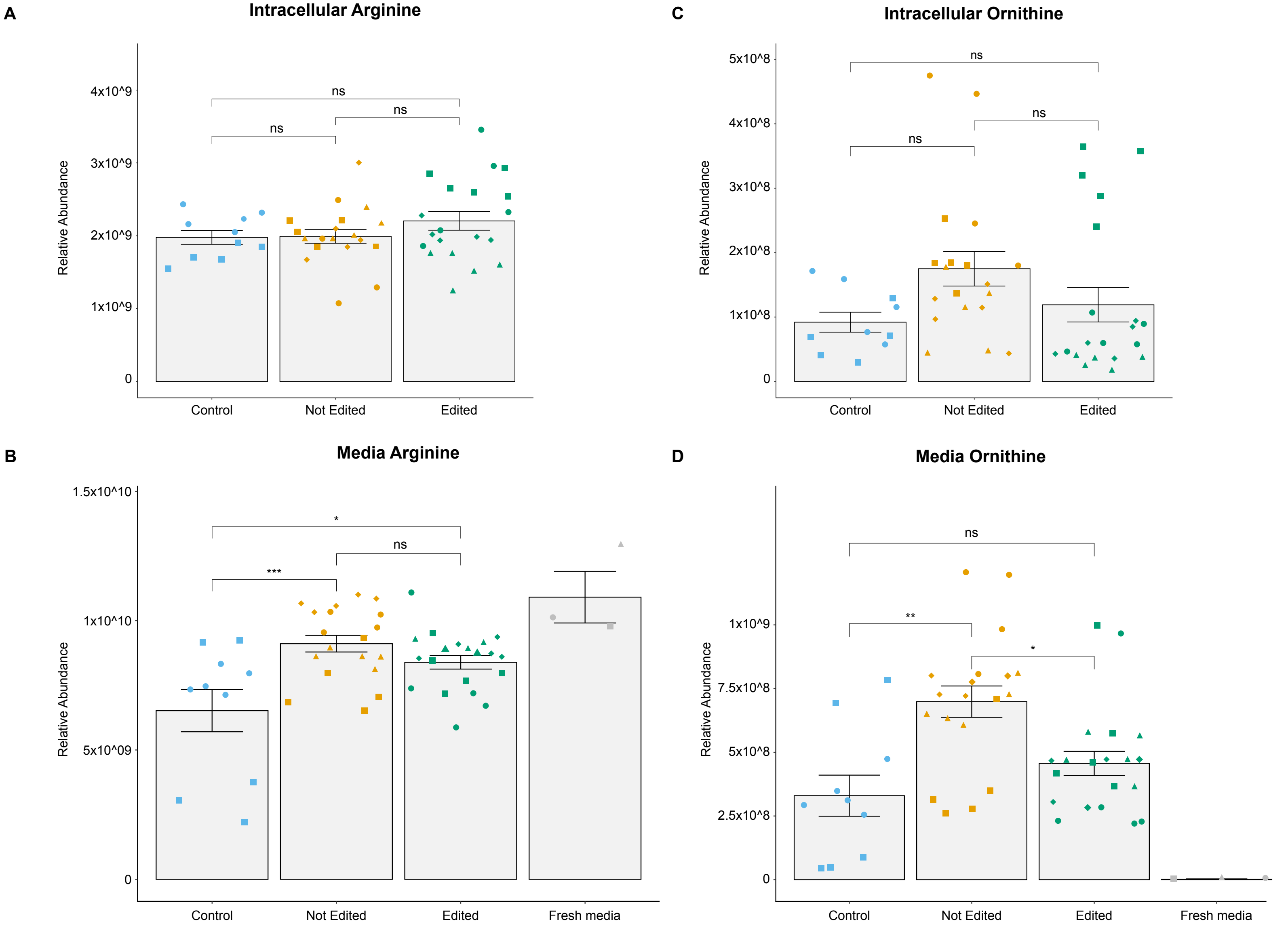

Figure S3: Metabolomic analysis of hiPSC-derived hepatocyte-like cells.
(A-D) Relative abundance of arginine (A-B) and ornithine (C-D) in the cell lysate and the media by LC-MS. Each shape represents independent differentiation batches (circle, square, diamond, triangle). We employed day-18 hiPSC-derived hepatocyte-like cells from two different probands. We analyzed two independently edited hiPSC lines per proband (four biological replicates), two not edited independent hiPSC lines per proband (four biological replicates), and HEL24.3 as a control (two biological replicates). We processed five technical replicates of each sample in the LC-MS. Relative abundance is the absolute abundance value normalized to the sum of all metabolites. Data are represented as the mean ± SEM. Statistical significance based on Tukey test; p>0.05 (ns, not significant), p < 0.05 (*), p < 0.01 (**), p < 0.001 (***), p < 0.0001 (****).

Figure S4

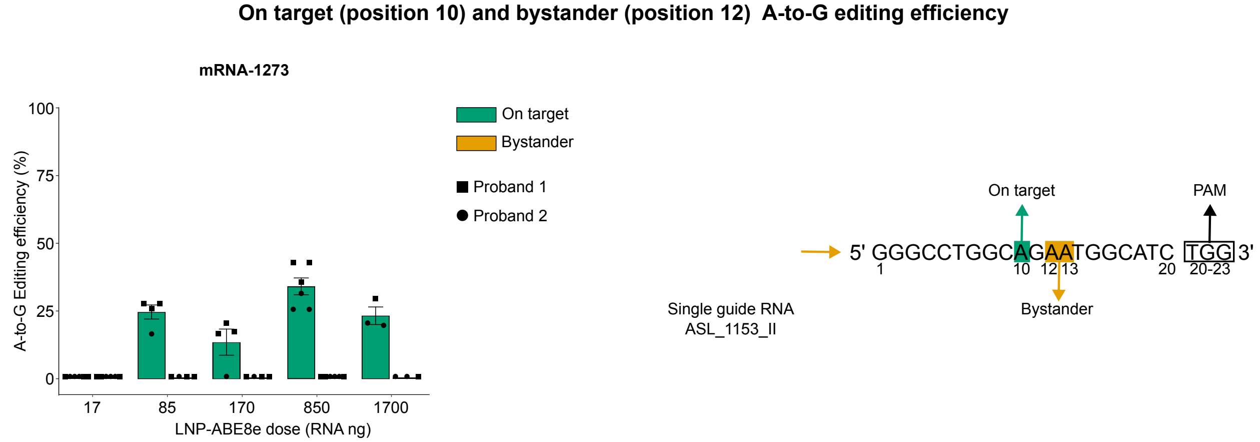

Figure S4: On target and bystander A-to-G editing efficiency using an alternative sgRNA.
On target (green) and bystander (orange) A-to-G editing efficiency. We employed primary fibroblasts from two different individuals. We independently treated these fibroblasts in triplicates (n=6) with five different doses (17 to 170 ng RNA) of the lipid nanoparticle formulation mRNA-1273 for the delivery of ABE8e together with the alternative sg ASL_1153_II. One week after the treatment, we estimated the on-target A-to-G editing efficiency by analyzing the Sanger sequence data through EditR62. Data are represented as the mean ± SEM, each data point is individually represented.

Figure S5

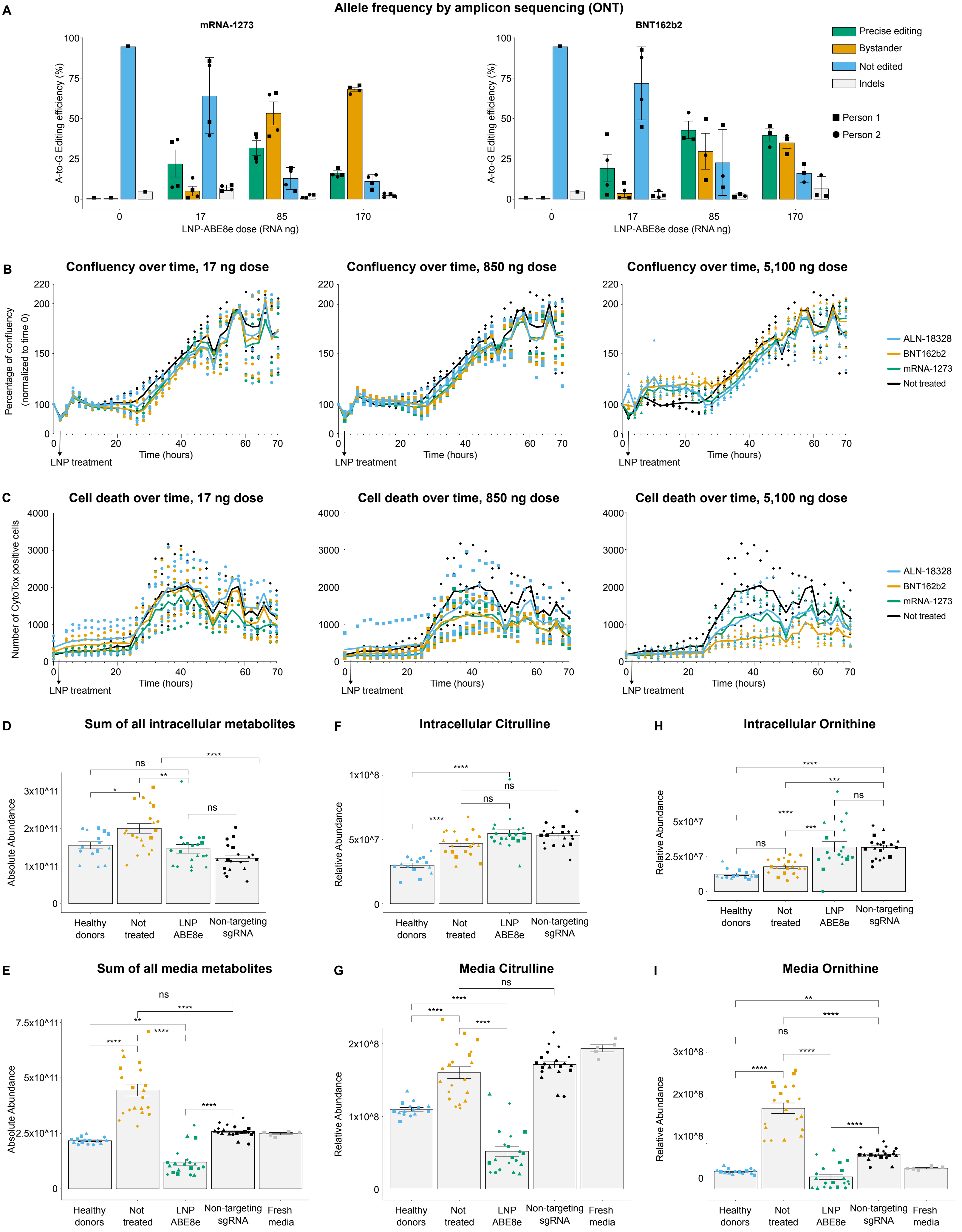
A
Allele frequency by amplicon sequencing (ONT)
mRNA-1273
BNT162b2
A-to-G Editing efficiency (%)
LNP-ABE8e dose (RNA ng)
Precise editing
Bystander
Not edited
Indels
Person 1
Person 2
B
Confluency over time, 17 ng dose
Confluency over time, 850 ng dose
Confluency over time, 5,100 ng dose
Percentage of confluency (normalized to time 0)
Time (hours)
LNP treatment
ALN-18328
BNT162b2
mRNA-1273
Not treated
C
Cell death over time, 17 ng dose
Cell death over time, 850 ng dose
Cell death over time, 5,100 ng dose
Number of CytoTox positive cells
Time (hours)
LNP treatment
D
Sum of all intracellular metabolites
Absolute Abundance
F
Intracellular Citrulline
Relative Abundance
H
Intracellular Ornithine
Relative Abundance
Healthy donors
Not treated
LNP ABE8e
Non-targeting sgRNA
E
Sum of all media metabolites
Absolute Abundance
G
Media Citrulline
Relative Abundance
I
Media Ornithine
Relative Abundance
Fresh media

Figure S5: Detailed toxicity profile, allele frequency by amplicon sequencing (ONT), and metabolomic analysis of proband primary fibroblasts after LNP-ABE8e treatment.
(A) We independently treated primary fibroblasts from two different probands in duplicates (n=4) with three different doses (17, 85, and 170 ng RNA) of two types of lipid nanoparticles (mRNA-1273 and BNT162b2) carrying the ABE8e mRNA cassette and the ASL targeting sgRNA. One week after the treatment, we estimated the allele frequency by amplicon long-read sequencing technology from Oxford Nanopore Technologies (ONT). We analyzed the data using CRISPResso2. We first filtered out those reads with less than 50% identity to the genome of reference and also those with less than 1% representation, which reduces the noise introduced by sequencing errors. We then quantified the reads in groups: "Precise Editing" refers to the alleles containing only the desired A-to-G edit on base 9 of the protospacer; "Bystander" refers to any allele containing one or more undesired A-to-G on the protospacer; "Not Edited" refers to the alleles with the unchanged proband sequence; "Indels" refers to any allele containing an insertion or deletion. Data are represented as the mean ± SEM. **(B)** We independently treated primary fibroblasts from two different probands in duplicates (n=4) with three different doses (17, 850, and 5,100 ng RNA) of three types of lipid nanoparticles ABE8e (mRNA-1273, BNT162b2, and ALN-18328). We followed the fibroblast populations for 70 hrs, taking pictures in the Incucyte® every 2 hrs. The lipid nanoparticle ABE8e treatment was applied at time 2 hrs. The solid lines represent the mean confluency of each treatment, each data point is individually expressed. **(C)** In the same setting as the previous graph, we assessed the number of dead cells, estimated by the CytoTox dye. The solid lines represent the mean number of CytoTox-positive cells for each treatment, each data point is individually expressed. **(D-E)** The sum of the absolute abundance of all the metabolites detected by LC-MS in the cell lysate and the media. Each shape represents independent lipid nanoparticle treatments (circle, square, diamond, triangle). We employed primary fibroblasts from two different probands. We independently treated these fibroblasts in duplicates (four biological replicates) with 85 ng RNA of the mRNA-1273 lipid nanoparticle ABE8e plus the variant-targeting sgRNA (LNP-ABE8e), or with vehicle (not treated), or with mRNA-1273 lipid nanoparticle ABE8e containing the sgRNA Site_16 targeting an unrelated locus (non-targeting sgRNA). As a control, we used fibroblasts coming from three healthy donors of different genders and ages (healthy donors) (three biological replicates). Two weeks after the treatment, we analyzed the metabolite content of each condition in five technical replicates. The sum of the absolute abundance of all the metabolites in each sample was employed as a normalization to calculate the relative abundance of individual metabolites. Data are represented as the mean ± SEM. **(F-I)** Relative abundance of intracellular and media citrulline **(F-G)** and ornithine **(H-I)** measured by LC-MS in the same samples described above. Relative abundance is the absolute abundance value normalized to the sum of all metabolites. Data are represented as the mean ± SEM. Statistical significance based on Tukey test; p>0.05 (ns, not shown), p < 0.05 (*), p < 0.01 (**), p < 0.001 (***), p < 0.0001 (****).

Figure S6

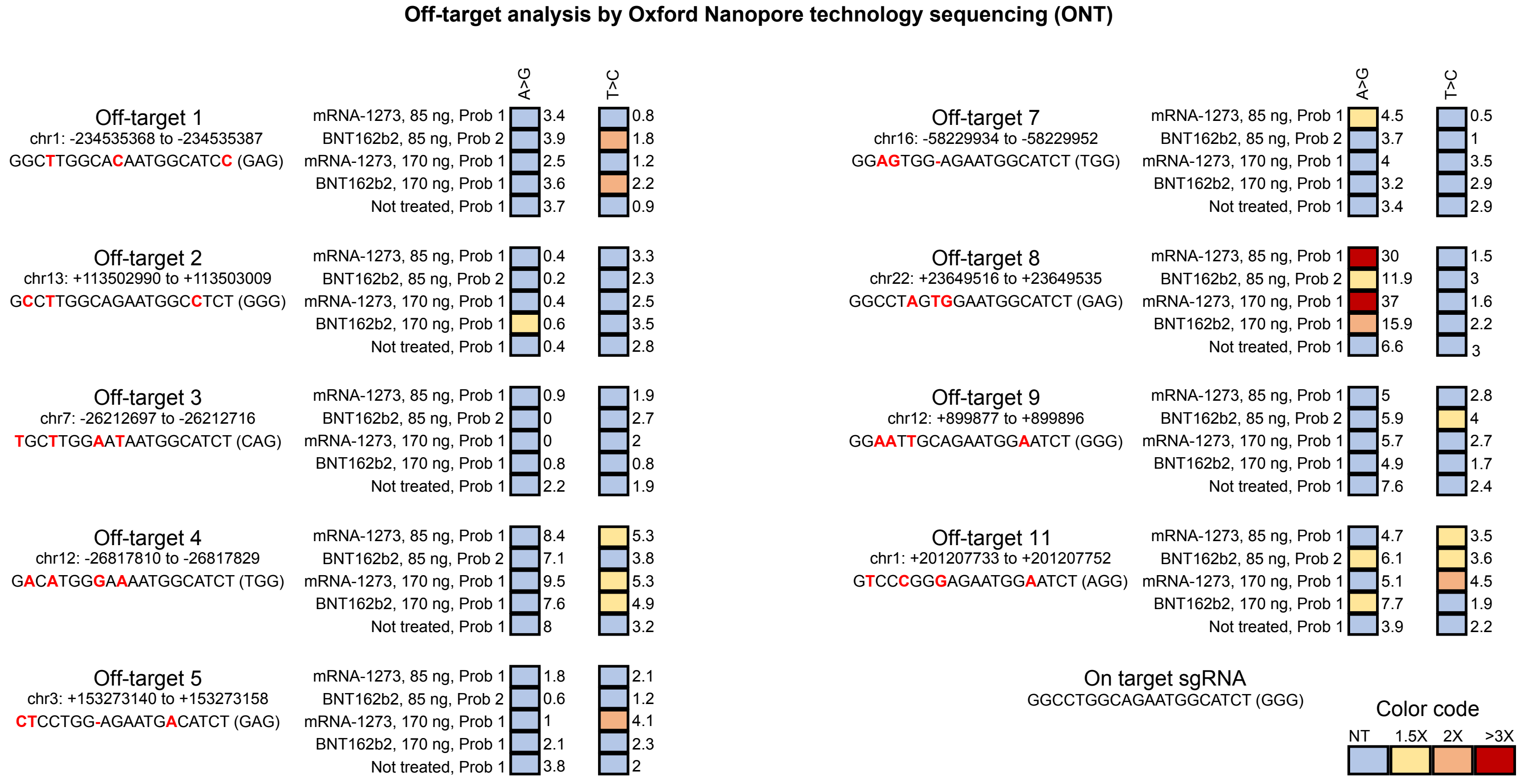

Figure S6: Analysis of the off-target activity through Oxford Nanopore Technology (ONT) sequencing.
To investigate the off-target effect in more detail, we generated a new list of 11 *in silico* predicted off-target sites using the software by IDT, CRISPOR, and Bencling. Then, we selected one non-treated fibroblast sample and four LNP-treated that showed a high on-target editing efficiency and amplified through PCR the 11 off-target sites in each one. We sequenced each DNA sample by ONT and analyzed the individual reads employing the CRISPResso2 software. The amplicon sizes were between 1400 nt and 2300 nt. We filtered out those reads that did not align with the genome of reference with at least 50% identity, but we kept all aligned reads, even those with low representation. This increases the sensitivity for rare events but also the noise in the analysis. We first compared the number of insertions and deletions in each treated sample to the non-treated one and saw no difference in that value. After that, we quantified all the adenine deamination activity on each of the nine off-target sites (as the sequencing of the off-target 6 and 10 filed due to highly repetitive flanking regions). When the putative off-target site falls on the positive strand of the locus, the base editing appears as an A>G change. When it occurs on the negative strand, the editing appears as a T>C change. Here we plotted the percentage of all aligned reads that presented any A>G (left) or T>C (right) conversion on the 20 nt region of each of the nine off-target sites. For each of the off-target sites, we noted the chromosomal location and the mismatches (in red) compared to the on-target sgRNA. The color of each square represents the fold change on A>G or T>C conversion percentage compared to the value of the non-treated sample for each of the off-target regions. To the right side of each square, we added the A>G or T>C conversion percentage for each sample. The ONT has some associated errors, and even the non-treated samples show variable levels of A>G and T>C change. As the ONT sequencing quality is partly related to the characteristics of each locus, for each off-target site we compared the conversion percentage of each treated sample to the conversion percentage of the corresponding non-treated sample.

Supplemental Tables

Table S1

sgRNA: ASL_1153	Software for prediction (order in the top 3 predictions)	Sequence	PAM	#MM	Gene	Locus	Editing
**On-target**		**GGCCTGGCAGAATGGCATCT**	**GGG**	**0**	**exon: *ASL***	**chr7:- 66092556-66092575**	**positive**
Off-target	IDT (1st)	AGCCTGG-AGAATGGCATCT	AAG	2	intergenic	chr2:+ 31113350-31113368	negative
Off-target	IDT (2nd) / Benchling (2nd)	TGCTTGTCAGAATGGCATCT	TGG	3	intergenic	chr2:- 29523682-29523701	negative
Off-target	IDT (3rd)	GGGCGGGCAGAATGACATCT	GGG	3	exon: *C1orf94*	chr1:- 34177482-34177501	negative
Off-target	CRISPOR (1st)	GGCCTGACAGAATGAAATCT	TGG	3	intron: *SCARB2*	chr4:+ 77128950-77128972	negative
Off-target	CRISPOR (2nd)	GGCCCAGCAAAATGACATCT	TGG	4	intergenic	chr4:- 7113139-7113161	negative
Off-target	Benchling (1st)	GGCCTGGCTGGATGGCATCT	TGG	2	intergenic	chr8:+ 142517669-142517688	negative
Off-target	Benchling (3rd)	TGCCTTGCACAATGGCATCT	TAG	3	intergenic	chr2:+ 184202763-184202782	negative

Table S1: off-target analysis by Sanger sequencing in hepatocyte-like cells and proband primary fibroblasts
Table S1 shows the *in silico* predicted off-target sites that were sequenced to check unwanted ABE-induced A•T to G•C mutations (off-target). The bases in red are the mismatches between each off-target sequence and the sgRNA ASL_1153. The number of mismatches is represented as #MM. We tested the presence of off-targets in the following hiPSC lines: Prob1-EDIT1, Prob1-EDIT2, Prob2-EDIT1, and Prob2-EDIT2. We also tested the presence of off-targets in the four fibroblast populations independently treated with 85ng of the mRNA-1273 LNP formulation carrying the ABE8e RNA vector, and the ASL_1153 sgRNA. We did not detect off-target activity in any of the seven loci analyzed in the four hiPSC or the four fibroblast populations.

Table S2

sgRNA: ASL_1153	Software for prediction (order of likelihood)	Sequence	PAM	#MM	Gene	Locus
**On-target**		**GGCCTGGCAGAATGGCATCT**	**GGG**	**0**	**exon: *ASL***	chr7:-66092556 to -66092575
ONT Off-target 1	Benchling (4th)	GGCTTGGCACAATGGCATCC	GAG	3	intergenic	chr1:-234535368 to 234535387
ONT Off-target 2	Benchling (5th)	GCCTTGGCAGAATGGCCTCT	GGG	3	intergenic	chr13:+113502990 to +113503009
ONT Off-target 3	Benchling (6th)	TGCTTGGAATAATGGCATCT	CAG	4	exon: *CBX3*	chr7:-26212697 to -26212716
ONT Off-target 4	Benchling (7th) / CRISPOR (5th)	GACATGGGAAAATGGCATCT	TGG	4	intergenic	chr12:-26817810 to -26817829
ONT Off-target 5	IDT (4th)	CTCCTGG-AGAATGACATCT	GAG	4	intergenic	chr3:+153273140 to +153273158
ONT Off-target 6	IDT (5th)	AGCTTGG-ATAATGGCATCT	GAG	4	intergenic	chr8:+129331504 to +129331522
ONT Off-target 7	IDT (6th)	GGAGTGG-AGAATGGCATCT	TGG	3	intergenic	chr16:-58229934 to -58229952
ONT Off-target 8	IDT (7th)	GGCCTAGTGGAATGGCATCT	GAG	3	*exon: *ASLP1*	chr22:+23649516 to +23649535
ONT Off-target 9	CRISPOR (3rd)	GGAATTGCAGAATGGAATCT	GGG	4	intron: *WNK1*	chr12:+899877 to +899896
ONT Off-target 10	CRISPOR (6th)	AGCCTGGCGGAAAGGAATCT	GGG	4	intron: *TAF2*	chr8:-119829280 to -119829299
ONT Off-target 11	CRISPOR (7th)	GTCCCGGGAGAATGGAATCT	AGG	4	exon: *IGFN1*	chr1:+201207733 to +201207752

Table S2: off-target sites for Oxford Nanopore technology sequencing analysis
Table S2 shows the *in silico* predicted off-target sites that were sequenced through Oxford Nanopore technology to check unwanted ABE-induced A•T to G•C mutations (off-target). The bases in red are the mismatches between each off-target sequence and the sgRNA ASL_1153. The number of mismatches is represented as #MM. We tested the presence of off-targets in four different LNP-treated fibroblast populations, the results are expressed in Figure S6.
**ASL* pseudogene ASLP1 (Refseq# NG_002637.6).

Table S3

Name	Sequence	Function	Comments	Vendor
**Single guide RNA**				
sgRNA: ASL_1153	mG*mG*mC*rCrUrGrGrCrArGrArArUrGrGrCrArUrCrUrGrUrUrUrUrArGrArGrCrUrArGrArArArUrArGrCrArArGrUrUrArArArArUrArArGrGrCrUrArGrUrCrCrGrUrUrArUrCrArArCrUrUrGrArArArArArGrUrGrGrCrArCrCrGrArGrUrCrGrGrUrGrCmU*mU*mU*rU	Target the ASL variant in chromosome 7	RNA oligo	IDT
sgRNA: ASL_1153_II	mG*mG*mG*rCrCrUrGrGrCrArGrArArUrGrGrCrArUrCrGrUrUrUrUrArGrArGrCrUrArGrArArArUrArGrCrArArGrUrUrArArArArUrArArGrGrCrUrArGrUrCrCrGrUrUrArUrCrArArCrUrUrGrArArArArArGrUrGrGrCrArCrCrGrArGrUrCrGrGrUrGrCmU*mU*mU*rU	Target the ASL variant in chromosome 7	RNA oligo	IDT
sgRNA: Site_16	mG*mG*mG*rArArUrArArArUrCrArUrArGrArArUrCrCrGrUrUrUrUrArGrArGrCrUrArGrArArArUrArGrCrArArGrUrUrArArArArUrArArGrGrCrUrArGrUrCrCrGrUrUrArUrCrArArCrUrUrGrArArArArArGrUrGrGrCrArCrCrGrArGrUrCrGrGrUrGrCmU*mU*mU*rU	Target the site 16 locus in chromosome 1. We used it as a non-targeting sgRNA control, given that it targets a locus unrelated to *ASL*.	RNA oligo	IDT
**PCR primers**				
*ASL*_Fw	GAGCTCAGGAATGGGTGCAA	Amplifies the genomic region around the pathogenic variant	DNA oligo	IDT
*ASL*_Rv	TCGCGCCCACTTTATTAGGG	Amplifies the genomic region around the pathogenic variant	DNA oligo	IDT
OT 1 Fw	CCTTGTAGGAGCAGTACCACC	Amplifies the genomic region containing a possible off-target	DNA oligo	IDT
OT 2 Fw	GCCCCGTTCAAGATCACAGA	Amplifies the genomic region containing a possible off-target	DNA oligo	IDT
OT 3 Fw	CTAGGCTGTCTTGGGGTGTG	Amplifies the genomic region containing a possible off-target	DNA oligo	IDT
OT 4 Fw	ACAGTTGTCAGCATAGCCCC	Amplifies the genomic region containing a possible off-target	DNA oligo	IDT
OT 5 Fw	GGCCACCACTTAGTGACTCC	Amplifies the genomic region containing a possible off-target	DNA oligo	IDT
OT 6 Fw	ACGGGAGAAGTGAGGAATGAC	Amplifies the genomic region containing a possible off-target	DNA oligo	IDT
OT 1 Rv	CCTTGTAGGAGCAGTACCACC	Amplifies the genomic region containing a possible off-target	DNA oligo	IDT
OT 2 Rv	GCCCCGTTCAAGATCACAGA	Amplifies the genomic region containing a possible off-target	DNA oligo	IDT
OT 3 Rv	CTAGGCTGTCTTGGGGTGTG	Amplifies the genomic region containing a possible off-target	DNA oligo	IDT
OT 4 Rv	ACAGTTGTCAGCATAGCCCC	Amplifies the genomic region containing a possible off-target	DNA oligo	IDT
OT 5 Rv	GGCCACCACTTAGTGACTCC	Amplifies the genomic region containing a possible off-target	DNA oligo	IDT
OT 6 Rv	ACGGGAGAAGTGAGGAATGAC	Amplifies the genomic region containing a possible off-target	DNA oligo	IDT
EBNA 1 Fw	ATCGTCAAAGCTGCACACAG	Amplifies the EBNA-1 region from the reprogramming plasmids in hiPSC	DNA oligo	Sigma
EBNA 1 Rv	CCCAGGAGTCCCAGTAGTCA	Amplifies the EBNA-1 region from the reprogramming plasmids in hiPSC	DNA oligo	Sigma
OriP Fw	TTCCACGAGGGTAGTGAACC	Amplifies the OriP region from the reprogramming plasmids in hiPSC	DNA oligo	Sigma
OriP Rv	TCGGGGGTGTTAGAGACAAC	Amplifies the OriP region from the reprogramming plasmids in hiPSC	DNA oligo	Sigma
ONT_OT_1_Fw	TGGCCTCACTGACGAGTTTC	Amplifies the genomic region containing a possible off target	DNA oligo	IDT
ONT_OT_1_Rv	CTTTGTCGGCCTCTCATGGT	Amplifies the genomic region containing a possible off target	DNA oligo	IDT
ONT_OT_2_Fw	GGCGGTGTTACTGGAGACAA	Amplifies the genomic region containing a possible off-target	DNA oligo	IDT
ONT_OT_2_Rv	TCTTTTGTGCGGTGTCTGGA	Amplifies the genomic region containing a possible off-target	DNA oligo	IDT
ONT_OT_3_Fw	TGGCTGTCGGTTGACATGAA	Amplifies the genomic region containing a possible off-target	DNA oligo	IDT
ONT_OT_3_Rv	GTCTATCATCACACTGAGCCCT	Amplifies the genomic region containing a possible off-target	DNA oligo	IDT
ONT_OT_4_Fw	GTGGCGAAAGGGAACTCAGA	Amplifies the genomic region containing a possible off-target	DNA oligo	IDT
ONT_OT_4_Rv	CCCATGCGAAGAATGAGCCT	Amplifies the genomic region containing a possible off-target	DNA oligo	IDT
ONT_OT_5_Fw	AGAGACGGCTCTTTCCCTCT	Amplifies the genomic region containing a possible off-target	DNA oligo	IDT
ONT_OT_5_Rv	GTGATGAGCAGCTAGGGAGC	Amplifies the genomic region containing a possible off-target	DNA oligo	IDT
ONT_OT_6_Fw	AACACTTCCAGCTTGCCTGA	Amplifies the genomic region containing a possible off-target	DNA oligo	IDT
ONT_OT_6_Rv	ATTCCTGGTGGAGCATTCGT	Amplifies the genomic region containing a possible off-target	DNA oligo	IDT
ONT_OT_7_Fw	ACCGCGAAACAAAATCCTGG	Amplifies the genomic region containing a possible off-target	DNA oligo	IDT
ONT_OT_7_Rv	TGCCCTTTTTCCCCAACAGT	Amplifies the genomic region containing a possible off-target	DNA oligo	IDT
ONT_OT_8_Fw	CAGGGCAGAGGCTGTGATAG	Amplifies the genomic region containing a possible off-target	DNA oligo	IDT
ONT_OT_8_Rv	GGGGTCCTCAAACGAAACCT	Amplifies the genomic region containing a possible off-target	DNA oligo	IDT
ONT_OT_9_Fw	CACATTAGGGCCAGTTCATGC	Amplifies the genomic region containing a possible off-target	DNA oligo	IDT
ONT_OT_9_Rv	TAGTCTCCCAACCCGCCTTA	Amplifies the genomic region containing a possible off-target	DNA oligo	IDT
ONT_OT_10_Fw	TAGGTGGGAACAGGGCTGAA	Amplifies the genomic region containing a possible off-target	DNA oligo	IDT
ONT_OT_10_Rv	ACTGCGGTAATCTAGGGGAGA	Amplifies the genomic region containing a possible off-target	DNA oligo	IDT
ONT_OT_11_Fw	AACTGTGGGATGCTCGACTG	Amplifies the genomic region containing a possible off-target	DNA oligo	IDT
ONT_OT_11_Rv	GGCCCTGGAAGTTCTGATCC	Amplifies the genomic region containing a possible off-target	DNA oligo	IDT
**qPCR primers**				
ASL Fw	CTCTCAACAGCATGGATGCCAC	quantifies ASL expression in qPCR	DNA oligo	Sigma
ASL Rv	CTTGGTGCAGTAGAGGATGAGG	quantifies ASL expression in qPCR	DNA oligo	Sigma
AFP Fw	CGCTGCAAACGATGAAGCAAG	quantifies AFP expression in qPCR	DNA oligo	Sigma
AFP Rv	AATCTGCAATGACAGCCTCAAG	quantifies AFP expression in qPCR	DNA oligo	Sigma
Albumin Fw	GGAAAAGTGGGCAGCAAATGT	quantifies Albumin expression in qPCR	DNA oligo	Sigma
Albumin Rv	GGTTCAGGACCACGGATAGA	quantifies Albumin expression in qPCR	DNA oligo	Sigma
HNF1a Fw	GGGCTTCTTGGACAACCTTTTCA	quantifies HNF1a expression in qPCR	DNA oligo	Sigma
HNF1a Rv	CGTATGGACACCCGGCTCAT	quantifies HNF1a expression in qPCR	DNA oligo	Sigma
CycloG Fw	TCTTGTCAATGGCCAACAGAG	quantifies CycloG expression in qPCR	DNA oligo	Sigma
CycloG Rv	GCCCATCTAAATGAGGAGTTG	quantifies CycloG expression in qPCR	DNA oligo	Sigma
Serpina1 Rv	AGCTGGCACACCAGTCCAAC	quantifies Serpina expression in qPCR	DNA oligo	Sigma
Serpina1 Fw	TGGCTGGTTGAGGGTACGGA	quantifies Serpina expression in qPCR	DNA oligo	Sigma
APOC3 Fw	CCGCCAAGGATGCACTGAG	quantifies APOC3 expression in qPCR	DNA oligo	Sigma
APOC3 Rv	CTCCAGTAGTCTTTCAGGGAACT	quantifies APOC3 expression in qPCR	DNA oligo	Sigma
APOA2 Fw	ATGTGTGGAGAGCCTGGTTTCTCA	quantifies APOA2 expression in qPCR	DNA oligo	Sigma
APOA2 Rv	AAGCTCTGGGCTCTTGACCTTCT	quantifies APOA2 expression in qPCR	DNA oligo	Sigma
OCT4 Fw	TTGGGCTCGAGAAGGATGTG	quantifies OCT4 expression in qPCR	DNA oligo	Sigma
OCT4 Rv	GTGAAGTGAGGGCTCCCATA	quantifies OCT4 expression in qPCR	DNA oligo	Sigma
SOX2 Fw	GCCCTGCAGTACAACTCCAT	quantifies SOX2 expression in qPCR	DNA oligo	Sigma
SOX2 Rv	CTCAGCCTCCAGCAGATGC	quantifies SOX2 expression in qPCR	DNA oligo	Sigma
Nanog Fw	TGCCCTGCTGCGAGTAGGA	quantifies Nanog expression in qPCR	DNA oligo	Sigma
Nanog Rv	TAGATTTCATTCTCTGGTTCTGG	quantifies Nanog expression in qPCR	DNA oligo	Sigma
**Primers for cloning**				
ABE8e-Fw	CTCAACTTTGGCAGATCTGAATTCCTGCAGCCCGGGATCCACAGCCACCATGAAACGGACAGCCGACGGAAGCGAG	Cloning ABE8e ORF into IVT T3 backbone	DNA oligo	Sigma
ABE8e-Rv	CTTGAGGCTGGTTTAGTGGTAACCAGATCCGCGGCCGCTCACCTACTTAGACTTTCCTCTTCTTCTTGGGCTCG	Cloning ABE8e ORF into IVT T3 backbone	DNA oligo	Sigma

Table S3: primers and sgRNAs

Table S3 shows the sgRNA ASL_1153 employed to target the ASL c.1153C>T pathogenic variant, and the sgRNA Site_16 used as a control guide targeting a locus unrelated to ASL. The table also shows the DNA primers for PCR and qPCR. The sequences are notated in 5'-to-3' orientation. In the "single guide RNA section", the protospacer is underlined, and the non-underlined sequence is the canonical CRISPR-Cas9 tracrRNA. "r_" (rA, rC, rG, rU) refers to ribonucleic bases. "m_*" (mC*, mA*, mG*, mU*) refers to phosphorothioated 2'-O-methyl RNA bases.

Supplemental Methods

Fibroblast culture

The skin biopsies were manually disaggregated, seeded, and cultured under glass coverslips in a 60 mm dish. The culture medium consisted of DMEM (Sigma, 6546) supplemented with 20% fetal bovine serum (FBS, Life Technologies, 10106-169), 1X GlutaMAX (Life Technologies, 35050-038), and 1% penicillin-streptomycin (Life Technologies, 15140-122) until the fibroblast cells formed a monolayer, after which the cells were passaged with TrypLETM Select Enzyme (Thermo Fisher Scientific; 12563029). After passaging the fibroblasts from the plates containing the skin biopsies, they were cultured in DMEM supplemented with 10% FBS and 1X GlutaMAX.

Incucyte and toxicity experiments

We followed the fibroblast population using the Incucyte® Live-Cell Analysis System, which automatically images the cells to assess their confluency and the number of dead cells, stained by the CytoTox membrane integrity dye (Sartorius; Cat. No. 4633). The fibroblasts were plated on 24-well plates and cultured with DMEM FBS 10%. We programmed the Incucyte® to take 16 pictures per well every 2 hrs, from time 0 to 70 hrs. Either different concentrations of lipid nanoparticles or an equal volume of PBS was added into each well at the 2 hrs time point. The confluence was calculated using the Incucyte® software and normalized to time 0. The absolute number of green positive (dead) cells was counted using the same software.

Direct argininosuccinate lyase activity assay

Fibroblasts were cultured in 100 mm plates until 80 % confluency, the cells were then washed with PBS, collected using a cell scraper, and centrifuged at 200g for 4 minutes. The supernatant was discarded, and the cell pellets were stored at -80°C. Protein samples were extracted from frozen fibroblast pellets by lysis with a buffer containing PBS, 0.1% TritonX-100 (Sigma-Aldrich, X100), and protease inhibitors (Roche, 04693124001), centrifuging 10,000 RCF for 10 minutes at + 4°C. The protein concentration in the supernatant was measured by PierceTM BCA Protein assay kit (Thermo Scientific; 23227). 0.6 mg/ml of total protein lysate was incubated in a physiological buffer consisting of 100 mM Tris-HCl (pH 7.2) and 0.3 mM argininosuccinate disodium salt (73097; Sigma-Aldrich) for 15 minutes at +37°C and 5 minutes at +95°C. The incubation was performed in the S1000 Thermal Cycler (Biorad) and the final volume per each reaction was 120 ul. The ASL enzyme activity was estimated by measuring the total amount of fumarate (in nanomoles) that formed during the incubation using the Fumarate Assay kit from Sigma-Aldrich (MAK060). The amount of fumarate in each reaction was measured in duplicates, loading 50 ul of the reaction supernatant into 96-well plate wells for a spectrophotometric measurement with the EnSpire® Multimode Plate Reader (PerkinElmer) at 450 nm. The unspecific reaction background was determined by a parallel reaction where the incubation order was reversed (5 minutes at +95°C and 15 minutes at +37°C). This background read was subtracted from the final result.

HepG2 cell line

HepG2 (Knowles BB, Aden DP. US Patent 4,393,133 dated Jul 12, 1983) is a commercial cell line isolated from a hepatocellular carcinoma of a 15-year-old, white male. The HepG2 line presents a high expression of AFP, HNF1α, SERPINA1, Albumin, APOA2, and APOC31; hence it was used as a positive control for the qPCR of the mentioned genes.

The cells were cultured in 12 well plates with no coating. The media consisted of DMEM (Sigma, 6546) supplemented with 10% fetal bovine serum (FBS, Life Technologies, 10106-169) inactivated for 30min at 56°C, 1X GlutaMAX (Life Technologies, 35050-038), and 1% penicillin-streptomycin (Life Technologies, 15140-122).

Sample preparation for metabolomic analysis

Hepatocyte-like cells and primary fibroblasts were cultured in 12 well plates with 0.75 mL of their respective media. Hepatocyte-like cells were harvested at day 18 of their differentiation protocol, fibroblasts were harvested one day after reaching confluency.

For harvesting, all the media (which was in contact with the cells for 24 hrs) in the well was collected and placed in a tube on ice. Then, we washed the cells once with cold PBS and added 255 µl of +4°C extraction buffer (Acetonitrile/dH2O 80:20) on top of the cells. All the cells in the well were detached and homogenized using a pipette and then transferred into a tube on ice. The samples were vortexed for 10 sec at full speed. At the same time, 50 µl of each media sample was transferred into a tube with 450 µl of +4°C extraction buffer (Acetonitrile/dH2O 80:20), and then vortexed. Then, all the cell lysate samples and the diluted media samples were centrifuged (15,800 g for 10 min at +4°C). We finally transferred 100 µl of the supernatant of each sample to a SureSTART 0.3 ml glass screw top micro vial (Thermo Scientific) and stored them at -80°C for no longer than one month until the metabolomic analysis.

sgRNA design

The sgRNA ASL_1153 and ASL_1153_II were designed using the web tool Benchling (https://benchling.com). Integrated DNA Technologies manufactured the sgRNA. The sequence of the sgRNA is listed in Table S3.

Determination of on-target editing, Sanger sequencing

We extracted genomic DNA from a bulk population of millions of cells, amplified the genomic locus around the *ASL* variant by PCR, purified and Sanger sequenced the PCR product. We analyzed on-target editing using the web tool EditR2.

Off-target analysis, Sanger sequencing

The seven most likely off-target loci for the sgRNA ASL_1153 were predicted by the web tools provided by (IDT Integrated DNA Technologies), Benchling (https://benchling.com), and CRISPOR3. See Table S1 for the top seven list of in silico predicted off-targets for the sgRNA ASL_1153. DNA samples from monoclonal edited hiPSC lines and edited bulk fibroblast populations were collected. Each of the seven loci in each sample was amplified by PCR (primers described in Table S3). The off-target editing was estimated from the Sanger sequencing data using the web tool EditR.

On-target and off-target analysis, Oxford Nanopore technology sequencing

We performed a 30-cycle PCR for each primary fibroblast DNA sample. We then purified the PCR product through electrophoresis in a 1% agarose TAE gel and column purified the gel band eluting the DNA in a concentration of 30 ng/ul. The samples were packaged according to the company guidelines (https://www.plasmidsaurus.com) and shipped them at room temperature. We obtained between 300 and 2,000 raw reads per sample and aligned them to the genome of reference and the sgRNA sequence using the CRSPResso2 software. For statistical analysis of the on-target editing efficiency, we considered all the reads that aligned with the genome of reference with at least 50% identity and had a representation equal to or above 1%, and computed the allele frequency into three categories: precisely edited reads, those that contained any bystanders or indels, and those that remained unchanged. For the statistical analysis of the off-target, we considered all reads that aligned with the genome of reference with at least 50% identity. Using CRISPResso2, we determined the A>G or T>C conversion percentages for every adenine or thymidine within the 20 nt region of each of the nine off-target sites. We then separately added all the A>G or T>C conversion percentages to create a parameter that reflects the potential ABE editing for each off-target site.

Western blot analysis

Cell samples were collected from culture plate wells by trypsinization or scraping and the cell pellets were lysed in a buffer containing PBS, 0.1 % TritonX-100, and protease inhibitors. The samples were centrifuged at 4°C for 10 minutes, 10000 g, and the supernatant was collected. The protein concentration of each sample was quantified by the Pierce BCA Protein Assay Kit (ThermoFisher, 23227). The samples were combined with 4x Laemmli Sample Buffer (Biorad, 1610746), and denatured at +95°C for 5 minutes. For the Western blot, 30 µg of total protein was seeded in each well of a Mini-Protean TGX Precast gel (Biorad, 456-8034). Primary antibodies: anti-ASL antibody produced in rabbit (Sigma-Aldrich, HPA016646, 1:500 dilution in 5 % milk-TBST), Human Serum Albumin antibody produced in mouse (R&D systems; MAB1455; 1:500 dilution in 5 % milk-TBST), anti-actin Antibody produced in mouse (Sigma-Aldrich; A3853; 1:1000 dilution in 5 % milk-TBST). Secondary antibodies: IRDye 800CW anti-rabbit IgG (LI-COR, 926-32213, 1:1,000 dilution in 5 % milk-TBST) and IRDye 680RD anti-mouse IgG (LI-COR, 926-68072, 1:1,000 dilution in 5 % milk-TBST). The ladder employed was Precision Plus Protein Dual Color Standards (Biorad, 1610374). After the treatment to simultaneously stain ASL, albumin, and actin, the membrane was imaged in the Odyssey (LI-COR), ASL was visible in the green channel, whereas albumin and actin were captured in the red channel.

Immunocytochemistry

HiPSCs were plated on round coverslips coated with Matrigel in 24-well plates and cultured to 50 % confluency when they were fixed. HiPSC-derived hepatocytes were differentiated on round coverslips coated with Matrigel in 24-well plates and fixed on day 18.

Cells were fixed with 4 % PFA in PBS for 15 minutes, permeabilized with 0.5 % Triton X-100 for 10 minutes, and blocked with an Ultravision blocker (Thermo Scientific) for 10 minutes. They were then incubated with primary antibodies diluted in 0.1% Tween in PBS for 24 hours at 4°C on a Stuart SSL4 seesaw rocker. Then, the plates were treated with Hoechst 33342 (Thermo Fisher Scientific), and the secondary antibodies were diluted in 0.1 % Tween in PBS in the dark at room temperature for 30 minutes on the seesaw rocker.

Primary antibodies: OCT4 (1:500 goat, polyclonal, Santa Cruz, sc-8628), TRA-1-60 (1:500 mouse, monoclonal, Thermo Fisher Scientific, MA1-023), SSEA (1:1000 mouse, monoclonal, Millipore, MAB4304), SOX17 (1:500 goat, polyclonal, R&D Systems, AF1924), α-SMA (1:500 mouse, monoclonal, Sigma, A2547), and β-tubulin III (1:500 rabbit, polyclonal, Abcam, Ab18207), AFP (1:300 rabbit, polyclonal, Agilent, A000829-2), Albumin (1:33 mouse, monoclonal, R&D Systems, MAB1455), HNF4α (1:500 rabbit, monoclonal, Cell Signalling Technology, 3113).

Secondary antibodies: Alexa Fluor 488 anti-goat (1:500 donkey, Invitrogen, A11055), Alexa Fluor 488 anti-mouse (1:500 donkey, Invitrogen, A21202) and anti-rabbit (1:500 donkey, Invitrogen, A21206), and Alexa Fluor 594 anti-mouse (1:500 donkey, Invitrogen, A21203) and anti-rabbit (1:500 donkey, Invitrogen, A21207).

Karyotyping

Samples were prepared for karyotyping as described previously[4]. 2.0 x 106 cells were suspended in a medium supplemented with 0.1 µg/mL KaryoMAX Colcemid Solution in PBS and incubated for 4 hrs at 37 °C. Cells were resuspended in 0.075 M KCl and incubated at 37 °C for 10 min. Fixative (3:1 ratio of methanol and acetic acid) was added dropwise to the cell suspension. Fixation was repeated three times before storing the samples at 4 °C until shipping. Karyotyping was performed as a service by Ambar in Barcelona, Spain.

Supplemental references

1. Rouillard, A.D., Gundersen, G.W., Fernandez, N.F., Wang, Z., Monteiro, C.D., McDermott, M.G., and Ma'ayan, A. (2016). The harmonizome: a collection of processed datasets gathered to serve and mine knowledge about genes and proteins. Database 2016, baw100. 10.1093/database/baw100.
2. Kluesner, M.G., Nedveck, D.A., Lahr, W.S., Garbe, J.R., Abrahante, J.E., Webber, B.R., and Moriarity, B.S. (2018). EditR: A Method to Quantify Base Editing from Sanger Sequencing. CRISPR J 1, 239–250. 10.1089/crispr.2018.0014.
3. Concordet, J.P., and Haeussler, M. (2018). CRISPOR: Intuitive guide selection for CRISPR/Cas9 genome editing experiments and screens. Nucleic Acids Res 46, W242–W245. 10.1093/nar/gky354.
4. Howe, B., Umrigar, A., and Tsien, F. (2014). Chromosome preparation from cultured cells. J Vis Exp, e50203–e50203. 10.3791/50203.
